# Molecular Effects of FDA-Approved Multiple Sclerosis Drugs on Glial Cells and Neurons of the Central Nervous System

**DOI:** 10.3390/ijms21124229

**Published:** 2020-06-13

**Authors:** Kim M. A. De Kleijn, Gerard J. M. Martens

**Affiliations:** 1 Department of Molecular Animal Physiology, Donders Institute for Brain, Cognition and Behaviour, Centre for Neuroscience, Faculty of Science, Radboud University, 6525 AJ Nijmegen, The Netherlands; k.dekleijn@science.ru.nl; 2 NeuroDrug Research Ltd., 6525 HP Nijmegen, The Netherlands

**Keywords:** fingolimod, dimethyl fumarate, teriflunomide, glatiramer acetate, interferon-β, microglia, astrocyte, neuron, oligodendrocyte, multiple sclerosis drug action

## Abstract

Multiple sclerosis (MS) is characterized by peripheral and central inflammatory features, as well as demyelination and neurodegeneration. The available Food and Drug Administration (FDA)-approved drugs for MS have been designed to suppress the peripheral immune system. In addition, however, the effects of these drugs may be partially attributed to their influence on glial cells and neurons of the central nervous system (CNS). We here describe the molecular effects of the traditional and more recent FDA-approved MS drugs Fingolimod, Dimethyl Fumarate, Glatiramer Acetate, Interferon-β, Teriflunomide, Laquinimod, Natalizumab, Alemtuzumab and Ocrelizumab on microglia, astrocytes, neurons and oligodendrocytes. Furthermore, we point to a possible common molecular effect of these drugs, namely a key role for NFκB signaling, causing a switch from pro-inflammatory microglia and astrocytes to anti-inflammatory phenotypes of these CNS cell types that recently emerged as central players in MS pathogenesis. This notion argues for the need to further explore the molecular mechanisms underlying MS drug action.

## 1. Introduction

Multiple sclerosis (MS) is an inflammatory disease of the central nervous system (CNS) characterized by oligodendrocyte pathology, microgliosis, astrogliosis, alterations of the blood–brain barrier (BBB), demyelination and neurodegeneration, and an exacerbating infiltration of both innate and adaptive immune cells into the brain [1,2]. MS is a complex disease with a large heterogeneity in MS lesions [3,4]. Furthermore, the non-lesioned white- and grey-matter regions in MS brains are different from those in healthy individuals [2,3]. For quite some time, the dysregulation of the peripheral immune system, causing immune cells infiltrating the CNS, autoreactivity against myelin sheath components and secondary BBB dysfunction, has been considered to be the primary cause of MS CNS pathology, defined as the outside-in hypothesis [5]. However, more recent research on MS and other neurodegenerative diseases has indicated a central role for a distinct type of macrophage found in the CNS, the microglia [6,7]. The hypothesis in which MS pathology is first and foremost caused by CNS-intrinsic factors, subsequently leading to the infiltration of peripheral immune cells via a leaking BBB, represents the inside-out model [8,9], which is supported by pathological evidence showing the absence of peripheral immune cells in newly forming MS lesions [10].

Because the outside-in model has been the norm for a long time, the currently available MS drugs approved by the Food and Drug Administration (FDA) have been mainly designed to target various cell types within the peripheral immune system [11] and most drug-impact studies have been directed towards their peripheral effects on the cells of the adaptive immune system [12]. However, it is likely that the MS drugs also affect (innate) CNS cells and the molecular cascades associated with neuroinflammation, since most genes that are dysregulated in MS-peripheral immune cells are also expressed in microglia [13]. MS drug effects on CNS pathology have been mostly studied in humans and animals on the basis of the clinical features of disease progression, magnetic resonance imaging (MRI) measures, and blood or cerebrospinal fluid (CSF) levels of biomarkers for demyelination and neuronal degeneration [14,15,16]. For this reason, we set out to review studies assessing at the molecular level, the effects of MS drugs on the pathways operational in CNS cells.

Molecular effects on cell types in the CNS have been reviewed for a number of FDA-approved MS drugs, such as Fingolimod (FTY720; Gilenya), Dimethyl Fumarate (DMF; Tecfidera), Glatiramer Acetate (GA; Copaxone), Interferon-beta (IFN-β; Rebif, Avonex, Betaseron, Extavia, Plegridy) and Teriflunomide (TF; Aubagio) [17,18,19,20,21,22,23,24,25,26,27,28]. The CNS-directed molecular effects of more recently approved drugs, such as Laquinimod (LQ; Nerventra), Natalizumab (NZ; Tysabri), Alemtuzumab (AZ; Lemtrada) and Orcelizumab (OCR; Ocrevus), have been less well described, except for the neuroprotective effects of LQ and NZ [29,30,31]. In general, each of these previous studies has reported the (molecular) effects of only one or two MS drugs (e.g., [28,29,31]) on one or two CNS cell types (e.g., [22]). Moreover, the protective effects of MS drugs on neurons and oligodendrocytes have often been attributed to indirect effects caused by the actions of MS drugs on peripheral immune cells (e.g., [28]). Therefore, the effects of MS drugs have not been documented in multiple CNS cell types nor integrated into a common molecular cascade of events. The goal of the present review is to describe and compare the molecular effects of the traditional and recent FDA-approved MS drugs on multiple CNS cell types, focusing on microglia within the generally applied homeostatic (M0), pro-inflammatory (M1) and anti-inflammatory (M2) designation [32,33], and on astrocytes within the homeostatic (A0), reactive (A1) and neuroprotective (A2) nomenclature [34], as well as on neurons and oligodendrocytes.

## 2. Molecular Effects of FDA-Approved MS Drugs on CNS Cells

### 2.1. Molecular Effects of FTY720

The synthetic compound FTY720 is a structural analogue of the natural molecule sphingosine that modulates the Sphingosine 1-phosphate receptor (S1PR) in immune and brain cells [17,18,19,35,36,37,38], and is FDA approved as an oral therapeutic for relapsing-remitting MS (RRMS). Phosphorylated FTY720 (pFTY720) is able to agonize the S1PR, which causes the internalization of the receptor and functional antagonism [39]. The molecular effects of FTY720 on peripheral monocyte populations are reviewed elsewhere [12] and include the promotion of anti-inflammatory cytokine profiles of B- and T-cells. Both the prominent S1P expression in neural cells [38], and its widespread effects on the proliferation, differentiation and the migration of neural cells [40] together with the fact that FTY720 is proven to be lipophilic and thus capable of crossing the BBB [41], make this drug an attractive treatment for diseases with neural pathology. The clear effect of FTY720 treatment on remyelination in the CNS has already been reviewed elsewhere [22,23]. In the next sections, we discuss the molecular effects of FTY720 specifically on microglia, astrocytes, neurons and oligodendrocytes (for details, see Supplementary Table S1; for article search terms, see Supplementary Information).

#### 2.1.1. Microglia

Both in vitro and in vivo studies have shown that FTY720 has inhibitory effects on the pro-inflammatory (M1) microglia phenotype and stimulates the anti-inflammatory (M2) microglia phenotype [42,43,44,45,46,47,48,49,50,51,52,53,54,55,56,57,58,59,60,61,62,63,64,65,66,67,68,69,70,71]. In rodent models for several diseases, such as stroke, cuprizone-induced demyelination, MS (experimental autoimmune encephalitis, EAE), familial Alzheimer’s disease (AD) and in irradiation-induced injury, FTY720 decreased microglia activation by polarization towards an anti-inflammatory M2 phenotype characterized by the increased expression of markers such as arginase 1 (ARG1) and mannose receptor C-type 1 (MRC1/CD206) [42,43,44] and a decreased microglia M1 state, defined by the expression of allograft inflammatory factor 1 (IBA1), cluster of differentiation 68 (CD68) and lysosome-associated membrane protein 2 (LAMP-2; CD107b/MAC-3) [42,43,45,46,47,48,49,50,51,52,53,54,55,56,57,58,59,60,61,62,63,64,71] Consistent with these findings, in vitro FTY720 inhibited the expression of M1 microglia markers and promoted the M2 polarization of oxygen–glucose deprivation (OGD)-insulted microglia [55], photothrombotic stroke-derived primary microglia cultures [42] and white matter-derived microglia from an ischemia rat model [43] as well as in the microglia cell line N9 [63], and human and murine primary microglia cultures [65]. Moreover, FTY720 downregulated the mRNA expression of the pro-inflammatory cluster (e.g., C-C motif chemokine ligand (CCL)7, chemokine (C-X-C motif) ligand (CXCL)13 and CCL5) and upregulated genes in the anti-inflammatory cluster (e.g., granulocyte-macrophage colony-stimulating factor (GM-CSF), chitinase 3-like 3 (CHI3L3) and interleukin (IL)-10) in microglia cells from non-obese EAE mice [65]. Interestingly, this shift from M1 to M2 microglia phenotype was found to be modulated via the signal transducer and the activator of transcription (STAT)3 activation in white-matter ischemia [43]. Thus, FTY720 converts a pro-inflammatory environment in the CNS into an anti-inflammatory milieu. 

In lysolecithin-induced demyelination slice cultures, FTY720 enhanced remyelination through increases in IBA1-positive microglia via S1PR1/S1PR5 signaling, which could not be explained by an increased number of phagocytosing microglia [66] and the mechanism underlying this effect on microglia remains unclear. Others have reported no effect of FTY720 on IBA1-positive or MAC-3-positive cell numbers in the cuprizone-demyelination model [67,68,69] and in mice with facial nerve lesions [70]. 

In addition to affecting the phenotype, FTY720 modulated the secretion of pro-inflammatory factors and programmed cell death pathways in microglia [42,43,44,48,54,55,58,65,72,73,74,75]. In general, FTY720 treatment reduced the secretion of pro-inflammatory cytokines such as tumor necrosis factor alpha (TNF-α), IL-1β, IL-6 and CXCL5 in in vitro models such as lipopolysaccharide (LPS)- or 1-methyl-4-phenylpyridinium (MPP)-treated microglia cell lines and primary cultures [43,44,55,58,72,73,74]. A diminished presence of pro-inflammatory cytokines following FTY720 treatment was also found in in vivo rodent models for intracerebral hemorrhage (ICH) [47], cuprizone-induced demyelination [48], status epilepticus (SE) [54] and an 1-methyl-4-phenyl-1,2,3,6-tetrahydropyridine (MPTP)-induced model of Parkinson’s disease (PD) [58]. In line with this, mRNA expression profiling in primary mouse microglia showed that FTY720 suppressed the expression of a wide array of LPS-induced inflammatory molecules such as IL-1α, IL-1β and CCL2 via a decrease in the expression of the transcription factors STAT1 and interferon regulatory factor (IRF)8 [74]. However, FTY720 did not affect the expression of the pro-inflammatory cytokines TNFα, IL-1β and CCL2 in non-activated human and murine microglia cultures [65], and even increased the microglial secretion of the T-cell chemo-attractant IL-16 in a mouse traumatic brain injury (TBI) model [75]. In addition to pro-inflammatory cytokines, FTY720 reduced the expression and secretion of the oxidative stress factors inducible nitric oxide synthase (iNOS) and reactive oxygen species (ROS) in vitro and in vivo [42,55]. Taken together, these results show that FTY720 reduces the pro-inflammatory environment created by activated M1 microglia.

The production and secretion of pro-inflammatory factors is regulated via a number of molecular signaling cascades [76]. In LPS-stimulated mouse primary microglia, FTY720 reduced the signaling of the p38 mitogen-activated protein kinases (MAPK) stress pathway, but did not modulate c-Jun N-terminal kinase (JNK)1/2 activation [59] and reduced phosphoinositide 3-kinase (PI3K), protein kinase B (AKT) and glycogen synthase kinase 3 beta (GSK3β) phosphorylation in MPP-treated microglia [58]. Furthermore, FTY720 slightly reduced Aβ42-induced pro-inflammatory cytokine production in LPS-induced Spinster homolog 2 (SPN2) knock-out and wild-type microglia [44] and inhibited the activation of nuclear factor binding near the kappa-light-chain gene in B cell (NFκB) [44,55,58] and reduced the NLR family pyrin domain containing 3 (NLRP3) and caspase (CASP)1 levels [55,58]. Surprisingly, FTY720 upregulated the expression of IL-1β and cleaved CASP1, and the oligomerization and dimerization of apoptosis-associated speck-like protein (ASC) in wild-type primary microglia, but not in ASC knock-out microglia [77], which implies again that FTY720 targets NFκB. Moreover in ischemia white matter-derived primary microglia cultures, FTY720 increased the levels of phosphorylated (p)STAT3 [43], which closely acts together with NFκB in inflammatory signaling cascades [78,79]. These results suggest that FTY720 targets signaling cascades involved in inflammation, such as NFκB and STAT3.

The additional activities of FTY720 are involved in the survival aspects of both microglia cells and other cell types via their interactions with microglia cells. The non-phosphorylated form of FTY720 stimulated the cleavage of CASP7 and CASP9, and induced apoptosis in the human microglial cell line HMO6 through the Sterol regulatory element-binding protein 2 (SREBP2) pro-apoptotic pathway, independent from S1PR binding [80]. This suggests that FTY720 also targets other pathways alongside S1P. In addition, FTY720 enhanced the expression of the growth factors brain-derived neurotrophic factor (BDNF) and glial cell line-derived neurotrophic factor (GDNF) in primary microglia [72], possibly showing its effect on neural trophic support via microglia. FTY720 treatment also increased the cell survival of neurons in co-culture with microglia on microfluidic chips and exposed to neurotoxic oligomers of amyloid-beta (Aβ) [81]. In support of a less inflammatory CNS environment, FTY720 also reduced the total brain Aβ-levels in a mouse model of AD [45], possibly via microglia cells which play a major role in the internalization and degradation of Aβ [82].

In summary, FTY720 targets the pro-inflammatory M1 phenotype, the downstream production and secretion of pro-inflammatory cytokines, and programmed cell-death pathways in microglia cells.

#### 2.1.2. Astrocytes 

In the astrocytes, but not in the neurons, of EAE mice lacking S1PR1, the neuroprotective effect of pFTY720 was diminished [82], showing its effect is mediated via the astrocytic S1PR1. In addition, cultured rat primary astrocytes represented the major cell type to respond to pFTY720, via the activation of Gi protein-mediated S1PR1 signaling cascades [83]. S1PR modulation is therefore a key target of FTY720 in the CNS and besides microglia, this compound acts on astrocytes. 

FTY720 reduced the number of cells positive for reactive A1 astrocytic markers glial fibrillary acidic protein (GFAP) and S100 calcium-binding protein B (S100β) in multiple rodent models, including in a model of familial AD [45,51], cuprizone-induced demyelination [48], MS [50,61,82], Huntington’s disease (HD) [84], Status epilepticus (SE) [54], and models of infection [85], stroke [86], ICH [47], Pentylenetetrazol (PTZ)-induced kindling [56], and maternal inflammation [87] as well as wild-type rodents [88]. In contrast, FTY720-treated immortalized astrocytes displayed increased release of the astrocyte-secreted protein GM-CSF [89], whereas LPS-stimulated primary astrocytes decreased GM-CSF release following treatment with FTY720 [65]. No effect of FTY720 on astrocyte reactivity status has been found [46,64,67,68,69,70] and even an increased number of GFAP-positive cells has been reported in in vivo models [66,90]. 

In addition to a possible effect on the reactivity of astrocytes in vivo and in vitro, FTY720 modulates the production and secretion of pro- and anti-inflammatory cytokines by astrocytes. In non-obese diabetes EAE mice, FTY720 administration resulted in the decreased expression of reactive A1 astrocytic factors (including manganese-dependent superoxide dismutase (MnSOD), IL-6, CCL2, CXCL10, IL-1β, TNFα), and an upregulation of CXCL12 and IL-33 [65]. This effect of FTY720 on pro-inflammatory cytokines was confirmed in other rodent disease models [47,48,54,84,87,91]. Furthermore, the expression and secretion of pro-inflammatory cytokines in vitro was also reduced following treatment with FTY720 in both human and rodent primary cells from fetal and adult origin [65,73,92,93,94] as well as in an astrocytoma cell line [95], while only one study has reported no effect of FTY720 on IL-1β and CCL2 [96]. The number of immediate-early astrocytes, characterized by FBJ murine osteosarcoma viral oncogene homolog (c-Fos)activation, was reduced following the treatment of the mouse EAE model with FTY720 [97]. It is currently not clear whether these c-Fos-positive astrocytes are neurotoxic (A1) or neuroprotective (A2), or how these cells contribute to MS pathology. Although the results of a minority of the studies are ambiguous, FTY720 generally decreased the reactive A1 astrocytic phenotype and the associated pro-inflammatory cytokine secretion.

A number of studies has reported FTY720-mediated effects on cellular stress and inflammation pathways in astrocytes. In vitro, interferon-gamma (IFN-γ)-stimulated rat astrocytes increased ADR-β2 and reduced MHC-II expression following FTY720- and pFTY720 treatment through a decrease in NFκB p65 (also named RelA) [98]. In addition, FTY720 inhibited the degree of phosphorylation of NFκB and p65 both after cellular stress by OGD and in unstimulated primary astrocyte cultures [65,90,92,93], and increased the inhibitor NFκB-alpha (Iκbα) levels in a mouse HD model [84], both leading to a reduced NFκB activation. The observed effects of FTY720 on pro-inflammatory cytokine production in astrocytes can be explained by these effects on NFκB, and are supported by its inhibitory effect on the production of the NFκB down-stream targets NLRP3 and IL-1β, which was not present in S1PR1 knock-out astrocytes [99]. Cultured astrocytes treated with FTY720 upregulated leukemia inhibitory factor (LIF), heparin binding EGF-like growth factor (HBEGF) and IL-11 expression [95], molecules related to growth factors and MAPK signaling. Furthermore, in vitro and in vivo FTY720 affected the signaling pathways extracellular signal-regulated kinase (ERK)1/2 and MAPK, both known to regulate NFκB activation [100,101]. FTY720-treated cultured astrocytes also decreased expression of the reactive astrocyte protein high mobility group box 1 (HMGB1) and induced a blockade of Toll-like receptor (TLR)2 and PI3K signaling [93].

In addition to the activation, the cytokine secretion and the induction of inflammation pathways of astrocytes, other effects of FTY720 on astrocytes with a high relevance to neurodegenerative disease pathology have been reported. FTY720 reduced the mRNA and protein expression of the NFκB-related downstream immune factors intercellular adhesion molecule 1 (ICAM-1) and vascular cell adhesion protein 1 (VCAM-1) [57,94], and increased the mRNA expression of astrocyte-secreted vascular endothelial growth factor A (VEGF-A) [86], indicating its effect on the BBB. Furthermore, FTY720 reduced astrocyte migration and proliferation [98,102], and increased astrocyte survival after OGD in primary rat astrocyte cultures [93,94]. FTY720-treated primary cultures also displayed an elevated mRNA and protein expression of solute carrier family 1 member (SLC1A)3 and SLC1A4, showing the effect of FTY720 on glutamate metabolism, a process important for astrocyte–neuron communication [50]. The pre-treatment of cultured astrocytes with FTY720 before incubation with TNFα reduced the expression of two neuroinflammation markers, sphingomyelinase and ceramide [103]. The expression of oxidative-stress-related factors such as iNOS, Nitric Oxide (NO) and 3-Nitrotyrosine (3-NT) was also diminished by FTY720 in (LPS-stimulated) cultures [65,92] and in mouse models of MS and HD [84,92], as well as FTY720-targeted cyclic adenosine monophosphate (cAMP) signaling and Ca^2+^-signaling in primary rat astrocyte cultures [83,104]. Thus, in addition to the attenuation of the neurotoxic A1 astrocytic phenotype, FTY720 exerts other positive effects on astrocyte functions.

#### 2.1.3. Neurons

A number of effects of FTY720 on neurons has been described regarding protection against environmental stressors and general neuronal viability. In EAE and models of stroke, ischemia and epilepsy, FTY720 treatment resulted in the increased survival of neurons and axonal projections [42,46,47,49,50,54,56,59,60,65,68,82,102]. In vitro, FTY720 prevented neurodegeneration in Aβ-exposed rat neuronal cultures and microglia–neuron co-cultures on microfluidic chips [81], decreased apoptosis in neuroblastoma SH-SY5Y cells [58] and increased the survival of neuronal cells after glutamate or N-methyl-D-aspartate receptor (NDMA)-induced stress or in unstressed primary rodent neurons [59,92,105]. Therefore, neurodegenerative diseases with excitotoxicity as a central hallmark might benefit from FTY720 treatment. Moreover, FTY720 reduced the loss of tyrosine hydroxylase (TH)-positive dopaminergic neurons in a mouse PD model [58]. Additionally the presence of neurofilament-heavy chain (SMI32)-positive or amyloid precursor protein (APP)-positive axonal spheroids, indicating axonal damage, was profoundly reduced following FTY720 treatment in vivo and in rat primary cultures [49,57,68]. In contrast, neuronal loss was not attenuated by FTY720 after cuprizone-induced demyelination [69], white matter ischemia [43] and in demyelination related to Krabbe’s disease [64]. In a rat model of myelin oligodendrocyte glycoprotein (MOG)-induced optic neuritis (MOG-ON), FTY720 reduced axonal damage, but did not prevent the apoptosis of retinal ganglion cells (RGCs) in the optic nerve [57]. Moreover, there was no effect on the number of non-phosphorylated SMI32- or APP-positive axons in FTY720-treated cuprizone mice [67,69]. Nonetheless, FTY720 attenuated dendritic spine loss and the expression of postsynaptic density protein 95 (PSD95) in a mouse model of HD [84], and reduced mossy fiber sprouting in SE rats [54]. At the structural level, the number and lengths of nodes of Ranvier increased due to the FTY720 treatment of white-matter ischemia mice [43]. Moreover, FTY720 normalized the physiological responses of neurons in EAE [56] and ameliorated neurotoxic increases in intra-neuronal Ca2+- in Ca2+-reporter mice [105]. These results indicate an effect of FTY720 on neuronal survival, and both structural and physiological changes that support neuronal transmission.

In line with these neuronal-positive effects, FTY720-treated rat neural stem cells (NSCs) differentiated into neurons and oligodendrocytes faster than non-FTY720-treated NSCs [106], and mouse primary neurons stimulated with the compound showed positive changes in growth cones and neurite outgrowth [70]. However, the addition of the supernatant of FTY720-treated cultures of primary rat astrocytes had no effect on neurite growth of the rat neuroendocrine pheochromocytoma cell line PC12 [50], which may indicate that FTY720 also acts directly on neurons. Furthermore, FTY720 increased the pool of neurons expressing the mature neuronal marker doublecortin (DCX) in mouse dorsal root ganglion (DRG) cultures [70], in mice with kainic acid-induced neurodegeneration [106] and in X-ray-exposed but not in wild-type mice [46]. Moreover, the expression of the neuronal differentiation marker beta-3-tubulin (βIII-Tubulin) increased following the FTY720 treatment of rat-derived primary neurons [92], but the compound did not affect βIII-Tubulin and DCX expression in irradiated cultured cells [46], and did not promote the differentiation of human-induced pluripotent stem cells into neuronal nuclei (NeuN)-positive cells after transplantation into wild-type mice [88]. These results point to an effect of FTY720 on the proliferation and differentiation of neurons, possibly due to an increased phosphorylation of ERK 1/2, cAMP-response element binding protein (CREB) and p38 MAPK [107]. However, since FTY720 did not alter the number of calbindin-positive neurons in the cerebellum, Purkinje neurons appear not to be affected by FTY720 [64].

The cascade of intracellular events affected by FTY720 may include the increased neuronal expression of immediate early genes such as cFOS, FOSb and early growth response (EGR)1/2 [70]. Moreover, FTY720 directly affects the expression of S1P receptors, G12/13G-proteins, RhoA-GTPases, and the transcription factors serum response factor (SRF) and MKL/megakaryoblastic leukemia 1 (MRTF) in neurons [70,84], all being relevant modulators of neuronal differentiation and synaptic plasticity. Furthermore, specifically in the neuronal soma of the EAE CNS [61], and in the CNS of wild-type mice [70] FTY720 increased mRNA expression of BDNF, a growth factor promoting neuronal cell survival in addition to its functions in the neuro-immune-axis [108].

Thus, FTY720 exerts direct effects on neuronal excitotoxicity and neuronal differentiation, as well as an indirect effect on neuronal viability via other CNS cell types. Nonetheless, inconsistencies exist concerning the effect of FTY720 on axonal preservation, depending on the experimental model used.

#### 2.1.4. Oligodendrocytes

Remyelination can be induced by FTY720 either via a direct effect on oligodendrocyte precursor cells (OPCs)/oligodendrocytes or indirectly by supporting other CNS cell types such as microglia and astrocytes, which provide metabolic and structural support to oligodendrocytes [109]. FTY720 reduced the extent of demyelination in the CNS of inflammation-induced and other demyelination rodent models [50,56,57,60,65,82,88,107,110,111], in line with the increased axonal myelin sheaths formed in FTY720-treated human fetal OPC cultures [107]. However, FTY720 did not promote remyelination or reduce demyelination in the cuprizone-induced demyelination model [67,69], which may be explained by the timing of the FTY720 administration. More specifically, to be effective in reducing demyelination and suppressing oligodendrocyte cell death, FTY720 should be applied within 10 days after initiating the cuprizone diet [48]. The fact that FTY720 caused myelin recovery following acute, but not chronic, cuprizone exposure [68], may also explain why FTY720 was not found to be effective in some cuprizone-diet studies. Overall, we conclude that FTY720 reduces the extent of demyelination, with the timing of disease induction and treatment being important for its efficacy.

Reduced demyelination may be attributed to a reduced degree of apoptosis of OPCs and oligodendrocytes [112,113]. Indeed, FTY720 reduced the oligodendrocyte apoptosis both in vivo [48] and in vitro [43,107,114,115,116]. In line with this, FTY720 increased the number of proliferating (bromodeoxyuridine/5-bromo-2′-deoxyuridin (BrdU)-expressing) OPCs in the brain [88,106,117], and the expression of the oligodendrocyte lineage marker oligodendrocyte transcription factor (OLIG2) in neural precursors derived from embryonic stem cells (ESCs) [115] and in the brains of Krabbe’s disease mice [64], pointing towards an increased survival of oligodendrocytes. Additionally, in EAE rats and in a human oligodendroglioma cell line, FTY720 reduced ceramide levels [118], which are involved in oligodendrocyte metabolism and apoptosis.

The enhanced differentiation of OPCs towards myelin-producing cells may increase the remyelination of the CNS. In EAE mice, FTY720 increased the expression levels of the OPC proliferation and differentiation factors sonic hedgehog (SHH), smoothened (SMO) and SMO effector GLI family zinc finger 1 (GLI1) [117]. FTY720 treatment also promoted OPC differentiation in cultured human and rodent oligodendrocytes [43,110,114,116,119] and in the CNS of a number of rodent demyelination models [48,56,64,66,107,110,111,117], as shown by the increased mRNA and protein expression of the mature myelin markers myelin basic protein (MBP), proteolipid protein (PLP1) and 2′,3′-cyclic-nucleotide 3′-phosphodiesterase (CNPase). In contrast, FTY720 did not affect the protein expression of mature myelin markers in the cuprizone-induced demyelination model [67,69]. The reported FTY720-induced increase in OPC differentiation possibly resulted from phosphorylation events in the PI3K/AKT or ERK1/2 pathways [107,110,114,116], albeit one study found that ERK 1/2 was phosphorylated in astrocytes, but not in oligodendrocytes, following treatment with FTY720 [120]. The downregulation of the growth factor BDNF in α-synuclein (αSYN)-treated OLN-93 oligodendroglial cells was counteracted by FTY720 [121], possibly pointing towards a critical role for this sphingosine analogue in oligodendrocyte–neuron communication. Finally, in a demyelination model of Krabbe’s disease, the levels of myelin debris were not diminished by FTY720, while demyelination was reduced [64], suggesting that this compound targets the differentiation rather than apoptosis of OPCs in this disease model. 

Taken together, treatment with FTY720 results in reduced demyelination alongside increased OPC survival and the promotion of OPC differentiation towards myelin-producing cells.

### 2.2. Molecular Effects of DMF

DMF belongs to the group of fumaric acid esters, and represents an FDA-approved treatment for MS and psoriasis [21]. Both DMF and its active metabolite monomethyl fumarate (MMF) exert anti-inflammatory and immune-modulatory effects in the peripheral system, as reviewed elsewhere [21]. DMF and MMF have both been described as activators of the transcription factor nuclear factor erythroid 2-related factor 2 (NRF2) in various neurodegenerative diseases, such as PD, MS and AD [24,25], and DMF also affects other oxidative stress-related pathways, including NFκB and hypoxia-induced factor 1α (HIF-1α) [21]. Furthermore, MMF has a high affinity for the hydroxycarboxylic acid receptor 2 (HCAR2), which is not only expressed in monocyte-derived macrophages, but also in microglia and astrocytes [122]. In this section, we will discuss the specific molecular effects of DMF and MMF on CNS cell types (for details, see Supplementary Table S2; for article search terms, see Supplementary Information).

#### 2.2.1. Microglia

Microglial M1 pro-inflammatory status, as measured by the number of IBA1- and CD68-positive cells, was reduced following DMF treatment in hypoperfused mice [123], in OGD-stressed rats [124], LPS-treated mice, vertebral hypoxic ischemia in mice [125], EAE mice [126], the mouse MPTP model of PD [127], rumpshaker hypomyelination mice [128], after spinal cord injury (SCI) in mice [129], ICH in mice [130] and in wild-type mice [131] as well as in mouse primary microglia cultures [132]. Interestingly, DMF increased the number of cluster of differentiation 86 (CD86)-positive microglia in aged rats with streptozotocin-induced AD [133], possibly due to altered inflammatory signaling in aged compared to non-aged microglia [7]. Levels of M2 microglia phenotype markers, such as MRC1/CD206, resistin-like alpha (RETNLA) and ARG1, were elevated by the addition of DMF to (activated) primary microglia cultures [134,135]. Similarly, treatment with the DMF-metabolite MMF decreased pro-inflammatory M1 marker expression [136] and increased anti-inflammatory M2 marker expression in vivo and in vitro [124,137], although one study did not confirm these findings [135]. No effect of MMF on the inflammatory state of microglia cells was found in cuprizone mice [138], and in human fetal and adult microglia cultures stimulated with LPS [122].

Moreover, DMF did not diminish the number of pro-inflammatory CXCR3-positive microglia cells in hippocampal slices stimulated with ATP [134]. In EAE animals, DMF alone did not show any significant effect on microglia, but interestingly, a combined treatment of DMF and IFN-β resulted in a reduction of the number of inflammation-linked MAC-3-positive microglia [139]. Furthermore, microglial phagocytic activity increased following the DMF treatment of primary rat cultures [140] and MMF treatment of the cell line N9 [137], but was decreased in LPS-stimulated mouse microglia cultures [134]. Altogether, DMF and also MMF may induce a switch from a pro-inflammatory M1 to an anti-inflammatory M2 phenotype in a wide variety of CNS-related diseases, but it is not clear whether the resulting microglia become more phagocytic.

In accordance with a less-activated M1 microglia phenotype, the expression and secretion of pro-inflammatory mediators were reduced not only in DMF-treated primary rodent cultures [122,124,134,141,142,143] and BV-2 microglia cells [144,145], but also in DMF-treated OGD-stressed rats [124]. However, in mouse cortical explants, DMF had no effect on the expression of IL-1β [145]. The effect of MMF on pro-inflammatory mediators was ambiguous, since in LPS- and IFN-γ-stimulated primary human and rodent cultures, MMF did not affect IL-6 and TNFα expression [135,143,146], while expression levels of these factors were decreased in MMF-treated N9 cells [137] and isolated microglia cultures from glioblastoma multiforme tumors [136]. In MMF-treated co-cultures of HMC3 microglia cells and monocytes, the expression of CXCL10 by microglia was decreased [127]. Although not unequivocally demonstrated but at least in human and rodent microglia cells, DMF and MMF appear to reduce the pro-inflammatory microglia phenotype alongside a reduction in the secretion of pro-inflammatory mediators. This notion is also supported by a clear reduction in pro-inflammatory cytokine expression in the total brain tissue following the DMF treatment of rodents exposed to stroke, LPS-stimulation, spinal cord injury and MPTP-induction [125,127,129,132,140].

The effect of DMF on cytokine expression may be evoked by its inhibitory effect on NFκB p65 levels [132] and NFκB target-gene expression [143]. DMF indeed induced total brain expression of the NFκB inhibitor IκBα and reduced the NFκB expression levels in the brain tissues of MPTP PD mice [127] and in a mouse model of SCI [129]. Moreover, in rat primary microglia, the DMF treatment resulted in decreased levels of pERK [142], an upstream regulator of NFκB. The microRNA 155 (miR-155) expression, which is transcriptionally regulated by NFκB, was also decreased by DMF in fetal and adult human microglia cultures [122]. The divergent effects of DMF and MMF described above may be explained by the fact that, in contrast to DMF, MMF causes not only an upregulation of NFκB target genes [143], but it also decreases NFκB p65 acetylation in microglia cells, resulting in a more inflammatory environment [137].

In microglia, DMF treatment may also affect antioxidant signaling, as the expression of oxidative stress factors such as NO and iNOS was diminished in DMF-treated rodent primary microglia [124,132,134,141,142,143], and the microglia cell lines BV-2 and N9 [137,145]. Furthermore, the expression of proteins important for the protection of cells against oxidative stress, such as Heme oxygenase 1 (HO-1), NAD(P)H dehydrogenase quinone (NQO-1) and glutathione (GSH), was elevated following the DMF treatment [122,124,141,144,145]. The results of these studies are supported by the attenuation of the oxidative milieu found in total brain tissue following the DMF treatment of wild-type mice [131], mice with ICH [140] and mouse models of cerebral hypoxic ischemia [125], PD [127] and SCI [129]. Moreover, DMF showed an inhibitory effect on LPS-stimulated NO bursts in cultured mouse microglia cells [138]. These results point to a role of DMF in the attenuation of oxidative stress in microglia cells. In addition, the DMF treatment of microglia in the culture resulted in decreased motility, a decreased response to ATP and increased ferritin uptake [134]. Moreover, supernatants from cultured DMF- and MMF-treated microglia cells enhanced OPC proliferation in vitro [135] and DMF reduced microglia toxicity towards neurons [132]. These findings indicate that the effects of DMF on microglia have also profound downstream influence on other CNS cell types.

#### 2.2.2. Astrocytes

Multiple in vivo studies have reported that the application of DMF leads to a reduction in the number of reactive A1 astrocytes, based on decreases in the number of cells positive for the reactive astrocytic marker GFAP in wild-type mice [131], LPS-stressed C-X-C motif chemokine receptor (CXCR)1-GFP- labeled mice [132] and a mouse model of SCI [129]. In the mouse cerebral hypoxic ischemia model, DMF diminished GFAP-positive cells only after 6 h of treatment, but the number of reactive astrocytes increased after 24 h [125]. However, DMF had no effect on the reduced number of reactive A1 astrocytes in hypomyelinated rumpshaker mice [128] and the fumaric ester did not induce changes in TNFα, IL-6 and IL-1β expression in LPS- or IFN-y- and IL-1β-stimulated rat primary astrocytes [147]. Nevertheless, DMF as well as its metabolite MMF, decreased the secretion of pro-inflammatory cytokines such as TNFα, IL-1β, CXCL10 and IL-6 from cultured LPS- or IL-1β-stimulated rodent and human primary astrocytes [142,148], and rat mixed glia and neuron cultures [124], respectively. Taken together, DMF and MMF affected the expression of pro-inflammatory cytokines in astrocytes and might have reduced A1 astrocyte numbers in a time-dependent manner.

The anti-oxidative capacity of DMF became apparent in a study on the co-cultures of microglia and astrocytes, in which the compound upregulated expression of the anti-oxidant factors NQO-1 and HO-1 [149]. Furthermore, DMF and MMF reduced intracellular ROS production, but had no effect on the mRNA expression of antioxidant-related genes in IL-1β-stimulated human and mouse microglia cultures [148]. Interestingly, the 24 h treatment of rat primary astrocytes with DMF-reduced histone deacetylase (HDAC)1, HDAC2 and HDAC4 protein levels in the presence of cytokines, and the resulting nuclear NRF2:DNA binding activity was blocked by a selective inhibitor of the NRF2-target gene HO-1 [150], which again suggests a role for DMF in the modulation of oxidative stress. 

A number of additional effects of DMF on astrocytes has been reported, e.g., in astrocytes from the mouse cerebral hypoxic ischemia model, and DMF reduced the levels of glutamine synthetase (GS) which is important for glutamine metabolism [125]. Moreover, following LPS stimulation or cytokine shock in primary rat astrocytes, the secretion of the growth factors nerve growth factor beta (NGF), GDNF, ciliary neurotrophic factor (CNTF), BDNF and fibroblast growth factor 2 (FGF2) was not modulated by DMF treatment [147], whereas GDNF, BDNF and neurotrophin-3 (NT3) expression was increased by DMF in astrocytes from SCI mice [129]. Other effects of DMF are the increased expression of the insulin-like growth factor (IGF-1) regulator placental alkaline phosphatase (PLAP) in the astrocytic cell line N18-RE-105 [151], and the decreased expression of miR-155 in human and mouse primary astrocyte cultures [147]. Finally, combined DMF–MMF treatment reduced the viability of glioblastoma tumor cells, which to some extent resembled the cells of the astrocytic lineage [136]. Thus, in addition to the downregulation of the pro-inflammatory environment and decrease in oxidative stress, DMF induced growth factors and metabolites important for astrocyte–neuron communication. The limited number of studies on the effect of MMF on oxidative stress precludes a firm conclusion.

#### 2.2.3. Neurons

DMF decreased neuronal cell death in wild-type [131] and EAE mice [139], as well as in rodent models of AD [133], ICH [130] and cerebral hypoxic ischemia [125]. In addition, DMF reduced the neurodegeneration and the number of α-Syn-positive neurons in an MPTP mouse model of PD [127]. The effect of DMF on neurodegeneration in the mouse cuprizone model remains unclear, as both a reduction [126] and no effect on the number of APP-positive axons [138] have been reported in this model. Moreover, in a mouse model of hypoperfusion, DMF did not diminish the number of APP-positive axonal spheroids [123], whereas in DMF-treated EAE mice, the number of preserved axons in inflamed lesions was doubled [139]. Treatment with the DMF-metabolite MMF reduced the toxic effect of HIV particle-stimulated microglia–monocyte co-cultures towards human fetal neurons [152]. Furthermore, MMF rescued cultured cortical neurons from OGD [124], and MMF and DMF increased the cell survival of primary human and mouse neuronal cultures stimulated with microglia medium [132,146], alongside a reduction in cleaved CASP3-positive apoptotic neuronal cells [132]. Treatment with DMF also protected primary mouse cortical neurons against glutamate- and NMDA-induced neurotoxicity [105], and neuroblastoma SH-SY5Y cells against MPTP-stress [127]. Finally, decreased neuronal damage was found in a brain biopsy of a DMF-treated MS patient when compared to the damage in treatment-naïve patient material [126]. Ex vivo monitoring of glutamate-mediated excitatory postsynaptic currents in mouse EAE-slice cultures showed that the effect of DMF on neurotoxicity was exerted via the modulation of glutamate release [137]. Taken together, these results confirm that both DMF and MMF play a role in inhibiting neurodegeneration, and as suggested by the results of three in vitro studies [132,146,152], DMF may achieve this through reducing microglial toxicity.

DMF also increased the levels of the antioxidants MnSOD-, HO-1- and GSH in MPTP-treated SH-SY5Y cells, and reduced the expression of cyclooxygenase 2 (COX2) and nNOS in a mouse model of PD [127]. In line with these results, HO-1 expression was increased in DMF-treated fetal microglia cultures stimulated with HIV-macrophage medium [152] and the compound decreased the expression levels of the oxidative stress marker 3-NT in a rat model of AD [133].

In addition, DMF may have an effect on dopaminergic neurons because it increased the number of TH-positive neurons as well as TH- and dopamine transporter (DAT) levels in a mouse PD model [127]. In line with these pro-neuronal findings, DMF diminished the phosphorylated Tau (pTau) protein levels in vivo [131] and reduced the mitochondrial respiratory deficits [143] and membrane potential disruption [132] in microglia-medium-treated rodent neuronal cultures. Moreover, the levels of the neuronal differentiation markers neurofilament (NF) and growth associated protein 43 (GAP-43) increased following DMF treatment in a mouse sciatic nerve model [153]. Finally, even though DMF did not affect axonal refractoriness in hypoperfused mice, the evoked compound action potentials (CAPs) improved after treatment [123] and DMF reduced the intracellular Ca^2+^-levels in transgenic mice carrying the calcium-sensing fluorescent protein TN-XXL [105], again supporting a protective role of DMF towards neurons.

#### 2.2.4. Oligodendrocytes

DMF treatment of EAE mice increased Luxol fast blue (LFB) myelin-staining levels compared to non-DMF-treated animals [126,154] in a HCAR2-dependent manner [154]. Moreover, in the corpus callosum of a non-inflammatory, but toxin-induced mouse demyelination model, LFB myelin signals were raised by DMF treatment [138] and by the administration of DMF-containing lipoidal nanoparticles [155]. Interestingly, combined DMF- and IFN-β treatment, but not DMF treatment alone, rescued myelination following EAE induction [139]. Thus, in rodent models of both MS and demyelination induced by a toxin, DMF affects myelination.

Furthermore, a higher number of mature oligodendrocytes was present in a brain-biopsy of a DMF-treated MS patient than in the treatment-naïve biopsies [126] and supernatants from DMF- as well as MMF-treated microglia cultures enhanced the proliferation of OPCs in vitro [135]. However, DMF did not increase the expression of myelin- and oligodendrocyte-related proteins and lipids nor myelin thickness (g-ratio) in hypoperfused mice [123], primary rat oligodendrocyte cultures treated with microglia medium [135] and the mouse rumpshaker hypomyelination model [128]. Nonetheless, DMF elevated the expression of the myelin-related proteins MBP, PLP, myelin-associated glycoprotein (MAG) and myelin oligodendrocyte glycoprotein (MOG) in cuprizone-fed mice [138] as well as the number of NPCs positive for the oligodendrocyte marker O4 [148]. The survival of the oligodendrocyte progenitor cell line CG4 was not increased by DMF treatment following stimulation with hydrogen peroxide (H_2_O_2_) or a NO donor [138]. Thus, it is unclear whether DMF has a direct effect on oligodendrocyte lineage progression and overall oligodendrocyte survival. Nevertheless, the exposure of the oligodendrocyte cell line MO3.13 to DMF did result in changes in the levels of lipids, GSH and the intermediates of the citric acid cycle [156], indicating that to some extent the fumarate ester does affect oligodendrocyte metabolism.

### 2.3. Molecular Effects of GA

GA (also known as copolymer-1 or copaxone) is a mixture of synthetic polypeptides containing four naturally occurring amino acids (L-glutamic acid, L-alanine, L-tyrosine and L-lysine), approved for the treatment of RRMS and mainly known for its effect on T-cell differentiation [157]. Moreover, GA inhibits the activation of MBP-reactive T-cells and creates an anti-inflammatory T-cell environment by preventing the entry of these cells into the brain [158]. In addition, GA may only minimally protect the BBB [159], and exert an effect beyond the immunomodulation of cells of the innate and adaptive immune systems. For instance, GA protects neurons and oligodendrocytes and affects three processes characteristic of neurogenesis (neuronal progenitor cell proliferation, migration, and differentiation), as reviewed elsewhere [26,27,28]. Moreover, a limited number of studies has reported that GA might affect general AKT and MAPK signaling in the brain [160,161]. In addition, the neuroprotective effect of GA is still considered to be a side-effect of the suppression of immune cell populations [28]. The following sections focus on the direct molecular effects of GA on cells of the CNS (for details, see Supplementary Table S3; for article search terms, see Supplementary Information).

#### 2.3.1. Microglia

Most studies report a reduction in the number of IBA1-positive and protein tyrosine-protein phosphatase (CD45)-positive activated microglia following the treatment of a rat cranial irradiation model with GA [162], the mouse EAE model [160,161] and neuropathic allodynia rats [163], which points to a decrease in the number of pro-inflammatory M1 microglia. Moreover, the density of cells positive for the pro-inflammatory microglia markers integrin alpha-M precursor (MAC-1/ITGAM) and galectin-3 (MAC-2) was decreased in PLP- and MOG-induced EAE mice [164,165,166] and in a double-transgenic (APP/Presenilin-1; PS1) mouse AD model [167]. In addition, the morphological transformation of ramified resting microglia into a pro-inflammatory amoeboid morphology was attenuated in vitro by GA in human primary fetal and adult microglia activated by T-lymphocytes [168] and in EAE-mouse slice cultures [169]. In contrast, the number of IBA1-positive microglia increased in the hippocampus of a rat model of cranial irradiation-induced brain injury [162] and GA-treated cuprizone mice showed an expansion of MAC-1 and CD68-positive M1 microglia numbers [170]. Moreover, in primary cultures, the number of MAC-1-positive microglia was increased following the GA treatment [170]. Furthermore, GA treatment induced a phagocytic phenotype in cultured rat microglia cells [171] and increased the bacterial phagocytosis of IFN-γ-stimulated primary microglia [172], but not the phagocytosis of autologous human peripheral blood-derived mononuclear cells [173]. Moreover, GA caused a switch of microglia to an Integrin alpha-X precursor (CD11c/ITGAX)-positive dendritic-like phenotype in AD mice [167]. The exact effect of GA on the microglia M1 phenotype and its consequences may therefore depend on whether the model used is characterized by inflammation-dependent or inflammation-independent CNS pathology. Still, the previous studies suggest that GA might attenuate the M1 microglia phenotype and induce a switch towards phagocytosis rather than inflammation.

More consistent evidence towards a shift from a pro-inflammatory to an anti-inflammatory phenotype has been provided through studies analyzing the cytokines secreted by M1 pro-inflammatory microglia following treatment with GA. For example, a decrease in TNFα secretion was found after the GA treatment of BV-2 microglia cells [169], in IBA1-positive cells in EAE-mouse slice cultures [169], in primary rat microglia cultures [171], and in human fetal and adult microglia activated by T-lymphocytes [168]. In line with this, the migration of GA-exposed T-cells into the brains of EAE mice resulted in the increased expression of the anti-inflammatory microglial markers IL-10 and transforming growth factor-beta 2 (TGF-β2) [174], but the GA-treated co-cultures of microglia and activated T-lymphocytes showed a reduction of IL-10 [168]. Furthermore, cultured GA-treated microglia showed increased IL-10 and IL-4 secretion [170,171], again supporting a possible switch from M1 to M2 microglia following GA treatment. Another contributor to a diminished pro-inflammatory environment is the reduction in the levels of the IL-17 protein, a strong inducer of inflammation, in GA-treated EAE mice [164]. In addition, TNFα and IL-6 levels are decreased in the total brain tissue of GA-treated irradiated rats [162] and EAE mice [175]. 

Thus, in addition to a change in microglia phenotype, GA shows effects on the expression and secretion of downstream inflammatory factors.

#### 2.3.2. Astrocytes

The number of GFAP-positive cells in the CNS of EAE mice was reduced following GA treatment [176], although the polypeptide mixture did not affect GFAP-positive cell numbers in cranially irradiated rats [162]. Furthermore, infiltration of GA-induced Th2/3 cells into the brains of EAE mice caused increased expression of the anti-inflammatory factors IL-10 and TGF-β2 by astrocytes [174]. In addition, the bioactive BDNF expression was upregulated in astrocytes in GA-treated mouse HD models [177] and in primary mouse mesencephalic astrocyte cultures [177], which is in line with increased BDNF expression in CNS tissue of the rodent EAE, HD and irradiation models following GA treatment [160,161,162,178]. These results may point towards a GA-induced switch of reactive A1 astrocytes towards a neuroprotective A2 phenotype.

#### 2.3.3. Neurons

GA reduced the number of APP-positive axons [160,179,180] and SMI-32-positive cells [180,181] and thus axonal damage in the CNS of EAE mice. Moreover, in a mouse model of organophosphate intoxication rescue of retinal neurons from cell death was reached by vaccination with GA [182] and neurodegeneration in mouse HD models was attenuated following GA treatment [177]. GA was even able to completely eliminate EAE pathology when the treatment was started at the same time as the induction of EAE in mice [183], which is in line with other studies, showing that GA administered at various stages of EAE induction led to a reduction of neuronal pathology and an increase in the number of BrdU/DCX-positive neurons [166]. In a cranial-irradiated rat model and a mouse AD model, GA also increased the number of BrdU/DCX-positive and BrdU/NeuN-positive neurons [162,176], confirming its positive effect on neuronal differentiation. Specifically, in the frontal cortex and hippocampus of EAE mice, the number of necroptotic receptor-interacting protein kinase 3 (RIP3)/NeuN-positive neurons decreased following the GA treatment [176]. In addition, 1H-magnetic resonance spectroscopy (MRS) of the MS brain showed that the N-acetyl aspartate (NAA)/creatine (Cr) ratio improved following GA treatment [184], indicating decreased neuronal damage, whereas no effect of such treatment on glutamate, NAA, Cr and phosphoCr (pCr) was found in the lesioned white matter of MS patients [185]. Still, GA increased the number of SMI32-positive neurons in EAE mice and the protein levels of the apoptosis marker cleaved CASP3 were reduced in primary mouse motor neurons treated with GA-astrocyte medium [177]. These results suggest that GA increases the survival of neurons and may display neuroprotective effects, possibly via its effect on astrocytes.

The therapeutic effect of GA on SMI32-positive neuronal cells was partially reversed after EAE induction in BDNF-knockout mice [181], suggesting that the neurotrophic factor was a crucial GA-target. This was confirmed since GA treatment increased BDNF expression in the DCX-positive cells of the mouse EAE model [166], as well as the total white matter of mouse lysolecithin-induced demyelination [186], cranially irradiated rats [162], EAE rodents [160,161] and transgenic mouse HD models [178]. However, in chronic EAE, no effect of GA on the total brain BDNF expression was found [187]. 

At the structural level, GA affected axonal conductivity, nodal organization [179] and axonal diameter [179,187,188] in EAE mice. Furthermore, GA treatment reversed glutamate-mediated excitatory postsynaptic currents (EPSC) alterations [169] and the callosal action potential [179] in these mice. In line with this, the mRNA expression of synapse related-genes was induced in EAE mice [175]. The migration of neuronal cells to EAE lesion sites was also augmented by GA [166]. Finally, shortly after the EAE onset, the expression of neuronal-related genes was dysregulated in the spinal cord and normalized by GA treatment [175]. 

In sum, GA might have an overall effect on neuronal fitness via its effect on BDNF expression, and attenuate both structural and physiological axonal features.

#### 2.3.4. Oligodendrocytes

Functional improvements in a number of CNS disease- and injury-based rodent models have been attributed to the reduced demyelination observed following treatment with GA, e.g., in EAE mice, GA increased the number of myelinated (MBP/NF-positive) axons as well as the thickness of the myelin sheath and the resulting g-ratio [188]. Furthermore, the mRNA expression of the oligodendrocyte genes MBP and OLIG2 dysregulated in the EAE spinal cords was normalized by GA [175]. Moreover, both in inflammatory and in non-inflammatory rodent demyelination models, the GA treatment resulted in reduced levels of demyelination [160,161,165,180,186]. However, when GA was applied to the mouse cuprizone model before inducing demyelination, the resulting myelin was still disorganized, whereas the administration during the demyelination phase generated better organized myelin and more mature oligodendrocytes [170]. In line with these findings, GA application during the MOG-immunization of the EAE model resulted in no brain pathology, and the treatment immediately after or one month after MOG-immunization caused less demyelination as well [183]. Thus, GA influences the extent to which demyelination occurs after various forms of injury induction, whereby the effect depends on the timing of GA treatment.

The GA-induced elevated number of myelinated axons may be explained by the fact that the immunomodulator increased the proliferation of OPCs in vivo [170,179,183,188] as well as in embryonic forebrain and primary oligodendrocyte cultures stimulated with a medium from GA-treated T-cells [186,189]. Moreover, a conditioned medium from GA-treated microglia cells induced OPC differentiation [170]. Consistent with this, GA-stimulated oligodendrocyte differentiation was observed in EAE mice [176,183,188], following GA treatment prior to or concomitant with cuprizone demyelination [170], and in GA-treated human T-cells co-cultured with primary human oligodendrocyte cultures [189]. Furthermore, GA treatment increased IGF-1 and BDNF protein levels in both the brain and spinal cord of EAE mice [188]. 

Taken together, GA enhances the proliferation and differentiation of OPCs both in vivo and in vitro, and furthermore may induce growth factor secretion that positively affects multiple CNS cell types.

### 2.4. Molecular Effects of IFNs

Interferons (IFNs) are naturally occurring cytokines, and their effects on the periphery as well as on the CNS are complex and not completely understood [190,191]. IFNs are known to have major effects on microglia cells, which may cause a cascade of consequences in other CNS cell types [192]. Remarkably, endogenous and exogenously administered IFNs may exert anti-inflammatory [193] as well as pro-inflammatory effects [194,195,196,197] in models for neurodegenerative and non-neurodegenerative diseases. The recombinant form of IFN-β has been used as an FDA-approved treatment of MS. IFN-β is a ligand for the Type 1 IFN receptor (IFNAR) [198], which prevents immune-cell infiltration of the brain by its stabilizing effect on the BBB, induces apoptosis of CD4+ and CD8+ Th17-cells, and stimulates the anti-inflammatory phenotype in B-cell populations in the blood [199]. The neuroprotective effects of IFN-β on the CNS are thought to be indirect and all mediated via the attenuation of immune cell functions or blocking BBB leakage [28]. The neuroprotective effects of the not-FDA-approved interferon-alpha (IFN-α) have not been previously reviewed. Since IFN-β and IFN-α have different affinities for binding to the IFNAR1 and IFNAR2 receptors, with IFN-α being the low-affinity cytokine [200], and because the ratio of IFNAR1 to IFNAR2 surface expression determines the downstream signaling events in a given cell [201], the two interferons may differentially affect the CNS. We now describe the effects of a number of recombinant forms of IFN-β on microglia, astrocytes, neurons and oligodendrocytes, namely that of the recombinant human (rh) IFN-β, produced in a mammalian cell expression system (IFN-β-1a, also named Rebif or Avonex), rhIFN-β, produced in a bacterial expression system (IFN-β-1b, Betaseron or Extavia) and IFN-β-1a, linked to polyethylene glycol (pegIFN-β-1a or Plegridy), as well as the effects of IFN-α on CNS cell types (for details, see Supplementary Table S4; for article search terms, see Supplementary Information).

#### 2.4.1. Microglia

The most-described effect of IFN-β on microglia concerns its attenuation of the pro-inflammatory and activated M1 phenotype of these cells. For instance, IFN-β-1 reduced the overproduction of the pro-inflammatory factor and B-cell chemo-attractant CXCL13 in cultured microglia cells from IRF7-deficient mice [202]. In LPS-stimulated microglia cultures, the expression of the neuroinflammation and BBB adhesion factors, matrix metalloproteinase (MMP)-9 and MMP-2 [203] was reduced by IFN-β and IFN-α [204]. In a mouse neovascular age-related macular degeneration (AMD) model [205], in germinal matrix hemorrhage (GMH) rats [206] and in a mouse model of MOG-induced optic neuritis [207], IFN-β and IFN-α both attenuated the levels of IBA1- or CD68-positive M1 microglia, which indicates that in vivo IFNs have positive effects on microgliosis. In line with a reduction in microgliosis, IFN-β-1a reduced the total brain levels of the gliotic inflammation marker myo-inositol in RRMS patients, albeit to a lesser extent than Ocrelizumab (OCR), another MS drug described below [208]. 

In contrast, the intracerebroventricular injection of rIFN-β increased the pro-inflammatory state of microglia cells and complement-dependent synapse elimination in the CNS of a mouse AD model, while blocking the type-I IFN receptor IFNAR1 diminished these effects [209]. The number of IBA1-, CD45- and CD68-positive microglia was also increased following the IFN-β treatment of GBM8-fluc-implanted athymic nude mice [210] and in the offspring of IFN-β-treated mice [211]. In line with these results, the microglial surface expression of the inflammation-associated Fc receptor and major histocompatibility complex class II (MHC-II) proteins was increased following the in vitro administration of IFN-β [212]. Moreover, IFN-β increased both the activated morphology and the number of cells positive for M1 microglia markers in primary mouse microglia [213,214]. In addition to a more pro-inflammatory M1 phenotype, IFNs induced the expression and secretion of pro-inflammatory factors. The levels of CCL5 and CCL2, two major chemo-attractants of peripheral immune cells, were increased in MG6-1 microglia cells, cultured in the presence of IFN-β and TNFα [215], and in IFN-β-treated primary mouse microglia cultures [202]. Moreover, IFN-β and IFN-α enhanced the expression levels of TNF, IL-6, IL-1β, IL-1α, CXCL10, CXCL9 and NO in (LPS-stimulated) primary rodent microglia cultures [209,212,213,216,217,218] and microglia isolated after the maternal separation of the offspring of immune-activated dams [211]. Furthermore, the two interferons downregulated the levels of superoxide anions and glutamate [216], both related to neurotoxicity, and the IFN-β treatment of the primary cultures of microglia stimulated with DNA or RNA upregulated the expression of the members of the Pyrin and HIN200 domain-containing proteins (PYHIN) family of DNA sensors [219], all again pointing to an IFN-mediated switch towards a more pro-inflammatory microglia milieu.

The induction of a pro-inflammatory M1 phenotype by IFNs may be explained by their effect on pro-apoptotic and pro-inflammatory signaling cascades. Nuclear STAT1 levels increased in IFN-β-treated AD mice [209] and the levels of pSTAT1 in microglia were induced by IFN-β in the cell line MG6-1 [215], in mixed mouse glial cultures [209] and in primary mouse microglial cultures [213]. This is in line with the increased STAT1 levels in total brain tissue from the NADH dehydrogenase, subunit 4 (ND4) transgenic mouse demyelination model following the treatment with IFN-β only, or IFN-β combined with vitamin B12 [220]. Moreover in the total brain tissue from human rIFN-α-treated GMH rats, the levels of pSTAT1 and phosphorylated Janus kinase 1 (pJAK1) increased, although the pNFκB-levels decreased [206]. Moreover, the mRNA expression levels of both STAT1 and STAT2 were induced in IFN-α-treated primary mouse microglia cultures [217]. The phosphorylation levels of JAK1 and leukocyte receptor tyrosine kinase (TYK1), factors involved in the phosphorylation of STAT1, were elevated by the combined treatment with IL-1β and TNFα of MG6-1 cells [215]. In primary mouse microglia cultures, IFN-β increased pNFκB-levels in IBA1-positive cells [213]. Furthermore, the expression levels of the NFκB interactors absent in melanoma 2 (AIM2), cyclic GMP–AMP synthase (cGAS) and interferon-inducible protein 204 (p204) were increased by IFN-β in vitro [219]. Thus, IFN treatment results in the activation of pro-inflammatory microglia response pathways involving NFκB and STAT1/2.

In cultured microglia, more than in cultured astrocytes, IFN-α upregulated a wide set of genes related to pathogen detection and elimination, as well as a pro-inflammatory M1 phenotype [217,218]. Furthermore, the systemic administration of IFN-β to dams of a mouse maternal immune activation model increased the stress sensitivity of fetal microglia [211], showing its profound effect on this cell type. Interestingly, neuronal and astrocyte IFNAR signaling, as well as the resulting secreted factors have been found to be important for the activation and proliferation of microglia [221].

Summarizing, while treatment with IFN-β certainly affects microglia cells, its consequences are inconsistent and specific conditions, such as the type of model used, may influence its outcome.

#### 2.4.2. Astrocytes

Like in microglia, a number of studies has shown that the administration of IFN-β or IFN-α caused bimodal effects on astrocytes. In RRMS patients, IFN-β reduced the levels of the gliotic inflammation marker myo-inositol, which is expressed by astrocytes and microglia [208]. Following the IFN-β treatment, a reduced number of reactive A1 astrocytes was also found in the mouse EAE model [220,222], and these effects may be mediated by aryl hydrocarbon receptor (AhR) and suppressor of cytokine signaling 2 (SOCS2) signaling [222]. The increased proliferation of astrocytes in vitro induced by growth factors or cytokines was counteracted by IFN-β treatment [223], indicating that this cytokine may reduce the astrogliosis response. Nonetheless, the administration of IFN-β did not affect cell-cycle arrest in multiple glioma cell lines [224], but did reduce the pathogenic NO production in the astrocytoma cell line A172 [225]. Astrocytes are also important modulators of the endothelial cells that form the BBB. The fact that the application of IFN-β reduced the expression of the BBB-adhesion molecule activators MMP-2 and MMP-9 in LPS-stimulated primary astrocytes in culture [204,226], as well as the permeability of endothelial–astrocyte co-cultures for polysaccharides, suggests that IFNs stabilize the BBB [227,228]. Moreover, IFN-β inhibited the proteolytic MBP-cleavage activity of LPS-stimulated rat astrocytes [226], suggesting that the phagocytic activity of astrocytes may be modulated by IFNs. Importantly, in vitro the negative effects of LPS on the astrocyte proteome were reversed by IFN-β, mainly regarding cytoskeletal proteins and protein degradation, while the expression of protective enzymes such as MnSOD was increased [229]. Following IFN-α treatment, mouse primary astrocytes displayed an expression profile enriched for mRNAs encoding pathogen detection and elimination factors, which again may indicate a more reactive A1 phenotype, whereby the number of differentially expressed genes was lower than that found in microglia cells [217]. These studies suggest that IFN-β reduces, whereas IFN-α may increase the neurotoxic A1 astrocytic phenotype.

In sharp contrast, IFN-β treatment increased the number of GFAP-positive cells in the brains of wild-type mice [209] and in primary mouse astrocyte cultures [230]. In addition, in increased major histocompatibility complex class I (MHC-I), protein levels were found in IFN-β-treated NG97 astrocyte cells [231]. In addition, the protein levels and secretion of A1 astrocyte-related factors such as CXCL10, IL-6, CCL5 increased both in vitro [230] and in vivo [232] following the treatment with IFN-β or the induction of IFN-β via poly I:C administration. Moreover, IFN-α increased the levels of CCL2, IL-6 and CXCL10 secreted by astrocytes in the co-cultures of human monocytes and astrocytes [233]. 

In addition to its effects on astrocyte reactivity, IFN-β affects the survival of astrocytes, e.g., in stressed rat fetal astrocytes, the administration of IFN-β itself increased apoptosis, but reduced TNFα-induced apoptosis [234]. However, a decreased level of apoptosis of primary rat fetal astrocyte cultures or the lack of an effect of IFN-β on cell survival in stressed primary rat neonatal astrocytes has also been reported [235]. These contradictory results may be explained by the fact that high doses of IFN-β induced cell death in rat fetal astrocytes via decreased levels of p38 MAPK, while low doses increased the number of BrdU-positive and thus proliferating cells via an increase in pAKT levels [236,237]. 

As in microglia cells, in astrocytes IFNs show an effect on the pro-inflammatory and apoptosis-related pathways STAT1/21 and NFκB. Both IFN-β and IFN-α increased the STAT1/2 expression levels and the degree of phosphorylation of STAT1/2 in EAE mice [222] as well as in human fetal astrocytes [222], primary mouse astrocyte cultures [217] and mixed mouse glial cultures [209]. IFN-β also increased the levels of nuclear p65 NFκB in the mouse EAE model [222], and high doses of this IFN induced cell death and decreased the protein levels of the NFκB-inhibitor IκB in serum-starved primary rat fetal astrocytes [236]. 

Taken together, IFNs may induce either a neurotoxic A1 or neuroprotective A2 astrocytic phenotype, depending on the specific disease-induced microenvironment.

#### 2.4.3. Neurons

In the rat EAE model of MS, IFN-β-1b reduced clinical disease activity, but did not result in a protective effect on RGCs [160]. Similarly, MRS-measured levels of the neuronal integrity markers NAA, Cr, pCr and glutamate were also not modulated in the brains of RRMS patients treated with IFN-β [185,238] even though the relapse rate was reduced [238]. However, increased NAA/Cr ratios, indicative of reduced neuronal injury, have also been found in the normal appearing white matter (NAWM) of MS patients treated with IFN-β-1b for 24 months [239,240]. Moreover, IFN-β-1a caused a slight decrease in the loss of RGCs in a mouse MOG-induced optic neuritis model [207]. The exposure of neuroblastoma SH-SY5Y cells in culture to IFN-α2 enhanced Cr, lactate and osmotic balances, and levels of metabolic waste products in the growth medium [241], which may have a positive effect on neuronal survival.

The literature is contradictory regarding the effect of IFNs on the protection of neurons from direct injury. In vitro, IFN-β and IFN-α increased neuronal cell survival after the human herpesvirus 1 (HSV-1) infection of mouse neuronal cell cultures [242] or La Crosse Virus infection of human cerebral brain organoids [243]. Nonetheless, IFN-β did not protect primary mouse neuronal cultures against viral infection [244] and IFN-β-1a showed no neuroprotective effect on rat RGCs [207]. In addition, IFN-β did not affect NDMA- or α-amino-3-hydroxy-5-methyl-4-isoxazolepropionic acid receptor (AMPA)-induced cell death in mouse neuron–microglia co-cultures, but did attenuate the microglia-induced cell death of mouse neurons [216], suggesting that the effects of IFNs on neurons may be generated via microglia cells.

At a more physiological level, IFN-α reduced the firing rate of neurons via opiate receptors in the rat hypothalamus, normalizing their neuronal response [245]. Furthermore, levels of the synapse-associated protein synaptophysin (SYP) were reduced in PC12 cells, following the incubation with IFN-β [246]. Supporting this notion, rIFN-β reduced the levels of the synaptic protein PSD95 and the dendritic spine density in wild-type mice, via the induction of complement component 3 (C3)-dependent synapse elimination [209], again pointing towards the microglia-mediated effects of IFNs on neurons.

IFN-β affected the phosphorylation levels of STAT1 [242], MAPK1/2 [207] and JAK1 [242] and total STAT1 protein levels [244] in primary rodent neuronal cultures. In addition, unlike normal neuronal cells, the exposure of STAT1-mutated neurons to IFN-β did not render them resistant to HSV-1 infection [247], which indicates that this IFN indeed affects neuronal STAT1 signaling. Still, the low expression of STAT1 in neurons [244] might explain why IFN-β does not have such profound effects on neurons compared to microglia and astrocytes.

#### 2.4.4. Oligodendrocytes

In various inflammatory and non-inflammatory demyelination-induced rodent models, IFN-β reduced demyelination in the brain and spinal cord, as was evident from the increased levels of myelin-related proteins [207,220,248]. Furthermore, the serum of untreated MS patients and of patients treated with IFN-β for six months inhibited OPC proliferation in mixed rat glial cultures, while the serum of twelve-month IFN-β-treated patients did not show this effect [249]. Furthermore, in mixed rat glial cultures and primary OPCs, IFN-β did not affect OPC differentiation or proliferation, but did not showed cytotoxic effects on the oligodendroglia cell line OLN-93 [250]. In primary rat oligodendrocytes, IFN-β did attenuate neurogenic locus notch homolog protein 1 (NOTCH-1) and SHH signaling, which are main factors for OPC maturation [220]. Importantly, the inhibition of rat OPC differentiation by IFN-β was only found in the presence of astrocytes and microglia cells [250]. As in neurons, this may again highlight the importance of astrocytes and microglia for IFN-induced effects on oligodendrocytes.

In EAE mice, IFN-β significantly reduced multiple oxidative-stress parameters (malondialdehyde, peroxidation potential, ratio of catalase (CAT) to superoxide dismutase (SOD), and blood GSH), and also improved the redox state of the animals [251], which may indirectly result in an improved white-matter repair response since OPCs are very sensitive to oxidative stress [252]. Furthermore, IFN-β did not show cytoprotective effects toward injury induced by H_2_O_2_, NO, complement or glutamate [250]. These results indicate that the limited effect of IFN-β on oligodendrocytes may occur via a reduction of oxidative stress and through its influence on other cell types such as astrocytes and microglia.

### 2.5. Molecular Effects of TF

TF is the active metabolite of its parent drug leflunomide and is FDA approved for the treatment of MS. TF inhibits the mitochondrial enzyme dihydro-orotate dehydrogenase (DHODH), blocking pyrimidine synthesis and leading to cell cycle interruption in T- and B-cells. Another peripheral effect of TF concerns the decrease in pro-inflammatory IL release from monocytes [253]. In the brain, TF reduced BBB damage by increasing the number of pericytes, and the levels of the tight-junction proteins Zonula occludens protein-1 (ZO-1) and occludin (OCLN) [254]. Moreover, in the brain, TF targets circulating levels of pyrimidine and purine nucleotides, and of GSH and carbohydrate metabolism intermediates [255]. These molecules are linked to microglia activation, glutamate excitotoxicity and neuronal viability, which implies that TF may have an important impact on MS CNS pathology. Here, we review the specific effects of TF on microglia, neurons and oligodendrocytes (for details, see Supplementary Table S5; for article search terms, see Supplementary Information).

#### 2.5.1. Microglia

A reduction in the number of pro-inflammatory M1 Sialoadhesin (SIGLEC-1)-positive or IBA1-positive microglia following TF treatment has been reported in rodent models for various diseases, such as in the brains of mice suffering from neuronal ceroid lipofuscinosis (CLN) [49], in the hippocampus of two different rat models for traumatic brain injury (TBI) [256], in the basal ganglia and the corpus callosum of a Theiler’s murine encephalomyelitis virus (TMEV)-induced mouse demyelination model [257], and fewer IBA1-positive cells were also detected in the brains of transient middle cerebral artery occlusion (tMCAO) stroke mice [254]. At high concentrations, TF diminished the LPS- and IFN-γ induced CD68 expression in vitro [258], and following LPS stimulation, the expression of the M2 microglia cytokine IL-10 was slightly increased in rat primary microglia and mixed glial cultures [258]. Furthermore, TF decreased the expression of the pro-inflammatory cytokines CXCL10, CCL2 and IL-6 in human microglia–monocyte co-cultures stimulated with HIV particles [146], and lower levels of the pro-inflammatory proteins IL-1β, COX2 and 3-NT were detected following the TF treatment of mice with tMCAO-induced stroke [254]. However, in primary rat microglia cultures, no effect of TF was observed on the mRNA expression levels of iNOS, IL-6 and TNFα and on the protein level of NFκB inhibitor IκBα [258]. These results suggest that TF decreases microgliosis and reduces the M1 pro-inflammatory microglia phenotype, although the molecular pathway involved is presently unclear.

#### 2.5.2. Astrocytes

To our knowledge, there are at present no reports describing the effect of TF on astrocytes in culture or in in vivo models.

#### 2.5.3. Neurons

The TF treatment of a mouse model of CLN resulted in a reduced number of damaged SMI32-positive axons, a reduced loss of RGCs and less thinning of the retina [49]. Axonal loss was also diminished by TF in the CNS of TMEV-demyelination mice [257]. In addition, TF treatment stimulated neurogenesis in the hippocampal sub granular zone of TBI rats [256], and upregulated the number of BrdU-/DCX-positive cells and the expression of mammalian achaete-schute homolog 1 (MASH1), DCX and PBX homeobox 1 (PBX1) in the sub ventricular zone of the mouse tMCAO stroke model [254]. In vitro, the neurotoxic effect of the medium from HIV-particle-stimulated microglia–monocytes co-cultures on human fetal neurons was attenuated by TF [146]. These studies indicated that TF may affect neurogenesis and neuronal survival.

#### 2.5.4. Oligodendrocytes

In mice with TMEV-induced demyelination, TF did not increase the number of OLIG2- and CC1-positive oligodendrocyte lineage cells nor the myelin density when compared to the vehicle-treated animals [257]. In rat neuron–oligodendrocyte co-cultures, only low doses of TF led to cell-cycle exit, while high doses resulted in the reduced survival of oligodendrocytes [259]. The same study showed that only short-term pulses of TF promoted OPC differentiation towards CNPase-, MOG- and MBP-expressing cells in vitro [259]. These results indicate that the effects of TF on oligodendrocytes are modest and are possibly modulated by the concentration of TF or the duration of TF treatment.

### 2.6. Molecular Effects of LQ

LQ is an orally administered, synthetic derivative of linomide, that targets peripheral immune cells, such as activated IL-21-producing CD4^+^CD44^+^ T-cells, C11c^+^CD4^+^ dendritic cells dendritic cells and B-cells [260], but it also displays neuroprotective effects in the mouse EAE model [30,31]. In addition, the linomide derivative might work via the AhR because LQ-treated mice showed major transcriptional changes of genes downstream of this receptor [261], such as the AhR hydroxylase Cytochrome P450 (CYP1A1). Here, we deal with the specific molecular effects of LQ on CNS cell types (for details, see Supplementary Table S6; for article search terms, see Supplementary Information).

#### 2.6.1. Microglia

In rodent models, for both the acute and chronic type of EAE, as well as in rodent models in which non-immunological demyelination is induced in the CNS, optic nerve or retina, LQ reduced the density of IBA1-positive M1 microglia [261,262,263,264,265]. In EAE mice, mRNA and protein expression levels of IBA1, transmembrane protein 119 (TMEM119) and CD68 were also reduced by LQ [261]. Importantly, in AhR-knockout mice, no effect of LQ on the number of IBA1-positive cells was found [262], indicating that AhR is the main target of LQ. LQ also decreased the number of MAC-3- and CD45-positive microglia cells in cuprizone mice, EAE mice and mouse TLR4-knockout and myeloid differentiation primary response 88 (MyD88)-knockout demyelination models [261,265,266,267,268,269]. Moreover, LQ increased the protein level of the pro-inflammatory microglia marker translocator protein (TSPO) [267] in the CNS of cuprizone-exposed mice [265]. Nevertheless, an increase in TSPO in microglia cells is not necessarily associated with neurodegeneration [270]. The expression levels of factors that are associated with microglial inflammation, such as TNFα, IL-1β, IL-6 and MMP-9, were reduced in LQ-treated human and mouse microglia cultures [263]. However, LQ also reduced the levels of anti-inflammatory interleukins IL-10 and IL-4 in these microglia cultures [263], raising the question of which exact microglia phenotype it induces.

LQ further reduced the activity of c-Jun N-terminal kinase (JNK), ribosomal S6 kinase and AKT pathways in primary human LPS-stimulated microglia cultures, and lowered nitrite levels in mouse microglia–neuron co-cultures [263]. The microglia mRNA expression profile of an LQ-treated mouse model of TBI was similar to that of the sham (non-TBI) group, indicating that LQ reduces TBI-pathology [271]. However, in the mouse cuprizone model, LQ reduced pro-inflammatory factors via NFκB signaling only in astrocytes, but not in microglia cells [268]. Finally, LQ upregulated levels of microRNA 124a (miR-124a) [263], which targets a number of molecular pathways that preserves a microglia inflammatory state and was recently proposed as a potential biomarker for CNS diseases [272].

We concluded that LQ reduced the pro-inflammatory M1 phenotype of microglia cells in several CNS-located pathologies.

#### 2.6.2. Astrocytes

Reactive gliosis, characterized among others by an increase in the number of GFAP- and vimentin (VIM)-positive cells, was reduced following the LQ treatment of cuprizone mice [265,268] and in the EAE-induced mouse demyelination model [261,262,266] with again no effect in AhR-knockout EAE mice [262]. One study has reported that LQ did not affect reactive astrocytes in EAE mice [269]. In vitro, LQ reduced the expression of the pro-inflammatory cytokines IL-6 and CXCL10 in IL-1β- and IFN-γ-stimulated primary human astrocytes, but induced the protein expression of the chemokine CCL5 [273], a potential modulator of central glutamatergic transmission which is important for glial–neuron crosstalk [274]. Moreover, in IL-1β-stimulated mouse astrocyte cultures, LQ increased the CCL5 mRNA and protein levels, and reduced the mRNA and protein levels of TNFα, CXCL10 and IL-6 [268]. The expression of these pro-inflammatory cytokines was diminished by LQ via its inhibitory effect on NFκB activation in primary astrocyte cultures from TRL4-, MyD88- or Toll-like receptor adaptor molecule 1 (TRIF)-knockout demyelination mice [267] and cuprizone-fed mice [268]. Furthermore, LQ reduced both the NFκB levels and p65 nuclear translocation in IL-1β-stimulated primary mouse astrocytes [268]. In sum, LQ attenuates the reactive A1 astrocytic phenotype and its pro-inflammatory downstream signaling events, possibly via NFκB signaling.

#### 2.6.3. Neurons

Several neuronal parameters appear to be positively affected by LQ treatment. Multiple studies have shown that in rodents, LQ reduces the axonal injury inflicted by EAE [262,263,266,269] and cuprizone [264,265,267,268], and after TBI [271] and neurodegeneration in the mouse R6/2 HD model [275] and genetically induced demyelination models [267]. Again, no effect was found on the number of APP-positive axons in LQ-treated AhR-knockout mice [262]. Moreover, when given prophylactically, LQ protected RGCs and reduced the apoptosis of neurons via the diminished cleavage of CASP3 in the mouse EAE model [261]. The effect of LQ on neuronal apoptosis may be explained by its reduction of BCL2-associated X (BAX) levels, which is a pro-apoptotic protein causing mitochondrial cytochrome-c release and caspase activation [276].

Not only was neuronal mortality reduced by LQ, but also the number of DCX-positive cells increased in the TBI mice [271], which suggests increased neurogenesis and maturation. This is in line with LQ-studies reporting increases in NeuN-, protein phosphatase 1 regulatory subunit 1B (DARPP-32)- and BDNF expression in neurons of the mouse R6/2 HD model [275] and in total brain tissue from wild-type mice [277]. Moreover, in human and mouse neuronal and microglia co-cultures, LQ increased the number of mature neuronal microtubule-associated protein 2 (MAP2)-positive cells [263]. Thus, LQ might affect neuronal injury via diminishing apoptotic signaling or increasing neuronal differentiation.

Furthermore, LQ alters the physiological response of neurons. In the mouse EAE model, both preventive and therapeutic LQ treatment changed γ-aminobutyric acid (GABA)ergic synaptic transmission, while only preventive LQ affected glutamatergic synaptic transmission as well, resulting in reduced glutamatergic excitotoxicity [269]. In line with this, LQ reduced the number of APP-positive axons, specifically of vesicular glutamate transporter 1 (VGLUT1)-positive glutamatergic neurons, and reduced the number of SYP-positive axonal spheroids in cuprizone-fed mice [265]. The neuronal output of the inner layer and b-wave amplitudes, as well as the callosal axon conduction and axonal refractoriness [266] also improved following the LQ treatment. In the co-cultures of mouse microglia and neurons, LQ decreased microglia-induced neurotoxicity [263] and the number of cells positive for iNOS in the mouse R6/2 HD model [275], indicating a positive effect on the degree of neurotoxicity and oxidative stress. However, in vitro, LQ had no effect on the expression of the chemo-attractants CXCL12 and CXCL88 in NSCs and it did not affect NSC differentiation after IL-1β stimulation [273]. Finally, and most relevant to HD pathology, LQ decreased the number of mutant-Huntingtin-positive PC12 cells, and diminished ATP production, proton leakage and basal respiration levels in the cell line [275]. The effects of LQ on neurons therefore include a reduction in axonal injury, an increase in neurogenesis, and an attenuation of GABAergic and glutamatergic synaptic transmission.

#### 2.6.4. Oligodendrocytes

The clearly positive effects of LQ on the degree of demyelination, in models for various CNS-related pathologies, indicate that this synthetic drug improves myelination when applied as a prophylactic, as well as a therapeutic treatment in both mouse EAE and cuprizone models [261,262,263,264,265,268,269,278]. Moreover, in EAE mice, an increase in axonal conduction has been observed, in line with improved myelin integrity [266]. However, in one study, therapeutic LQ treatment did not affect optic nerve demyelination in EAE mice [261]. The effect of LQ on demyelination may be explained by the fact that in vivo, the linomide derivative reduced the apoptosis of oligodendrocytes [268]. Nonetheless, LQ did not affect the survival or mitochondrial respiration of primary mouse OPC cultures [268] or human ESC-derived OPCs stimulated with IL-1β [273]. Furthermore, based on the unaltered number of neural/glial antigen 2 (NG2)-, CNPase- and MBP-positive oligodendrocytes, LQ did not affect in vitro OPC differentiation [273]. In contrast, LQ did increase the number of oligodendrocytes positive for the cytoskeletal-related protein, the tubulin polymerization-promoting protein (P25) [267], which is in general highly upregulated in differentiating primary oligodendrocytes [279]. In EAE mice, MBP-, CC1 and PLP1-positive oligodendrocyte numbers increased after LQ treatment [266]. Finally, the treatment of oligodendrocytes with LQ had no effect on the expression of the chemo-attractants CXCL8 and platelet-derived growth factor subunit A (PDGF-A) [273]. Taken together, the treatment with LQ before as well as after the induction of demyelination appears to have a positive effect on the degree of myelination and myelin integrity, possibly via reducing oligodendrocyte apoptosis or via stimulating OPC differentiation.

### 2.7. Molecular Effects of NZ

NZ is a humanized monoclonal IgG4 antibody against the alpha-4 subunit of very late antigen-4 (VLA4) of α4β1 and α4β7 integrins [280] that are located on the plasma membrane of lymphocytes and monocytes. NZ is effective in RRMS patients and mainly prevents the infiltration of leukocytes into the MS brain [281], but also more specific effects on immune cells and hematopoietic populations in the CNS have been described [282]. Furthermore, NZ has important additional effects on the MS-affected CNS, including a reduction of oxidative stress and LPS-binding protein (LBP) levels [283], as wells as CSF levels of markers for intrathecal inflammation, axonal damage and demyelination [29]. In the following paragraphs, we discuss the effects of NZ on microglia, astrocytes, neurons and oligodendrocytes (for details, see Supplementary Table S7; for article search terms, see Supplementary Information).

#### 2.7.1. Microglia

Overall, most studies point to an effect of NZ on the attenuation of microgliosis by reducing the activated M1 microglia response in various disease models, such as in the APP1/PS1 mouse model of AD [284] and in EAE mice [285]. The NZ treatment of MS patients also reduced the pro-inflammatory microglia phenotype, as established by PET scanning, using the pro-inflammatory microglia marker 11C-PK11195, which binds to the mitochondrial outer-membrane translocator protein TSPO [286,287]. Moreover, CSF levels of soluble triggering receptor expressed on myeloid cells 2 (sTREM-2) [288] and the chitotriosidase (CHIT1) [289], markers for pro-inflammatory microglia [290,291], were decreased following NZ-therapy in MS patients. Thus, NZ has an effect not only on peripheral, but also on brain-resident immune cells, possibly because microglia express the NZ-antigen integrin VLA-4 when phagocytosing neuronal debris [292].

#### 2.7.2. Astrocytes

Astrogliosis was reduced in a mouse AD model treated with NZ, when compared to untreated animals and based on the number of reactive A1 GFAP-positive astrocytes and the protein expression level of GFAP [284]. Moreover, following the NZ treatment of EAE mice, the protein levels of the inflammation-associated protein lipocalin 2 (LCN2) [293,294] was reduced in inflammation-activated GFAP-positive astrocytes [295]. The CSF and the CNS of both EAE mice and MS patients also contained elevated LCN2 levels, which were modulated by NZ treatment [295]. Interestingly, the reactive astrocyte-confined expression of CXCL12 was downregulated in NZ-treated EAE mice [282], suggesting that in addition to reducing the reactive astrocytic phenotype, the drug also affects downstream chemokine signaling.

#### 2.7.3. Neurons

In the mouse EAE model, NZ treatment resulted in more NF-positive cells, indicating reduced axonal damage [285]. Interestingly, NZ normalized the synaptic changes found in the mouse APP/PS1 model of AD by increasing the PSD-95 levels [284]. Moreover, RRMS patients treated with NZ showed an increase in the concentrations of NAA, Cr, pCr and glutamate in lesioned white matter [185]. These findings point to an effect of NZ on axonal and mitochondrial metabolism.

#### 2.7.4. Oligodendrocytes

NZ reduced the inflammation-mediated demyelination in EAE mice, as indicated by the increases in the number of MBP-positive cells [285]. In line with this, LCN2, which in vivo is decreased by NZ, inhibits remyelination in rat neuron–oligodendrocyte co-cultures [296]. Possibly, NZ affects remyelination by oligodendrocytes, but more studies are needed.

### 2.8. Molecular Effects of AZ

AZ is a humanized monoclonal antibody against the 12-amino acid cell-surface glycopeptide cluster of differentiation 52 (CD52), which is an antigen found on the surface of B- and T-lymphocytes [297], but not on the surface of hematopoietic stem cells. The extent to which certain immune cell subpopulations are affected by AZ differs due to the variable surface-expression levels of CD52 [298]. The function of CD52 is largely unknown, but this glycosylphosphatidylinositol (GPI)-anchored glycopeptide is thought to contribute to the activation and migration of T-cells, and the induction of regulatory T-cells [299]. Moreover, CD52 was recently found to be expressed on the surface of microglia cells under neuropathological conditions in the mouse [300]. We now discuss the effects of AZ on CNS cell types (for details, see Supplementary Table S8; for article search terms, see Supplementary Information).

#### Microglia, Astrocytes, Neurons and Oligodendrocytes

The number of studies on the effects of AZ on CNS cells is limited. In hippocampal slice cultures of EAE mice, the blockade of CD52 with anti-mouse CD52 antibody triggered microglia morphology towards a less ramified, and thus possibly more activated M1 phenotype [301], while this antibody did not affect neuronal Ca^2+^ levels or neuroprotection against excitotoxicity [301]. To our knowledge, there are at present no reports describing the effect of AZ on astrocytes and oligodendrocytes, in culture or in in vivo animal models. AZ may have beneficial effects on CNS-resident glial populations, since CD52 expression is mainly found on microglia, and to a lesser extent on astrocytes, and is upregulated following the in vitro LPS treatment of primary mouse microglia cells [301].

### 2.9. Molecular Effects of OCR

OCR is a humanized monoclonal IgG1 antibody against an extracellular domain of the glycosylated protein cluster of differentiation 20 (CD20), that is expressed on pre-B-cells, mature B-cells and memory B-cells [302]. OCR is the most recently approved treatment for both RRMS and primary-progressive MS (PPMS). The main function of OCR is the elimination of B-cells from peripheral blood, primarily through antibody-dependent cellular cytotoxicity (ADCC) and to a lesser extent by antibody-dependent cellular phagocytosis (ADCP), complement-dependent cytotoxicity (CDC) and the direct apoptosis of B-cells [303,304]. In the next paragraph, we review the effects of OCR on CNS cell types (for details, see Supplementary Table S9; for article search terms, see Supplementary Information).

#### Microglia, Astrocytes, Neurons and Oligodendrocytes

The effects of OCR have been studied only in astrocytes and neurons. OCR displayed a superior effect, relative to IFN-β, with respect to reducing gliosis in the mouse EAE model as well as in MS patients [208]. In a recent MRS study on RRMS patients, OCR was found to reduce gliosis, as shown by a reduction in the gliotic inflammation marker myo-inositol, and stabilized the total choline and total Cr levels in the brain, indicating a decreased neurotoxic and inflammatory environment [208]. Moreover, in two rat MS models, anti-CD20 therapy reduced the number of MHC-II-positive microglia and decreased the binding of the TSPO-radioligand (^125^I)DPA-713, indicating decreased microgliosis [71]. However, anti-CD20 treatment did not result in decreased numbers of MAC3-positive microglia nor in reduced demyelination in an MS mouse model [305]. To our knowledge, there are at present no other reports describing the effect of OCR on cells in culture or in in vivo animal models, possibly since this antibody is one of the most recent MS drugs to have become FDA approved. 

## 3. Discussion

In the previous sections, the effects of various FDA-approved drugs for MS on microglia, astrocytes, neurons and oligodendrocytes were described on the basis of results from studies on cells in culture, in animal models and in humans. Of note, any discrepancies in the extent of the effects reported in the various studies may be attributed to differences in the cell lines or species used, the methods of drug administration and/or the concentrations of the drugs applied. Most evident were the actions of the drugs regarding the modulation of the pro-inflammatory downstream cascades within microglia and astrocytes, and we therefore discuss in particular their effects on these CNS cell types. Since microglia and astrocytes are involved in maintaining the neuronal network [306] and the quality of myelin [307], we also deliberate on how their drug-induced changes may affect neurons and oligodendrocytes. Finally, we consider how microglia and astrocytes may influence the inflammatory state of each other.

### 3.1. FDA-Approved MS Drugs Induce the Transition from a Pro-Inflammatory into an Anti-Inflammatory Microglia Phenotype 

FTY720, the synthetic analogue of sphingosine, reduced in vitro and in vivo the secretion of pro-inflammatory cytokines and the expression of pro-inflammatory factors in microglia. However, in the mouse cuprizone-demyelination model, FTY720 did not in all investigations switch microglia to an anti-inflammatory phenotype. The fumaric acid ester DMF, and the active leflunomide metabolite TF, also reduced the number of activated M1 microglia in in vivo models of inflammation or otherwise induced CNS pathology. The DMF studies that showed a reduction in the expression and secretion of pro-inflammatory factors were mainly performed in vitro, while the TF studies were both in cell cultures and in rodent models. Treatment with the synthetic polypeptide mixture GA caused not only a reduction in pro-inflammatory cytokine expression and secretion, both in vitro and in vivo, but also an increase in neuroprotective cytokine expression and a reduction in the number of microglia with a pro-inflammatory M1 phenotype. In addition, GA may stimulate phagocytosis by microglia cells. For unknown reasons, the naturally occurring cytokines IFN-β and IFN-α caused in (un)stressed cell cultures, as well as in wild-type rodents and rodent CNS-related disease models, the conversion of microglia cells into either a pro-inflammatory M1 or an anti-inflammatory M2 phenotype. However, the IFN-β studies performed in vitro point more to an induction of the expression of pro-inflammatory cytokines and other factors. The synthetic linomide derivative LQ as well as the humanized monoclonal antibody NZ reduced the number of pro-inflammatory microglia cells in animal studies and in MS patients. Unfortunately, no studies, or only a very limited number of studies, on the humanized monoclonal antibodies AZ and OCR have been reported with respect to their impact on microglia.

**Figure 1:**
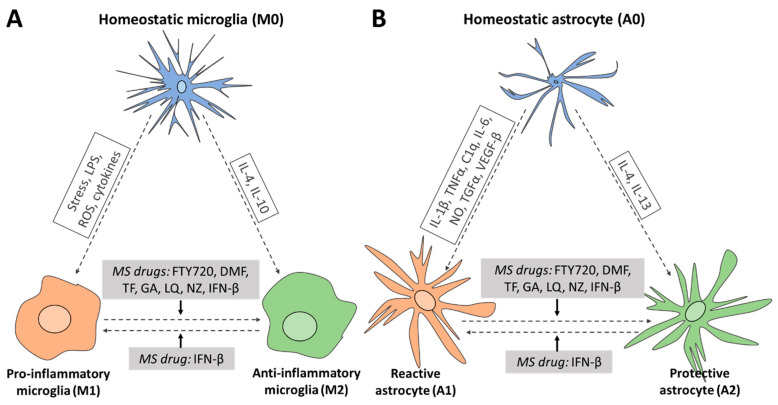
Effects of the environmental cues and the FDA-approved multiple sclerosis (MS) drugs on (**A**) the homeostatic M0 microglia becoming pro-inflammatory microglia (M1) or the anti-inflammatory microglia (M2) and (**B**) the homeostatic A0 astrocytes becoming reactive astrocytes (A1) or neuroprotective astrocytes (A2). Black dotted arrows indicate a change in phenotype. Black solid arrows indicate influence of MS drug on the change in phenotype. FTY720: Fingolimod, DMF: Dimethyl Fumarate, TF: Teriflunomide, GA: Glatiramer Acetate, LQ: Laquinimod, NZ: Natalizumab, IFN-β: Interferon-β.

Altogether, the in vitro and in vivo findings indicate that, except for IFN-β, the various FDA-approved MS drugs described here have a clear effect on the transition from a pro-inflammatory into an anti-inflammatory microglia phenotype (Figure 1A). This effect is particularly evident in inflammatory (EAE) as well as non-inflammatory (Cuprizone, Lysolecithin, TBI, AD) rodent models in which neurotoxicity and demyelination are central factors causing pathology. In general, the results of the in vitro studies are in line with this inference. We argue though that more studies should be performed, especially on AZ and OCR.

### 3.2. FDA-Approved MS Drugs Induce the Transition from a Reactive into a Neuroprotective Astrocytic Phenotype

Of the drugs and CNS cell types reviewed here, most clear are the in vitro and in vivo effects of FTY720 on astrocytes. FTY720 reduced the secretion of pro-inflammatory cytokines and the reactive A1 phenotype of astrocytes. Similarly, DMF decreased the levels of pro-inflammatory cytokines secreted by astrocyte cultures and the reactive A1 astrocytic phenotype in vivo. Moreover, DMF showed clear effects on the oxidative stress response of astrocytes. As holds for the microglia phenotype, both IFN-β and IFN-α modulated the astrocytic phenotype towards either a neurotoxic A1 or a neuroprotective A2 state. In addition, the two IFNs may also stabilize the BBB. Both GA and LQ decreased the astrocytic A1 reactivity in vivo, and reduced the secretion of pro-inflammatory cytokines by cultured astrocytes. Astrogliosis, as well as the expression of pro-inflammatory-linked metabolites and factors in animal models and human studies, appeared to be diminished by both NZ and OCR, although the number of pertinent studies is limited. To our knowledge, there have been no studies on the effects of TF and AZ on astrocytes. In sum, most FDA-approved MS drugs demonstrate the capacity to reduce the inflammatory environment in the CNS, by converting reactive A1 astrocytes into a neuroprotective A2 phenotype (Figure 1B). Comparable to what holds for microglia, this effect was evident from in vitro cellular studies as well as from studies on wild-type rodents, rodent CNS-related disease models and MS patients.

### 3.3. Effects of FDA-Approved MS Drugs on the Microglial and Astrocytic Phenotypes May Be Mediated through NFκB Signaling

**Figure 2:**
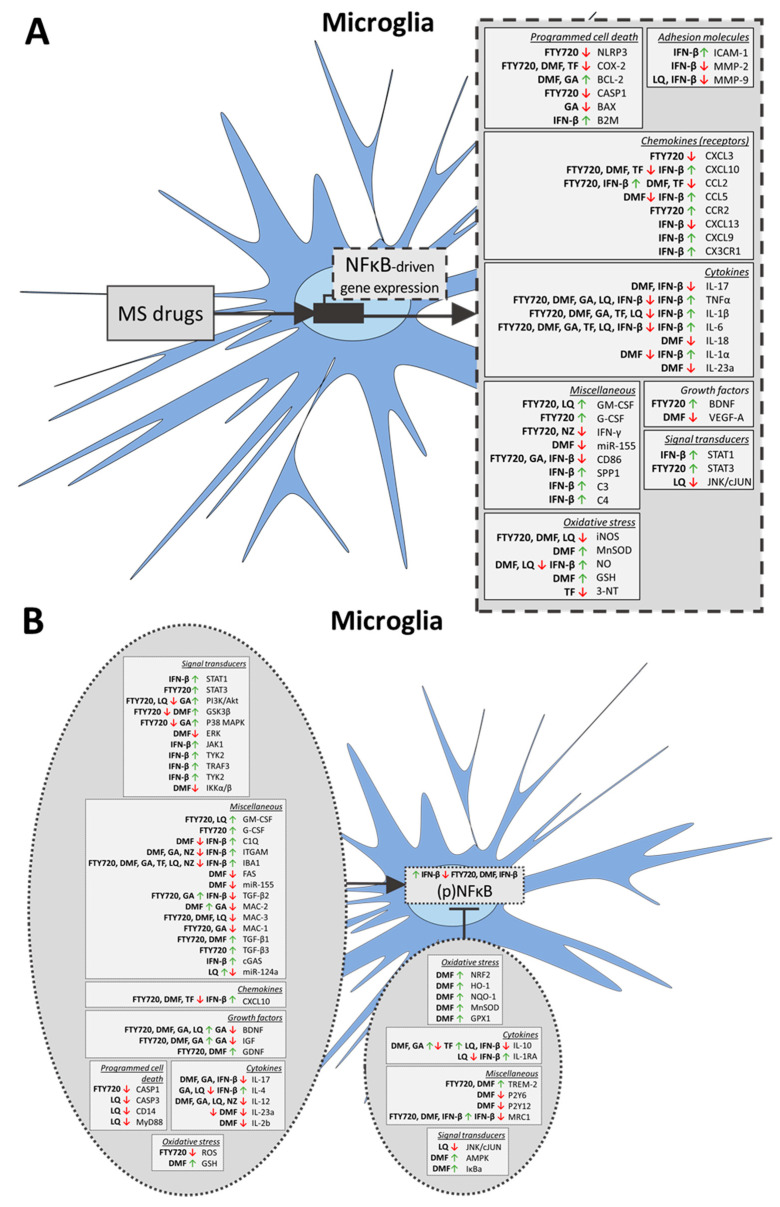
MS drugs directly or indirectly target NFκB signaling in microglia. The effects of MS drugs on (**A**) NFκB-driven gene expression and (**B**) the expression of genes known to affect NFκB signaling are shown. Black arrows: stimulation; bar-headed lines: inhibition; red arrows: decreased expression; green arrows: increased expression. Genes mentioned in A within the rectangular frame are under transcriptional regulation of NFκB. Genes mentioned in B within the circular frames are upstream regulators of NFκB.

**Figure 3:**
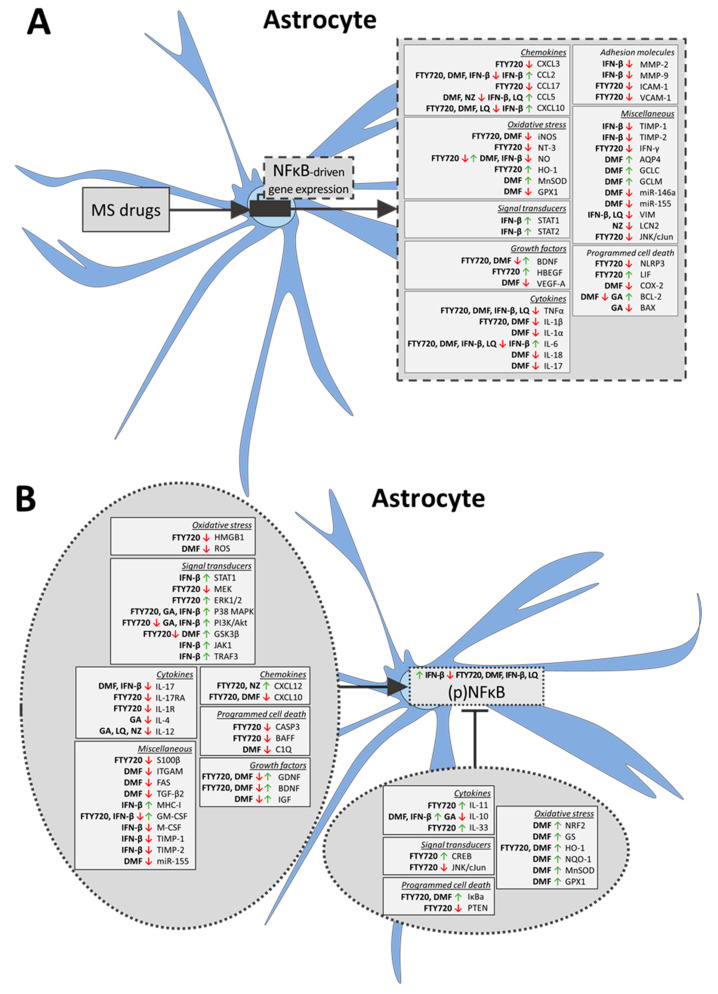
MS drugs directly or indirectly target NFκB signaling in astrocytes. The effects of MS drugs on (**A**) NFκB-driven gene expression and (**B**) the expression of genes known to affect NFκB signaling are shown. Black arrows: stimulation; bar-headed line: inhibition; red arrows: decreased expression; green arrows: increased expression. Genes mentioned within the rectangular frame are under transcriptional regulation of NFκB. Genes mentioned within the circular frames are upstream regulators of NFκB.

NFκB is the major signal transducer involved in the activation of microglia and astrocytes towards a pro-inflammatory M1 and reactive A1 phenotype, respectively [308]. This transcription factor complex, that binds to nuclear DNA elements, is responsible for activating the transcription of a wide range of pro-inflammatory cytokines, chemokines and matrix metalloproteinases, and induces oxidative stress and inflammasome activation [309,310]. Moreover, NFκB interacts and cooperates with two other nuclear signal transducers, STAT1 and STAT3 [78,79,311]. A number of studies has described an increased degree of phosphorylation of NFκB, STAT1 and STAT3 under various CNS disease and injury conditions, linking their activation to the induction of a number of pathological states [312,313,314,315]. The effects of the MS drugs on pro-inflammatory as well as anti-inflammatory factors, such as chemokines, growth factors, and oxidative stress inducers that are transcriptionally driven by NFκB (Figure 2A), strongly indicate that in microglia the NFκB pathway plays a key role in the molecular actions of these drugs. Moreover, in microglia, both FTY720 and DMF reduce the protein levels and activation of NFκB [44,55,58,77,127,129,132,143], and virtually all MS drugs modulate the expression of a remarkable number of molecular effectors upstream of NFκB, such as MAPK and PI3K/AKT [43,58,59,142,263], and other proteins known to influence NFκB signaling (Figure 2B). Similarly, in astrocytes most MS drugs discussed here affect the expression of pro-inflammatory and anti-inflammatory factors that is dependent on NFκB-induced transcriptional programs (Figure 3A). In addition, FTY720 and LQ diminish NFκB protein levels and activation [65,84,90,92,93,98,99,267,268], and nearly all the MS drugs modulate the expression of MAPK and ERK1/2 [91,107,120] as well as of other NFκB-interactor proteins in astrocytes (Figure 3B). Together, these findings highlight the importance of the NFκB pathway for MS drug actions in microglia as well as astrocytes. In this connection, one should realize that the effects of the monoclonal antibodies NZ, AZ and OCR on CNS cells have not yet been sufficiently studied. Remarkably, IFN-β seems to stimulate rather than inhibit the pro-inflammatory NFκB and STAT1 pathways [206,209,213,215,217], and the STAT1-related kinases JAK1 and TYK1 [215] in microglia and astrocytes [209,217,222,236].

### 3.4. Effects of FDA-Approved MS Drugs on Neurons and Oligodendrocytes May Be Caused Indirectly by the Attenuation of the Pro-Inflammatory States of Microglia and Astrocytes

Microglia and astrocytes have a wide range of functions in the CNS, not only at baseline conditions but also in response to neuroinflammation, including providing synaptic support and maintenance, preserving metabolic homeostasis, delivering structural support, and regulating oxidative stress, phagocytosis of cellular debris and neuroinflammatory responses to intruders [306,316,317,318,319]. As such, microglia and astrocytes support neurons and oligodendrocytes, but they also have important interactions with each other [320]. For example, activated M1 microglia induce reactive A1 astrocytes via the secretion of cytokines such as IL-1α, IL-1β and TNFα [321], as well as via the growth factors TGF-α and VEGF-B [322], and many other agents [320]. Conversely, reactive astrocytes increase the pro-inflammatory state of microglia by secreting factors such as CXCL10, CCL2 and LCN2 [320]. Thus, a bilateral communication occurs between microglia and astrocytes, which is important for maintaining a non-inflammatory microenvironment. In disease states, the prolonged presence of pro-inflammatory microglia and reactive astrocytes has detrimental effects on neurons and oligodendrocytes [306,307], and affects the BBB [323]. The positive effects of FDA-approved MS drugs on neurons, such as a reduction in axonal injury and neurotoxicity, and on oligodendrocytes, such as improving myelin integrity and remyelination capacity, may therefore reflect the indirect effects enabled by a reduction in the number of pro-inflammatory microglia and reactive astrocytes.

We realize that in addition to the M0/M1/M2 functional states, more types of pro- and anti-inflammatory microglia phenotypes exist and that these may have various downstream effects. For instance, the presence of microglia phenotypes associated with specific disease states, such as IFN- or LPS-induced microglia and neurodegeneration-associated microglia, has been reported [324]. Moreover, the inflammatory state of microglia may depend on their location in the brain (e.g., white matter versus grey matter) [325,326]. Likewise, diverse astroglia phenotypes apart from the A0/A1/A2 states have been found in a number of diseases [34]. 

## 4. Conclusions

In this review, we have provided an overview of the molecular effects of traditional and more recently FDA-approved MS drugs on four CNS cell types. The effects of these MS drugs on the peripheral immune system and their influence on immune cell infiltration via the BBB have been documented before, and were not specifically addressed here. From our comprehensive analysis of MS drug effects on CNS cells, we concluded that, via NFκB signaling, the majority of these drugs attenuates the pro-inflammatory M1 microglia and reactive A1 astrocytic phenotypes. On its turn, this attenuation may positively affect the functioning of diseased or injured neurons and oligodendrocytes. Such a mechanism is likely more complicated than initially thought in that microglia have recently been shown to engage a large range of activation programs which appear to be increasingly difficult to classify as purely pro-inflammatory versus anti-inflammatory [34,324]. Future characterization of the signatures of the recently described additional microglia and astrocytic subtypes, and of the extent of their pro-inflammatory or anti-inflammatory state, will aid in obtaining an even more detailed molecular understanding of the CNS mechanisms of action of drugs targeting MS. Knowledge of these molecular mechanisms may help anticipating adverse drug effects and in considering the use of combinatorial drug therapy to treat this complex neuroinflammatory disease.

## Supplementary Materials

Supplementary Materials can be found at https://www.mdpi.com/1422-0067/21/12/4229/s1.

## Abbreviations

3-NT	3-nitrotyrosine
AD	Alzheimer’s disease
ADCC	Antibody-dependent cellular cytotoxicity
ADCP	Antibody-dependent cellular phagocytosis
AhR	Aryl hydrocarbon receptor
AIM2	Absent in melanoma 2
AKT	Protein kinase B
AMD	Age-related macular degeneration
AMPA	α-amino-3-hydroxy-5-methyl-4-isoxazolepropionic acid receptor
APP	Amyloid precursor protein
ARG1	Arginase 1
ASC	Apoptosis-associated speck-like protein
AZ	Alemtuzumab
Aβ	Amyloid-beta
BAX	BCL2 associated X
BBB	Blood–brain barrier
BDNF	Brain derived neurotrophic factor
BrdU	Bromodeoxyuridine/5-bromo-2′-deoxyuridin
C3	Complement component 3
cAMP	Cyclic adenosine monophosphate
CAP	Compound action potentials
CASP	Caspase
CAT	Catalase
CCL	C-C Motif chemokine ligand
CD11c/ITGAX	Integrin alpha-X precursor
CD20	Cluster of differentiation 20
CD45	Protein tyrosine-protein phosphatase
CD52	Cluster of differentiation 52
CD68	Cluster of differentiation 68
CD86	Cluster of differentiation 86
CDC	Complement-dependent cytotoxicity
cGAS	Cyclic GMP-AMP synthase
c-Fos	FBJ murine osteosarcoma viral oncogene homolog
CHI3L3	Chitinase 3-like 3
CHIL3	Chitinase-like 3
CHIT1	Chitotriosidase
CLN	Neuronal ceroid lipofuscinosis
CNPase	2′,3′-Cyclic-nucleotide 3′-phosphodiesterase
CNS	Central nervous system
CNTF	Ciliary neurotrophic factor
COX2	Cyclooxygenase
Cr	Creatine
CREB	cAMP-response element binding protein
CSF	Cerebrospinal fluid
CXCL	Chemokine (C-X-C motif) ligand
CXCR	C-X-C Motif chemokine receptor
CYP1A1	Cytochrome P450
DARPP-32	Protein phosphatase 1 regulatory subunit 1B
DAT	Dopamine transporter
DCX	Doublecortin
DHODH	Dihydro-orotate dehydrogenase
DMF	Dimethyl fumarate
DRG	Dorsal root ganglion
EAE	Experimental autoimmune encephalitis
EGR1/2	Early growth response 1/2
EPSC	Excitatory postsynaptic currents
ERK1/2	Extracellular signal-regulated kinase 1 and 2
ESC	Embryonic stem cells
FDA	US Food and Drug Administration
FGF2	Fibroblast growth factor 2
FTY720	Fingolimod
FTY720-pPFTY720	Phosphorylated FTY720
GA	Glatiramer acetate
GABA	γ-aminobutyric acid
GAP43	Growth associated protein 43
GDNF	Glial cell line-derived neurotrophic factor
GFAP	Glial fibrillary acidic protein
GLI1	GLI family zinc finger 1
GM-CSF	Granulocyte-macrophage colony-stimulating factor
GMH	Germinal matrix hemorrhage
GPI	Glycosylphosphatidylinositol
GS	Glutamine synthetase
GSH	Glutathione
GSK3β	Glycogen synthase kinase 3 beta
H2O2	Hydrogen peroxide
HBEGF	Heparin binding EGF-like growth factor
HCAR2	Hydroxycarboxylic acid receptor 2
HD	Huntington’s disease
HDAC	Histone deacetylase
HIF-1α	Hypoxia-induced factor 1α
HMGB1	High mobility group box 1
HO-1	Heme oxygenase 1
HSV-1	Human herpesvirus 1
IBA-1	Allograft inflammatory factor 1
ICAM-1	Intercellular adhesion molecule 1
ICH	Intracerebral hemorrhage
IFN	Interferons
IFNAR	IFN receptor
IFN-α	Interferon-alpha
IFN-β	Interferon-beta
IFN-γ	Interferon-gamma
IGF-1	Insulin-like growth factor 1
IL	Interleukin
iNOS	Inducible nitric oxide synthase
IRF	Interferon regulatory factor
Iκbα	NFκB inhibitor alpha
JAK1	Janus kinase 1
JNK	c-Jun N-terminal kinase
JNK1/2	c-Jun N-terminal kinase 1 or 2
LBP	LPS-binding protein
LCN2	Lipocalin 2
LFB	Luxol Fast Blue
LIF	Leukemia inhibitory factor
LPS	Lipopolysaccharide
LQ	Laquinimod
MAC-1/ITGAM	Integrin alpha-M precursor
MAC-2	Galectin-3
MAC-3/CD107b	Lysosome-associated membrane protein 2
MAG	Myelin-associated glycoprotein
MAP2	Microtubule-associated protein 2
MAPK	p38 mitogen-activated protein kinases
MASH1	Mammalian achaete-schute homolog 1
MBP	Myelin basic protein
MHC-I	Major histocompatibility complex class I protein
MHC-II	Major histocompatibility complex class II protein
MiR-124a	MicroRNA 124a
MiR-155	MicroRNA 155
MMF	Monomethyl Fumarate
MMP	Matrix metalloproteinase
MnSOD	Manganese-dependent superoxide dismutase
MOG	Myelin oligodendrocyte glycoprotein
MOG-ON	MOG-induced optic neuritis
MPP	1-methyl-4-phenylpyridinium
MPTP	1-methyl-4-phenyl-1,2,3,6-tetrahydropyridine
MRC1/CD206	Mannose receptor C-type 1
MRI	Magnetic resonance imaging
MRS	1H-magnetic resonance spectroscopy
MRTF	MKL/megakaryoblastic leukemia 1
MyD88	Myeloid differentiation primary response 88
NAA	N-acetyl aspartate
NAWM	Normal appearing white matter
ND4	NADH dehydrogenase, subunit 4
NDMA	N-methyl-D-aspartate receptor
NeuN	Neuronal nuclei
NF	Neurofilament
NFκB	Nuclear Factor binding near the kappa-light-chain gene in B cell
NFκB p65	Rel-A
NG2	Neural/glial antigen 2
NGF	Nerve growth factor beta
NLRP3	NLR family pyrin domain containing 3
NO	Nitric oxide
NOTCH-1	Neurogenic locus notch homolog protein 1
NQO-1	NAD(P)H dehydrogenase quinone
NRF2	Nuclear factor erythroid 2-related factor 2
NSC	Neural stem cells
NT3	Neurotrophin-3
NZ	Natalizumab
OCLN	Occludin
OCR	Ocrelizumab
OGD	Oxygen–glucose deprivation
OLIG2	Oligodendrocyte transcription factor
OPC	Oligodendrocyte precursor cell
p	Phosphorylated
P204	Interferon-activable protein 204
P25	Tubulin polymerization-promoting protein
PAP	Placental alkaline phosphatase
PBX1	PBX homeobox 1
pCr	phosphoCr
PD	Parkinson’s disease
PDGF-A	Platelet derived growth factor subunit A
PI3K	Phosphoinositide 3-kinase
PLP1	Proteolipid protein
PPMS	Primary-progressive MS
PS1	Presenilin-1
PSD95	Postsynaptic density protein 95
PTZ	Pentylenetetrazol
PYHIN	Pyrin and HIN200 domain-containing proteins
RETNLA	Resistin-like alpha
RGC	Retinal ganglion cell
rIFN	Recombinant interferon
RIP3	Receptor-interacting protein kinase 3
ROS	Reactive oxygen species
RRMS	Relapsing-remitting MS
S100β	S100 calcium-binding protein B
S1P	Sphingosine 1-phosphate
S1PR1	S1P receptor 1
SCI	Spinal cord injury
SE	Status epilepticus
SHH	Sonic hedgehog
SIGLEC-1	Sialoadhesin
SLC1A	Solute carrier family 1 member
SMI32	Neurofilament-heavy chain
SMO	Smoothened
SOCS2	Suppressor of cytokine signaling 2
SOD	Superoxide dismutase
SPN2S	Spinster homolog 2
SREBP2	Sterol regulatory element-binding protein 2
SRF	Serum response factor
STAT	Signal transducer and activator of transcription
sTREM-2	Triggering receptor expressed on myeloid cells 2
SYP	Synaptophysin
TAU	Tau protein
TBI	Traumatic brain injury
TF	Teriflunomide
TGF-α	Transforming growth factor alpha
TGF-β2	Transforming growth factor-beta 2
TH	Tyrosine hydroxylase
TLR	Toll-like receptor
tMCAO	Transient middle cerebral artery occlusion
TMEM119	Transmembrane protein 119
TMEV	Theiler’s murine encephalomyelitis virus
TNF-α	Tumor necrosis factor alpha
TRIF	Toll like receptor adaptor molecule 1
TSPO	Translocator protein
TYK1	Leukocyte receptor tyrosine kinase
VCAM-1	Vascular cell adhesion protein 1
VEGF-B	Vascular endothelial growth factor B
VEGF-A	Vascular endothelial growth factor A
VGLUT1	Vesicular glutamate transporter 1
VIM	Vimentin
VLA-4/CD49d/CD29	Very late antigen-4
ZO-1	Zonula occludens protein-1
αSYN	α-synuclein
βIII-Tubulin	Beta-3-tubulin
