# Supplementary material for: Molecular Effects of FDA-Approved Multiple Sclerosis Drugs on Glial Cells and Neurons of the Central Nervous System
Source: Int J Mol Sci. 2020 Jun 13;21(12):4229. doi: 10.3390/ijms21124229 (PMC7352301; doi:10.3390/ijms21124229)
Supplement: Supplementary file 1 [file ijms-21-04229-s001.pdf]

Supplementary information

De Kleijn & Martens (2020): Molecular Effects Of FDA-Approved Multiple Sclerosis Drugs On Glial Cells And Neurons Of The Central Nervous System.

Search criteria

A systematic search to identify relevant studies was performed using PubMed and for each compound the following search-terms were utilized.

<u>Fingolimod:</u>
1) MS [tiab] AND brain [tiab] AND (Gileyna [tiab] OR Fingolimod [tiab] OR FTY720 [tiab])
2) (Gileyna [tiab] OR Fingolimod [tiab] OR FTY720 [tiab]) AND (axon [tiab] OR oligodendrocyte [tiab] OR astrocyte [tiab] OR microglia [tiab] OR pericyte [tiab] OR endothelial [tiab])

<u>Dimethyl Fumarate/Monomethyl Fumarate:</u>
1) MS [tiab] AND brain [tiab] AND (Tecfidera [tiab] OR Dimethyl Fumarate [tiab] OR DMF [tiab])
2) (Gileyna [tiab] OR Fingolimod [tiab] OR FTY720 [tiab]) AND (axon [tiab] OR oligodendrocyte [tiab] OR astrocyte [tiab] OR microglia [tiab] OR pericyte [tiab] OR endothelial [tiab])

<u>Glatiramer Acetate:</u>
1) MS [tiab] AND brain [tiab] AND (Glatiramer Acetate [tiab] OR Glatopa [tiab] OR Copaxone [tiab])
2) (Copaxone [tiab] OR Glatiramer Acetate [tiab] OR Glatiramer [tiab] OR Cop1 [tiab] OR Cop-1 [tiab]) AND (axon [tiab] OR oligodendrocyte [tiab] OR astrocyte [tiab] OR microglia [tiab] OR pericyte [tiab] OR endothelial [tiab])

<u>Teriflunomide:</u>
1) MS [tiab] AND brain [tiab] AND (Teriflunomide [tiab] OR Aubagio [tiab])
2) (Aubagio [tiab] OR Teriflunomide) AND (axon [tiab] OR oligodendrocyte [tiab] OR astrocyte [tiab] OR microglia [tiab] OR pericyte [tiab] OR endothelial [tiab])

<u>Interferon-beta/Interferon-alpha:</u>
1) MS [tiab] AND brain [tiab] AND (IFN [tiab] OR Interferon [tiab] OR Rebif [tiab] OR Avonex [tiab] OR Betaseron [tiab] OR Extavia [tiab] OR Plegridy [tiab]) NOT (IFN-γ [tiab] OR IFN-gamma [tiab] OR IFNgamma [tiab])
2) (IFN [tiab] OR Rebif [tiab] OR Avonex [tiab] OR Betaseron [tiab] OR Extavia [tiab] OR Plegridy [tiab]) NOT (IFN-γ [tiab] OR IFN-gamma [tiab] OR IFNgamma [tiab]) AND (axon [tiab] OR oligodendrocyte [tiab] OR astrocyte [tiab] OR microglia [tiab] OR neuron [tiab])

<u>Laquinimod:</u>
1) MS [tiab] AND brain [tiab] AND (Laquinimod [tiab] OR Nerventra [tiab])
2) (Laquinimod [tiab] OR Nerventra [tiab]) AND (axon [tiab] OR oligodendrocyte [tiab] OR astrocyte [tiab] OR microglia [tiab] OR pericyte [tiab] OR endothelial [tiab])

<u>Ocrelizumab:</u>
1) MS [tiab] AND brain [tiab] AND (Ocrelizumab [tiab] OR Ocrevus [tiab])
2) (Ocrelizumab [tiab] OR Ocrevus [tiab]) AND (axon [tiab] OR oligodendrocyte [tiab] OR astrocyte [tiab] OR microglia [tiab] OR pericyte [tiab] OR endothelial [tiab])

<u>Natalizumab:</u>
1) MS [tiab] AND brain [tiab] AND (Natalizumab [tiab] OR Tysabri [tiab])
2) (Natalizumab [tiab] OR Tysabri [tiab]) AND (axon [tiab] OR oligodendrocyte [tiab] OR astrocyte [tiab] OR microglia [tiab] OR pericyte [tiab] OR endothelial [tiab])

<u>Alemtuzumab:</u>

1) MS [tiab] AND brain [tiab] AND (Alemtuzumab [tiab] OR Lemtrada [tiab])
2) (Alemtuzumab [tiab] OR Lemtrada [tiab]) AND (axon [tiab] OR oligodendrocyte [tiab] OR astrocyte [tiab] OR microglia [tiab] OR pericyte [tiab] OR endothelial [tiab])

Studies identified by the systematic search were initially selected based on their title and abstract. Next, the completearticles were studied to extract the relevant information that was included when the following criteria were met:
- The study reports effects of the pertinent MS drug, or a commonly studied derivative of the MS drug (in the case of DMF/MMF, IFN-β/IFN-α);
- The study describes an effect on microglia, astrocytes, neurons and/or oligodendrocytes;
- In addition to any neurological measure of disease activity or progression, the study shows the effect of the pertinent MS drug at a molecular level.

Supplementary table 1. Molecular effects of Fingolimod (FTY20) on microglia, astrocytes, neurons and oligodendrocytes. ↑ indicates increased level of expression, number of cells or morphological/functional state; ↓ indicates reduced expression level, number of cells or morphological/functional state. Gene name in *italics* indicates mRNA expression; gene name in regular font indicates protein expression. pFTY720 indicates phosphorylated FTY720.

Refe-rence	Com-pound	Cell type	Effect	Model	Species
[61]	pFTY720	Total brain	• ↑ BDNF	Experimental Autoimmune Encephalomyelitis (EAE)	Mouse
[42]	pFTY720	Microglia	• ↓ IBA1+ cells, IBA1-CD18/32+ cells<br>• ↑ IBA1-CD206+ cells<br>• ↓ CD16, iNOS<br>• ↑ ARG1, CD206	Photothrombotic stroke	Mouse
[42]	pFTY720	Microglia	• ↓ CD16, iNOS<br>• ↑ ARG1, CD206<br>• ↑ M1 microglia morphology	Photothrombotic stroke derived primary cultures (LPS + IFN-y-stimulated)	Mouse
[43]	pFTY720	Microglia	• ↓ IBA1+ cells, CD68+ cells<br>• ↑ IBA1-CD206+ cells<br>• ↓ CD68	White matter ischemia	Mouse
[43]	pFTY720	Microglia	• ↓ *Cd86, iNos, Tnfα, Cd16/32, Il-1β*<br>• ↑ *Tgf-β, Ym, Arg1, Cd206*<br>• ↓ iNOS, CD16/32,<br>• ↑ STAT3, pSTAT3, CD206, ARG1	White matter ischemia derived primary cultures	Mouse
[44]	pFTY720 S1P	Microglia	• ↓ pP65<br>• ↓ *Tnfα, IL-1β, IL-6*<br>• ↑ *Arg1, Cd206, Il-4*	Primary cultures (Spn2 -/- ; LPS-stimulated)	Mouse
[45]	pFTY720	Microglia	• ↓ IBA1+ cells	Familial Alzheimer's disease (FAD)	Mouse
[46]	pFTY720	Microglia	• ↓ IBA1+ cells	Irradiation	Mouse
[47]	pFTY720	Microglia	• ↓ IBA1+ cells<br>• ↓ TNFα	Intracerebral hemorrhage (ICH)	Mouse
[48]	pFTY720	Microglia	• ↓ IBA1+ cells<br>• ↓ *Tnfα, Il-1β, Cxcl3*	Cuprizone-induced demyelination	Mouse
[49]	pFTY720	Microglia	• ↓ Sialoadhesin (SN)+ cells	Neuronal Ceroid Lipofuscinoses (CLN)	Mouse
[50]	pFTY720	Microglia	• ↓ MAC-3+ cells	EAE	Mouse
[51]	pFTY720	Microglia	• ↓ IBA1+ cells	FAD	Mouse

[53]	pFTY720	Microglia	• ↓ MAC-1+ cells, IBA1+ cells	Middle cerebral artery occlusion (MCAO)	Mouse
[54]	pFTY720	Microglia	• ↓ IBA1+ cells<br>• ↓ IL-1β, TNFα	Status Epilepticus (SE)	Rat
[55]	pFTY720	Microglia	• ↓ iNOS, Pre-IL-1β, COX2, CD11b, NLRP3<br>• ↑ IGF-1a, CD206<br>• ↓ *Cd86, Cox2, iNos, Il-1β, Il-6, Tnfα, Ifn-y*<br>• ↑ *Tgf-β1, Tgf-β2, Tgf-β3, Ccl2, Ccr2, Gcsf, Gm-csf, Igf*	Primary cultures (OGD-insulted)	Rat
[55]	pFTY720	Microglia	• ↓ IBA1+ cells<br>• ↓ iNOS, NLRP3<br>• ↑ TREM2<br>• ↓ TNFα, IL-1β (serum)	Ischemia	Rat
[56]	pFTY720	Microglia	• (during kindling) ↓ IBA1+ cells<br>• (during treatment) ↓ IBA1+ cells	Pentylenetetrazol (PTZ)-induced kindling	Mouse
[57]	pFTY720	Microglia	• No effect on pMAPK, pAKT, BCL2, BAX<br>• No effect on IL-12, TGF-β, TNFα<br>• ↑ ICAM	MOG-induced optic neuritis (MOG-ON)	Rat
[57]	pFTY720	Microglia	• ↓ ED+ cells	MOG-ON	Rat
[58]	pFTY720	Microglia	• ↓ CD68+ cells<br>• ↓ IL-6, IL-1β, TNFα	MPTP-induced Parkinson's disease (PD)	Mouse
[58]	pFTY720	Microglia	• ↓ IL-6, IL-1β, TNFα<br>• ↓ phosphorylation PI3K/AKT/GSK-3β<br>• ↓ ROS pP65, NLRP3 and CASP1	BV2 cell line (MPP+ stimulated) / Primary cultures	Rat
[59]	pFTY720	Microglia	• ↓ p38 MAPK<br>• No effect on total JNK or pJNK	Primary cultures (LPS-stimulated)	Rat
[59]	pFTY720	Microglia	• ↓ IBA1+ cells	Kainic acid seizure	Rat
[60]	pFTY720	Microglia	• ↓ CD86+ cells	EAE	Rat
[61]	pFTY720	Microglia	• ↓ FcGR-IV+ cells	EAE	Mouse
[62]	FTY720-Mitoxy	Microglia	• ↓ IBA1+	CNP-aSyn Tg animals	Mouse
[63]	pFTY720	Microglia	• ↓ IBA1+ cells↓ CD68+ cells	N9 cell line (LPS-stimulated)	Mouse
[64]	pFTY720	Microglia	• ↓ IBA1+ area	Krabbe's disease	Mouse
[65]	pFTY720	Microglia	• No effect on *Nos2, Ccl2, Il-1β, Tnf-α, Il-10*<br>• ↑ *Il-6, Arg1*	Primary cultures	Murine
[65]	pFTY720	Microglia	• No effect on *NOS2, CCL2, IL-1β, TNF-α, IL-10*<br>• ↑ *IL-6, ARG1*	Primary cultures	Human
[65]	pFTY720	Microglia	• ↓ *Ccl7, Cxcl13, Ccl5, Axl, Ccr2, Fosb, Ccl1, Fos, Cxcl11, H2-Aa, TNFα, Ccl17, Ccl4, H2-Ab1, Ccl2, Cxcl9*<br>• ↑ *Csf2, Chi3l3, IL-10, Igf1, Retnla, Cd206*	EAE in non-obese diabetes	Mouse
[66]	pFTY720	Microglia	• ↑ IBA1+ cells mediated via S1P1/S1P5	Lysolecithin-induceddemyelination	Mouse
[67]	pFTY720	Microglia	• No effect on IBA1+ cell area	Cuprizone-induced demyelination	Mouse
[68]	pFTY720	Microglia	• No effect on IBA1+ cell number and area	Cuprizone-induced demyelination	Mouse

[69]	pFTY720	Microglia	• No effect on MAC3+ cells	Cuprizone-induced demyelination	Mouse
[70]	pFTY720	Microglia	• No effect on IBA1+ cells	Facial nerve lesion	Mouse
[71]	(p)FTY720	Microglia	• ↓ MHC-II+ cells<br>• ↓ [125I]DPA-713 (TSPO-ligand)	fDTH-EAE (experimental allergic encephalomyelitis)	Rat
[71]	(p)FTY720	Microglia	• ↓ MHC-II+ cells<br>• ↓ [125I]DPA-713 (TSPO-ligand)	MOG-induced EAE	Rat
[72]	pFTY720	Microglia	• ↓ *Tnfα, Il-1β, Il-6*<br>• ↓ TNFα<br>• ↑ *Bdnf, Gdnf*	Primary cultures	Mouse
[73]	pFTY720	Microglia	• ↓ CXCL5	Primary cultures (LPS-stimulated)	Mouse
[74]	pFTY720	Microglia	• Modulation of genes with a STAT1 and IRF8 promotor binding motif<br>• ↓ *Il-1α, Il-1β, Ccl2*<br>• ↓ *Ccl2, Ccl3, Ccl4, Ccl8, Ccl9, Ccl12, Ccl22, Cxcl3, Cxcl9, Cxcl10, Cxcl11, Cxcl16, Il-1α, Il-6, Il-12b, Il-15, Il-18bp, Il-18, Il-19, Il-23a, Il-27, Il-1rn, Il-12rg, Il-10ra, Il-13ra1, Il-15ra, Tnfα, Tnfaip3, Tnfsf10, Tnfsf15, Tnfrsf1a, Tnfrsf1b, Tnfrsf14*<br>• ↓ *Cmpk2, Dhx58, Gbp2, Gbp3, Gbp4, Gbp5, Gbp6, Gbp7, Gbp10, Gbp11, Gbp2b, Isg20, Ifi35, Ifi44, Ifi44i, Ifi47, Ifi203, Ifi204, Ifi205, Ifih1, Ifnb1, Irg1, Irgm1, Irgm2, Ifit1, Ifit2, Ifit3, Ifitibi1, Ifitibi2, Ifit3b, Ifitm3, Mx1, Mx2, Oas2, Oas3, Oas12, Oasl1a, Oasl1b, Oasl1c, Oasl1g, Rsad2, Usp18, Usp21, Zbp1*<br>• ↓ CCL4, IL-1β, TNFα	Primary cultures (LPS+IFN-y stimulated)	Mouse
[75]	pFTY720	Microglia	• ↑ IL-16	Traumatic brain injury (TBI)	Mouse
[77]	pFTY720	Microglia	• ↑ monomeric ASC and ASC dimerization/oligomerization<br>• ↑ IL-1β and clCASP1 p20 subunit	Primary cultures (wildtype and ASC-/-)	Mouse
[80]	FTY720	Microglia	• ↑ Apoptosis independent of S1P receptor binding via ↑ SREBP2<br>• ↑ clCASP7<br>• ↑ clCASP9<br>• ↑*GSTTP1, RPL7P26, INSIG1, HSD17B7P2, HMGCS1, EYS, RNU4-2, CYP4Z2P, DDIT4, CCL4, LDLR, SC4MOL, PRAMEF18, LPIN1, CASZ1, HMGCR, OR56BB4, HSD3B1, BHLHE40, FBXW10, OR51F1, MVK, METT5DD1,CCNG2, C1orf180, AGR2, ANKRD30A, TSC22D3*	HMO6 cell line	Human
[80]	pFTY720	Microglia	• No effect on apoptosis	HMO6 cell line	Human
[81]	pFTY720	Microglia	• ↓ Neurotoxicity	Primary microglia neuron co-culture (Aβ stimulated)	Rat
[45]	pFTY720	Astrocyte	• ↓ GFAP	FAD	Mouse

[46]	pFTY720	Astrocyte	• No effect on GFAP+ cells	Irradiation	Mouse
[47]	pFTY720	Astrocyte	• ↓ GFAP<br>• ↓ TNFα	ICH	Mouse
[48]	pFTY720	Astrocyte	• ↓ GFAP+ cells<br>• ↓ *Tnfα, Il-1β, Cxcl3*	Cuprizone-induced demyelination	Mouse
[50]	pFTY720	Astrocyte	• ↓ GFAP+ cells<br>• ↑ *Slc1a3, Slc1a2*<br>• No effect on SLC1A3, SLC1A2	EAE	Mouse
[50]	pFTY720	Astrocyte	• ↓ *Slc1a3, Slc1a2*<br>• ↓ SLC1A2	Primary cultures	Mouse
[51]	pFTY720	Astrocyte	• ↓ GFAP+ cells<br>• ↓ Taurine/Cr	FAD	Mouse
[54]	pFTY720	Astrocyte	• ↓ GFAP+ cells<br>• ↓ IL-1β, TNFα	SE	Rat
[56]	pFTY720	Astrocyte	• (during kindling) ↓ GFAP+ cells<br>• (during treatment) ↓ GFAP+ cells	PTZ-induced kindling	Mouse
[61]	pFTY720	Astrocyte	• ↓ GFAP+ cells	EAE	Mouse
[64]	pFTY720	Astrocyte	• No effect on GFAP<br>• ↑ VIM in cerebellum	Krabbe's disease	Mouse
[65]	pFTY720	Astrocyte	• ↓ *Ccl2, Nos2, Csf2, Il-6, Tnfα, Il-10*<br>• ↓ CCL2, GM-CSF, TNFA, IL-6, NO<br>• ↓ nuclear P65	Primary cultures (LPS-stimulated)	Murine
[65]	pFTY720	Astrocyte	• ↓ *CCL2, NOS2, IL-6, IL-12A, IL-23A, IL-10*<br>• ↓ *NRTN, ARTN, BDNF, NTF4, GDNF, IL-11, LIF, CSPG4, NGF, HBEGF*<br>• ↑ *CSPG5, NTF3, PSPN,CNTF*	Fetal cells cultures (LPS-stimulated)	Human
[65]	pFTY720	Astrocyte	• ↓ *Nos2, Il-6, Ccl20, Ccl2, Ccl1, Ifn-y, Csf2, Il23a, Il21, Il21a, Ccl17, Cxcl10, Il-1β, Tnfα, H2-Aa, H2-Ab1*<br>• ↑ *Cxcl12, Il-33*	EAE in non-obese diabetes	Mouse
[66]	pFTY720	Astrocyte	• ↑ GFAP area via S1P3/S1P5	Lysolecithin-induced demyelination	Mouse
[67]	pFTY720	Astrocyte	• No effect on GFAP+ cells	Cuprizone-induced demyelination	Mouse
[68]	pFTY720	Astrocyte	• No effect on GFAP+ cell number and area	Cuprizone-induced demyelination	Mouse
[69]	pFTY720	Astrocyte	• No effect on GFAP+ cells	Cuprizone-induced demyelination	Mouse
[70]	pFTY720	Astrocyte	• No effect on GFAP+ cells	Facial nerve lesion	Mouse
[73]	pFTY720	Astrocyte	• ↓ CXCL5<br>• ↓ *Cxcl10, Ccl2*	Primary cultures (LPS-stimulated)	Mouse
[82]	pFTY720	Astrocyte	• ↓ GFAP+ cells	EAE	Mouse
[83]	pFTY720	Astrocyte	• ↑ Ca^{2+} signals<br>• ↓ cAMP formation<br>• ↑ Inositol phosphate (IP) formation<br>• ↑ Cell migration	Primary cultures	Rat
[84]	pFTY720	Astrocyte	• ↓ GFAP+ cells, area and intensity<br>• ↑ IKBα<br>• ↓ *Tnfα, iNos*	Huntington's disease (HD)	Mouse
[85]	(p)FTY720	Astrocyte	• ↓ Astrocytic activation<br>• ↓ Aβ	Infection	Mouse

[86]	pFTY720	Astrocyte	• ↓ GFAP+ area	Stroke	Mouse
[87]	pFTY720	Astrocyte	• ↓ Fetal IL-6, CASP3, S100β	Endotoxin stimulation (Material inflammation) + Maternal treatment with FTY720	Rat
[88]	pFTY720	Astrocyte	• ↓ GFAP+ cells	Human induced pluripotent stem cell transplantation into wildtype animals	Human
[89]	pFTY720	Astrocyte	• ↑ GM-CSF	Immortalized cells	Human
[90]	(p)FTY72 0	Astrocyte	• ↑ NO<br>• ↓ pNFκB/P65	Complex regional pain syndrome (CRPS)	Mouse
[90]	(p)FTY72 0	Astrocyte	• ↑ Astrocytic activation	CRPS	Mouse
[91]	pFTY720	Astrocyte	• ↓ pATF-2, pcJUN, pJNK, pMEK1, p70S6K, pAKT, pGSK3β, PTEN, IL-6, MIP-1β	Mucolipidosis (Mcoln1-/- animals)	Mouse
[92]	pFTY720	Astrocyte	• ↓ Nuclear NFκB<br>• ↓ NO production	Primary cultures	Human
[92]	pFTY720	Astrocyte	• ↓ IL1R-GFAP+ cells, IL17RA-GFAP+ cells, S1P1-GFAP+ cells, S1P3-GFAP+ cells, iNOS-GFAP+ cells, Nitrotyrosine-GFAP+ cells	EAE	Mouse
[93]	pFTY720	Astrocyte	• ↓ cell death (MTT assay)<br>• ↓ HMGB1,TNFα<br>• ↓ TLR2, pPI3K and nuclear NFκB	Primary cultures (Oxygen-glucose deprivation OGD-induced)	Rat
[94]	pFTY720	Astrocyte	• ↑ Viability<br>• ↓ LDH release, TUNEL+ cells<br>• ↓ IL-1β, TNF-α, IL-6, ICAM-1, VCAM-1 and CXCL-10	Primary cultures (OGD-induced)	Rat
[95]	pFTY720	Astrocyte	• ↑ *LIF, IL-11 and HBEGF*	Primary cultures (TNFα-stimulated)	Human
[95]	pFTY720	Astrocyte	• ↑ *LIF, IL-11 and HBEGF*<br>• ↑ LIF, IL-11 and HBEGF<br>• ↓ CXCL10, BAFF, MX1, and OAS2	Astrocytoma cell line (TNFα-stimulated)	Human
[96]	pFTY720	Astrocyte	• ↑ Ki67+ cells<br>• No effect on *Il-1β, Ccl2, Ccl20, Cxcl12,* Igf1, Cntf, *S1p1, S1p3*<br>• ↑ *Gdnf*	Primary cultures (TNFα/LPS-stimulated)	Mouse
[97]	pFTY720	Astrocyte	• ↓ Immediate-early astrocytes	EAE	Mouse
[98]	pFTY720<br>FTY720	Astrocyte	• ↑ ADR-β2<br>• ↑ *Adr-β2*<br>• ↓ MHC-II via ↓ Nuclear NFκB P65<br>• ↓ Mobility	Primary cultures (IFN-y-stimulated)	Rat
[99]	(p)FTY72 0	Astrocyte	• ↓ NLRP3, IL-1β, via IL-10	Wildtype and S1PR1-astrocyte knock-out animals	Mouse
[103]	pFTY720	Astrocyte	• ↓ *ASM*<br>• ↓ Ceramide levels	Primary cultures (TNFα-stimulated)	Human
[104]	pFTY720	Astrocyte	• ↑ Translocation of S1P1R to trans-Golgi network	Primary cultures	Rat

			• ↓ Forskolin-induced cAMP levels		
[107]	pFTY720	Astrocyte	• ↑ pERK-GFAP+ cells, pP38-GFAP+ cells, pCREB-GFAP+ cells	Mixed glial cultures	Rat
[120]	pFTY720	Astrocyte	• ↑ pERK (co-localized with GFAP)	Primary cultures	Rat
[42]	pFTY720	Neuron	• ↓ Neuronal loss (Nissl staining)	PT stroke	Mouse
[43]	pFTY720	Neuron	• No effect on neuronal loss (Nissl staining)<br>• ↑ panNfacs/CASPR (Ranvier's nodes)<br>• ↑ Length Nav1.6 domains	White matter ischemia	Mouse
[46]	pFTY720	Neuron	• ↓ PI+ cells<br>• No effect on DCX+ or βIII-TUB+ cells	Primary cultures (irradiated)	Mouse
[46]	pFTY720	Neuron	• No effect on DCX+ cells	Wildtype animals	Mouse
[46]	pFTY720	Neuron	• ↑ DCX+ cells	Irradiation	Mouse
[47]	pFTY720	Neuron	• ↑ Surviving neurons (Cresyl Violet staining)	ICH	Mouse
[49]	pFTY720	Neuron	• ↓ Retinal thinning<br>• ↓ SMI32+ axonal spheroids<br>• ↑ Cresyl Violet+ cells	CLN	Mouse
[50]	pFTY720	Neuron	• No effect on neurite growth	PC-12 cell line (stimulated with IL-β+TNFα stimulated astrocyte medium)	Mouse
[50]	pFTY720	Neuron	• ↑ Number of axons	EAE	Mouse
[54]	pFTY720	Neuron	• ↓ Mossy fiber sprouting (Timm staining)<br>• ↑ NeuN+ cells<br>• ↓ FJB+ (Fluoro-Jade B) cells	SE	Rat
[56]	pFTY720	Neuron	• (during kindling) ↑ Nissl+ cells, NeuN+ cells<br>• (during treatment) ↑ Nissl+ cells, NeuN+ cells	PTZ-induced kindling model	Mouse
[57]	pFTY720	Neuron (retinal)	• No effect on apoptosis of RGCs	Primary cultures	Rat
[57]	pFTY720	Neuron (retinal)	• ↓ β-APP+ particle cells<br>• No effect on apoptosis RGCs	MOG-ON	Rat
[58]	pFTY720	Neuron	• ↓ PI+ cells	SH-SY5Y cell line (treated with MPP+ BV-2 cell medium)	Human
[58]	pFTY720	Neuron	• ↑ TH+ cells	MPTP-induced Parkinson's disease (PD)	Mouse
[59]	pFTY720	Neuron	• ↓ Fluoro-Jade C<br>• ↑ NeuN+ cells<br>• ↑ Nissl staining<br>• ↓ LDH release<br>• ↓ PI+ cells	Kainic acid seizure	Rat
[59]	pFTY720	Neuron	• ↓ LDH release	Primary cultures (NDMA-stimulated)	Rat
[60]	pFTY720	Neuron	• Normalized electrophysiological responses (sensory evoked potential/ visual evoked potential)<br>• ↑ Bielschowsky silver impregnation	EAE	Rat
[61]	pFTY720	Neuron	• ↑ *Bdnf*	EAE	Mouse

[64]	pFTY720	Neuron	• No effect on Calbindin+ Purkinje cells<br>• Structural changes in Purkinje cell layers<br>• No effect on SMI32+ cells	Krabbe's disease	Mouse
[65]	pFTY720	Neuron	• ↓ Axonal loss	EAE in non-obese diabetes	Mouse
[67]	pFTY720	Neuron	• No effect on β-APP+ particle cells	Cuprizone-induced demyelination	Mouse
[68]	pFTY720	Neuron	• ↓ APP-NF-H+ cells	Cuprizone-induced demyelination	Mouse
[69]	pFTY720	Neuron	• No effect on APP+ cells<br>• No effect on NFL+ cells	Cuprizone-induced demyelination	Mouse
[70]	pFTY720	Neuron	• ↑ *cFos, FosB, Egr1, Egr2, Acta, Tmp1a, Cnn1, Tagln, Bdnf, Ccl2,*<br>• ↓ *Ccl3, Ccl9*<br>• ↑ Growth cone area and neurite length<br>• ↑ cFOS, FOSB, EGR1 and EGR2<br>• ↑ cFOS-NeuN+ cells, DCX+ cells<br>• No effect on SMI-32+ cells<br>• Modulation of G12/13 G protein-RhoA-MRTF-A/SRF signaling	Primary cultures / DRG neurons / organotypic slice cultures	Mouse
[81]	pFTY720	Neuron	• ↓ Neuronal cell death	Primary cultures (Aβ-stimulated) microglia neuron co-culture	Rat
[82]	pFTY720	Neuron	• ↑ Neurofilament Heavy chain (NF-H)	EAE	Mouse
[84]	pFTY720	Neuron	• ↓Dendritic spine loss<br>• ↑ PSD95	HD	Mouse
[88]	pFTY720	Neuron	• No effect on NeuN+ cells	Human induced pluripotent stem cell transplantation into wildtype animals	Human
[92]	pFTY720	Neuron	• ↑ βIII-TUB<br>• ↓ TUNEL+ cells	Primary cultures	Rat
[105]	pFTY720	Neuron	• ↑ MTT cell viability	Primary cultures (Glutamate/NDMA-stimulated)	Mouse
[105]	pFTY720	Neuron	• ↓ Intraneuronal Ca(2+)	B6.thy1-TN-XXLtransgenic Ca(2+) reporter animals	Mouse
[106]	pFTY720	Neuron	• ↑ BrdU-DCX+ cells	Kainic acid induced seizure	Rat
[106]	pFTY720	Neuron	• ↑ βIII-TUB+ cells	Primary cultures	Rat
[107]	pFTY720	Neuron	• ↑ pERK, pP38, pCREB	Co-culture human fetal oligodendrocyte precursor cells (OPCs) with rat (dorsal root ganglion) DRG neurons	Human /rat
[43]	pFTY720	Oligodendro cyte	• ↓ TUNEL+/Olig2+ cells<br>• ↑ MBP+/Olig2+ cells	White matter ischemia derived primary cultures (microglia medium treated)	Mouse

[48]	pFTY720	Oligodendrocyte	• ↓ TUNEL+ cells<br>• ↑ CNPase<br>• ↑ *Mag*, *Cnp*	Cuprizone-induced demyelination	Mouse
[50]	pFTY720	Oligodendrocyte	• ↓ Demyelination	EAE	Mouse
[56]	pFTY720	Oligodendrocyte	• (during kindling) ↑ Luxol fast blue (LFB) intensity<br>• (during kindling) ↑ MBP+ cells, NG2+ cells<br>• (after kindling) ↑ LFB intensity<br>• (after kindling) ↑ MBP+ cells, NG2+ cells	PTZ-induced kindling	Mouse
[57]	pFTY720	Oligodendrocyte	• ↑ LFB intensity	MOG-ON	Rat
[60]	pFTY720	Oligodendrocyte	• ↑ LFB staining	EAE	Rat
[64]	pFTY720	Oligodendrocyte	• ↑ MBP, OLIG2<br>• No effect on MOG<br>• No effect on levels of myelin debris	Krabbe's disease	Mouse
[65]	pFTY720	Oligodendrocyte	• ↓ Demyelination	EAE in non-obese diabetes	Mouse
[66]	pFTY720	Oligodendrocyte	• ↑ MBP+ staining<br>• ↑ NOGO-A+ or PDGFαR+ extensions	Lysolecithin-induced demyelination	Mouse
[67]	pFTY720	Oligodendrocyte	• ↑ NOGO-A+ cells<br>• No effect on remyelination or number of mature oligodendrocytes (MBP, PLP1)	Cuprizone-induced demyelination	Mouse
[68]	pFTY720	Oligodendrocyte	• (acute Cuprizone) ↑ Remyelination<br>• (chronic Cuprizone) No effect on remyelination	Cuprizone-induced demyelination	Mouse
[69]	pFTY720	Oligodendrocyte	• No effect on LFB staining<br>• No effect on PLP1+ cells<br>• No effect on NOGO-A+ cells<br>• No effect on PLP1,MBP, MAG, MOG, OMG, MYEF2, MOBP, MYT1L	Cuprizone-induced demyelination	Mouse
[82]	pFTY720	Oligodendrocyte	• ↑ Fluoromyelin	EAE	Mouse
[88]	pFTY720	Oligodendrocyte	• ↑ OLIG2+ cells<br>• ↑ Myelination (PLP1)	Human induced pluripotent stem cell transplantation into wildtype animals	Human
[106]	pFTY720	Oligodendrocyte	• ↑ BrdU-NG2+ cells in corpus callosum	Kainic acid induced seizure	Rat
[107]	pFTY720	Oligodendrocyte	• ↑ MBP+ cells, O1+ cells<br>• ↑ Axonal myelin ensheathment<br>• ↑ O4-Ki67+ cells, O4-GC+ cells<br>• ↓ TUNEL+ cells<br>• ↑ pP38, pERK, pCREB	Co-culture human fetal OPCs with rat DRG neurons	Human /rat
[110]	pFTY720	Oligodendrocyte	• ↑ MBP+ cells<br>• ↑ pAKT, pERK	Primary cultures (CoCl2-stimulated)	Rat
[110]	pFTY720	Oligodendrocyte	• ↓ Demyelination (Klüver-Barrera staining)<br>• ↑ MBP intensity	Subcortical ischemic vascular dementia	Mouse

			• ↑ GSTpi-BrDU+ cells<br>• No effect on OLIG2-BrdU+ cells		
[111]	pFTY720	Oligodendro cyte	• ↑ LFB staining, PLP1 staining<br>• ↑ Remyelinated axons<br>• ↑ OLIG2+ cells, OLIG2-BrdU+ cells	Lysolecithin-induced demyelination	Mouse
[114]	pFTY720<br>FTY720	Oligodendro cyte	• ↑ pERK1/2, pAKT<br>• ↓ TUNEL+ cells after growth factor depletion<br>• ↑ OPC differentiation	Primary cultures	Rat
[115]	pFTY720 + S18	Oligodendro cyte	• ↑ OLIG2+ cells	Neural precursor cells derived from embryonic stem cells	Mouse
[116]	pFTY720	Oligodendro cyte	• ↓ A2B5+ cell process extensions<br>• ↓ GALC+ cells<br>• ↓ TUNEL-A2B5+ cells after growth factor depletion<br>• ↑ pERK1/2	Fetal cell cultures	Human
[117]	pFTY720	Oligodendro cyte	• ↑ MBP, CNPase+ cells<br>• ↑ NG2+ cells, NG2-BrdU+ cells, CNPase-BrdU+ cells<br>• ↑ Sonic Hedgehog, Smoothened, GLI1	EAE	Mouse
[118]	pFTY720	Oligodendro cyte	• ↓ Ceramide	EAE	Rat
[118]	pFTY720	Oligodendro cyte	• ↓ Ceramide	HOG cell line (TNFα/IFNγ-stimulated)	Human
[119]	pFTY720	Oligodendro cyte	• ↑ MBP+ cells<br>• ↑ MOG	EAE (organotypic cerebellar slices stimulated with 2D2 transgenic mouse or MOG-splenocytes)	Mouse
[121]	pFTY720	Oligodendro cyte	• ↑ *Bdnf*<br>• ↑ BDNF<br>• No effect on *Ngf*<br>• ↑ Histone 3 acetylation (AcH3)	OLN-93 cell line (α-synuclein-treated)	Rat

Supplementary table 2. Molecular effects of Dimethyl Fumarate (DMF) and Monomethyl Fumarate (MMF) on microglia, astrocytes, neurons and oligodendrocytes. ↑ indicates increased level of expression, number of cells or morphological/functional state; ↓ indicates reduced expression level, number of cells or morphological/functional state. Gene name in *italics* indicates mRNA expression; gene name in regular font indicates protein expression.

Refe-rence	Com-pound	Cell type	Effect	Model	Species
[123]	DMF	Total brain	• ↓ MCP1, KC, VEGF<br>• No effect on IL-1β, IL-6 and MIP1α	Hypo perfusion	Mouse
[125]	DMF	Total brain	• ↑ *Nqo-1, Ho-1, Sod2, Gpx, Nrf2, Il-10*<br>• ↓ *Il-1β, iNos,*	Cerebral hypoxic Ischemia in wildtype and Nrf2-/- animals	Mouse
[127]	DMF	Total brain	• ↑ NRF-2, HO-1, MnSOD, IκBα<br>• ↓ nNOS, NFκB, IL-1β, CD11b, COX-2	MPTP Parkinson's disease (PD) model	Mouse
[128]	DMF	Total brain	• ↓ *Nqo-1*	Rumpshaker hypomyelination	Mouse

[129]	MMF<br>DMF	Total brain	• ↑ BDNF, GDNF, NT3, IκBα, BCL2<br>• ↓ TNFα, IL-1β, COX2, iNOS, NO, AIF, FAS ligand<br>• ↑ GPX1, MnSOD, HO-1, NRF-2, GSH<br>• ↓ Malondialdehyde	Spinal cord injury (SCI) model	Mouse
[131]	DMF	Total brain	• ↑ pGSK-3β, pCRMP2<br>• ↑ *Nqo-1, Osgin1*	Wildtype animals	Mouse
[132]	DMF	Total brain	• ↓ *Tnfα, Il-1β, Ccl2, Il-1α, C1q*	Cxcr1-GFP (LPS-stimulated)	Mouse
[140]	DMF	Total brain	• ↑ *Ho-1, Cd36, Catalase, Nqo1, Nrf2, Hp, Cd163, Il-10*<br>• ↓ *iNos, Il-1β*	Intracerebral hemorrhage (ICH)	Mouse/ Rat
[141]	DMF	Microglia / astrocyte	• ↓ Tnfα, I-1β, Il-6, *iNos*<br>• ↓ NO<br>• ↑ *Nqo-1*<br>• ↑ NQO1, GSH	Co-culture primary cultures (LPS-stimulated)	Rat
[122]	DMF	Microglia	• ↓ *TNFα, IL-6, IL-10, MIR-155*<br>• ↑ *HMOX1*	Adult cell cultures (LPS-stimulated)	Human
[122]	MMF	Microglia	• No effect on microglia phenotype	Adult cell cultures (LPS-stimulated)	Human
[122]	DMF	Microglia	• ↓ TNFα, IL-6, IL-10, MIR-155<br>• ↑ HMOX1	Fetal cell cultures (LPS-stimulated)	Human
[122]	MMF	Microglia	• No effect on microglia phenotype	Fetal cell cultures (LPS-stimulated)	Human
[123]	DMF	Microglia	• ↓ IBA1+ area	Hypo perfusion	Mouse
[124]	MMF<br>DMF	Microglia	• (DMF) ↓ CD68+ cells, iNOS<br>• ↓ Pro-inflammatory cytokines	Oxygen-glucose deprivation (OGD)	Rat
[124]	DMF<br>MMF	Microglia	• (MMF) ↓ *Il-12b, Ifn-y, Il-17, Gm-csf, Mip-2*	Mixed glia/neuron cultures	Rat
[124]	DMF<br>MMF	Microglia	• (DMF) ↑ NRF-2, HO-1<br>• (DMF) ↓ *Il-17, Rantes, Eotaxin, Il-18, Il-1β*	Primary cultures (OGD-treated)	Rat
[125]	DMF	Microglia	• (24h and 6h) ↓ IBA1+ area	Cerebral hypoxic Ischemia model in wildtype and Nrf2-/- animals	Mouse
[126]	DMF	Microglia	• ↓ MAC-3+ cells	EAE	Mouse
[127]	DMF	Microglia	• ↓ IBA1	MPTP PD model	Mouse
[128]	DMF	Microglia	• No effect on IBA1+ cells<br>• ↓ CD68+ cells	Rumpshaker hypo myelination	Mouse
[129]	DMF<br>MMF	Microglia	• ↓ IBA1+ cells<br>• ↑ GDNF, BDNF, NT3	SCI	Mouse
[130]	DMF	Microglia	• ↓ OX-42+ cells	ICH	Mouse
[131]	DMF	Microglia	• ↓ IBA1+ cells	Nrf2+/+	Mouse
[132]	DMF	Microglia	• ↓ IBA1+ cells (high dose)<br>• ↓ *Tnfα, Il-1β, Nos2, Il-23a, Il-2b*<br>• ↓ NFκB (P65), pIKKα/β	Nrf2-/- and wildtype primary cultures (LPS-stimulated)	Mouse
[132]	DMF	Microglia	• ↓ CXCR1-GFP+ cells<br>• ↓ IBA1+ cells	Cxcr1-GFP (LPS-stimulated) animals	Mouse
[133]	DMF	Microglia	• (Aged rats) ↑ CD68+	Streptozotocin-induced AD model	Rat
[134]	DMF	Microglia	• ↓ *P2y6, P2y12*	Primary cultures	Mouse

			• (ATP-stimulated)<br>• ↓ number of microglia cells migrating towards an ATP gradient<br>• (ATP-stimulated) ↓ [Ca2+]i<br>• (ATP-stimulated) ↑ mHFt uptake, *Tim2*<br>• (LPS-stimulated) ↓ phagocytosis after LPS<br>• ↑ *Ym1, Arg1, Fizz*<br>• (LPS+IFNy-stimulated) ↓ *Il-1β, Tnfα, Inos*<br>• (LPS+IFNy-stimulated) ↑ Arg1, *Mtfr1, Tim2*		
[134]	DMF	Microglia	• ↓ microglia motility<br>• No effect on number of microglia	Cxcr3-GFP hippocampal slices (ATP-stimulated)	Mouse
[135]	DMF	Microglia	• ↑ *Igf1, iNos, Tgf-1β, Tnfα, Mrc1*<br>• No effect on phagocytosis<br>• No effect on IGF1	Primary cultures (LPS/ IL-4-stimulated)	Rat
[135]	MMF	Microglia	• No effect on *Igf1, iNos, Tgf-1β, Tnfα, Mrc1*<br>• No effect on phagocytosis<br>• No effect on IGF1	Primary cultures (LPS/IL-4-stimulated)	Rat
[136]	MMF<br>DMF	Microglia	• (MMF) ↓ Cell survival<br>• (MMF) ↓ IL-6, TGFb, TNFα, CD11b, CD68	Isolated microglia cultures from GBM tumors	Mouse
[137]	MMF	Microglia	• ↓ *Tnf, Il-1β, Sp1, Hmox1, Nos2*<br>• ↑ *Cx3cr1, Cd200r, Nr4a2, Igf1, Arg1, Rtnla, Mrc1, Lgals3, Trem2*<br>• ↑ Phagocytosis<br>• ↑ [Ca2+]i<br>• ↓ acetylation (of NfkB P65<br>• ↑ pAMPK	N9 cell line (LPS-stimulated)	Mouse
[137]	MMF	Microglia	• ↓ *Il-1β*<br>• ↑ *Arg1, Retnla, Mrc1, Lgal3*	EAE	Mouse
[138]	DMF<br>MMF	Microglia	• No effect on MAC-3+ cells	Cuprizone-induced demyelination	Mouse
[138]	DMF<br>MMF	Microglia	• (DMF) ↓ NO bursts	Primary cultures	Mouse
[139]	DMF	Microglia	• No effect on MAC3-3+ cells<br>• (DMF+IFN-β) ↓ MAC-3+ cells	EAE	Mouse
[140]	DMF	Microglia	• ↑ RBC phagocytic activity	Primary cultures	Rat
[142]	DMF	Microglia	• ↓ *iNos, Tnfα, Il-1β, Il-6*<br>• ↓ pERK<br>• ↑ NRF-2	Primary cultures (LPS-stimulated)	Rat
[143]	DMF<br>MMF	Microglia	• (DMF) ↓*NfkB*-related genes,<br>• (MMF) ↑ *NfkB-related genes,*<br>• (DMF) ↑ *Nrf2*-related genes<br>• (MMF) ↓ *Nrf2*-related genes<br>• (DMF) ↓ IL-6, MCP-1, TNFα, KC, Nitrite, Nitrate, IL-12p40(MMF) No effect on IL-6, MCP-1, TNFα, KC, Nitrite, Nitrate, IL-12p40	Primary cultures (LPS+IFN-y-treated)	Mouse
[144]	DMF	Microglia	• ↑ *Hmox1*<br>• ↑ ALAS1, BVR<br>• (LPS-stimulated) ↓ TNFα, PGE2, via HO-1 and Nrf2	BV-2 cell line	Rat
[145]	DMF	Microglia	• No effect on *Il-1β*	Cortical explants	Mouse

[145]	DMF	Microglia	• ↑ *Ho-1*<br>• ↓ NO<br>• ↓ Il-1β, *iNos*	BV-2 cell line (LPS-stimulated)	Rat
[146]	MMF	Microglia	• ↓ CXCL10	HMC3 cell line + monocyte co-culture (stimulated with HIV particles)	Human
[146]	MMF	Microglia	• ↓ CXCL10, CCL5<br>• No effect on CCL2, IL-6	Primary cultures	Human
[124]	DMF<br>MMF	Astrocyte	• (MMF) ↓ *Il-12b, Ifn-y, Il-17, Gm-csf, Mip-2*	Mixed glia/neuron cultures	Rat
[125]	DMF	Astrocyte	• (24h) ↑ GFAP+ cells<br>• (6h) ↓ GFAP+ cells<br>• ↑ Glutamine Synthetase (GS), AQP4	Cerebral hypoxic Ischemia	Mouse
[128]	DMF	Astrocyte	• No effect on GFAP+ cells (spinal cord)	Rumpshaker hypomyelination	Mouse
[129]	DMF<br>MMF	Astrocyte	• ↓ GFAP + cells<br>• ↑ GDNF, BDNF, NT3	SCI	Mouse
[131]	DMF	Astrocyte	• ↓ GFAP+ cells	Nrf2+/+ animals	Mouse
[132]	DMF	Astrocyte	• ↓ *Ggta1, H2-d1, Serping1*	Nrf2-/- and wildtype primary cultures (LPS-stimulated)	Mouse
[132]	DMF	Astrocyte	• ↓ GFAP+ cells	Cxcr1-GFP (LPS-treated) animals	Mouse
[136]	MMF<br>DMF	Astrocyte	• (DMF) ↓ GBM cell survival (proteasome inhibitor toxicity)	Glioblastoma tumor cultures	Mouse
[142]	DMF	Astrocyte	• ↓ *iNos, Tnfα, Il-1β, Il-6*	Primary cultures (LPS-stimulated)	Rat
[147]	DMF<br>MMF	Astrocyte	• No effect on *Ngf, Bdnf, Gdnf, Fgf2, Pdgfa, Cntf, Tnfα, Il-6, Il-1β, iNos*	Primary cultures (LPS- or IFN-y+IL-1β-stimulated)	Rat
[148]	DMF<br>MMF	Astrocyte	• (DMF) ↓ *Cxcl10*<br>• No effect on *Hmox1, Osgin1, Nqo-1*<br>• (DMF/MMF) ↓ intracellular ROS production<br>• (DMF) ↓ *mMiR-155, mIR-146*	Primary cultures (IL-1β-stimulated)	Mouse
[148]	DMF<br>MMF	Astrocyte	• (DMF) ↓ *Il-6, Cxcl10, Ccl2*<br>• No effect on *HMOX1, OSGIN1, NQO-1*<br>• (DMF) ↓ intracellular ROS production<br>• (DMF) ↓ *MiR-155*	Primary cultures (IL-1β-stimulated)	Human
[149]	DMF	Astrocyte	• ↑ *Ho-1, Gclm, Gclc, Nqo-1*	Primary cultures	Mouse
[150]	DMF	Astrocyte	• (4 hours) ↑ *Hdac*1,2,4<br>• (24h) ↓ HDAC1,2	Primary cultures (pro-inflammatory cytokine stimulated)	Rat
[151]	DMF	Astrocyte	• ↑ Placental alkaline phosphatase (PAP)	N18-RE-105 cell line	Rat/Mouse hybrid
[105]	DMF	Neuron	• ↓ [Ca2+]i	TN-XXL transgenic Ca2+ reporter animals	Mouse

[105]	DMF	Neuron	• ↓ Neurotoxicity	Primary cultures (Glutamate or NDMA-stimulated)	Mouse
[123]	DMF	Neuron	• Improved evoked compounds action potential (CAP)<br>• No effect on axonal refractoriness<br>• No effect on APP+ cells	Hypo perfusion	Mouse
[124]	DMF<br>MMF	Neuron	• (MMF) ↓ *Il-12b, Ifn-y, Il-17, Gm-csf, Mip-2*	Mixed glia/neuron cultures	Rat
[124]	DMF<br>MMF	Neuron	• (MMF) ↑ Cell survival<br>• (MMF) ↓ *Il-12b, Ifn-y, Il-17, Gm-csf, Mip-2*	Primary cultures (OGD-treated)	Rat
[125]	DMF	Neuron	• ↑ Cresyl violet + cells	Cerebral hypoxic Ischemia	Mouse
[126]	DMF	Neuron	• ↓ APP+ cells	Cuprizone-induced demyelination	Mouse
[127]	DMF	Neuron	• ↓ Neuronal cell loss<br>• ↓ α-synuclein (α SYN)+ neurons<br>• ↑ T, DAT, MAP2, NGF TH+ cells<br>• ↑ NRF-2-NeuN+ cells in striatum	MPTP PD model	Mouse
[127]	DMF	Neuron	• ↑ Cell viability<br>• ↓ iNOS<br>• ↑ MnSOD	SHSY5Y cell line (MPTP-treated)	Human
[130]	DMF	Neuron	• ↓ Evans dye extravastation	Intracerebral hemorrhage	Mouse
[131]	DMF	Neuron	• ↓ pTAU<br>• (DMF+IFN-β) ↑ SMI3I+ cells	Nrf2+/+ animals	Mouse
[132]	DMF	Neuron	• ↓ Cleaved CASP3+ cells<br>• ↑ Cell survival<br>• ↓ Membrane potential disruption	Nrf2-/- and wildtype primary cultures (treated with microglia medium)	Mouse
[133]	DMF	Neuron	• ↓ FJB+ cells<br>• ↓ Nitrotyrosine	AD model (streptozotocin-induced)	Rat
[137]	MMF	Neuron	• ↓ Frequency of glutamatergic sEPSCs, sEPSC half-width, sEPSC decay time	EAE	Mouse
[138]	DMF	Neuron	• No effect on APP+ cells	Cuprizone-induced demyelination	Mouse
[139]	DMF	Neuron	• ↓ Bielschowsky silver staining	EAE	Mouse
[143]	DMF	Neuron	• ↓ Oxygen consumption rate	Primary cultures (LPS+IFN-y microglia medium treated)	Rat
[146]	MMF	Neuron	• ↓ MAP2-PI+ cells	Fetal cell cultures (MMF treated microglia conditioned medium)	Human
[152]	DMF<br>MMF	Neuron	• Decreased neurotoxicity of macrophages by ↑ HO-1 expression	Fetal primary cultures (HIV- macrophage derived medium treated)	Human
[153]	DMF	Neuron	• ↑ NF, GAP43	Sciatic nerve damage	Mouse
[123]	DMF	Oligodendrocyte	• No effect on MBP+ intensity or MAG+ intensity	Hypo perfusion	Mouse
[126]	DMF	Oligodendrocyte	• ↑ LFB+ cells	EAE	Mouse

[126]	DMF	Oligodendrocyte	• ↑ OLIG2+ cells<br>• No effect on NOGO-A+ cells	Cuprizone-induced demyelination	Mouse
[128]	DMF	Oligodendrocyte	• No effect on MBP, PLP1<br>• No effect on protein folding response (CHOP+ cells)<br>• No effect on myelin thickness (G-ratio) in optic nerve	Rumpshaker hypomyelination	Mouse
[135]	DMF<br>MMF	Oligodendrocyte	• ↑ Proliferation<br>• No effect on GALC/A2B5 ratio	Primary cultures (+ microglia DMF/MMF supernatant)	Rat
[138]	DMF<br>MMF	Oligodendrocyte	• ↑ Luxol Fast Blue (LFB) signal in corpus callosum<br>• ↑ MOG, PLP1, MBP in corpus callosum<br>• No effect on NOGO-A and OLIG2+ cells	Cuprizone-induced demyelination	Mouse
[138]	DMF<br>MMF	Oligodendrocyte	• No effect on cell viability	CG4 cell line (H2O2 stimulated, SNP-stimulated)	Mouse
[139]	DMF	Oligodendrocyte	• No effect on LFB+ cells<br>• (DMF+IFN-β) ↑ LFB+ cells<br>• (DMF+IFN-β) ↑ CNPase+ cells	EAE	Mouse
[148]	DMF	Oligodendrocyte	• ↑ O4+ and NG2+ cells	Neural progenitor cell (NPC) cultures	Human
[148]	DMF	Oligodendrocyte	• ↑ O4+ and NG2+ cells	Neural progenitor cell (NPC) cultures	Mouse
[154]	DMF	Oligodendrocyte	• ↑ LFB+ cells via HCA_2	EAE	Mouse
[155]	DMF	Oligodendrocyte	• ↑ Myelination	Cuprizone-induced demyelination	Mouse
[156]	DMF	Oligodendrocyte	• (24 hours) ↑ Glutamine, Arginine<br>• (72 hours) ↓ Arginine<br>• (24/72 hours) ↑ Succinate, Fumarate, Malate, GSH<br>• (24 hours) ↑ PE, PG, PS<br>• (24 hours) ↓ PC, SM, FFA<br>• (72 hours) ↑ PC, PE, SM, PG, PI, PS<br>• (72 hours) ↓ PA	MO3.13 cell line	Human

Supplementary table 3. Molecular effects of Glatiramer Acetate (GA) on microglia, astrocytes, neurons and oligodendrocytes. ↑ indicates increased level of expression, number of cells or morphological/functional state; ↓ indicates reduced expression level, number of cells or morphological/functional state. Gene name in *italics* indicates mRNA expression; gene name in regular font indicates protein expression.

**Reference**	**Cell type**	**Effect**	**Model**	**Species**
[160]	Total brain	• ↑ BDNF, pAKT, pMAPK, BCL-2<br>• ↓ BAX	EAE	Rat
[162]	Total brain	• ↑ BDNF, IGF<br>• ↓ TNFα, IL-6	Cranial irradiation	Rat
[167]	Total brain	• ↓ Aβ plaques in hippocampus	Double-transgenic (APP/PS1) Alzheimer's disease (AD) model	Mouse
[175]	Total brain	• ↑ *Il-17a*, *H2-Ab1*, *Cxcl16*, *Tnfα*	EAE	Mouse

[178]	Total brain	• ↑ *Bdnf-1, Bdnf-4, Bdnf-IX*<br>• ↓ IL-4, IL-1	CAG140 KI and N171-82Q transgenic Huntington Disease (HD) models	Mouse
[185]	Lesional white matter	• No effect on glutamate, NAA, Cr and phosphocreatine	MS-patients	Human
[186]	Total tissue (spinal cord)	• ↑ *Bdnf, Igf1, Il-5*	Lysolecitin-induced demyelination	Mouse
[187]	Total brain	• (chronic EAE) ↓ BDNF, NRG1	EAE	Mouse
[162]	Microglia	• ↑ IBA1+ cells, BrdU-IBA1+ cells in the hippocampus	Cranial irradiation	Rat
[163]	Microglia	• ↓ IBA1+ cells	Neuropathic allodynia	Rat
[164]	Microglia	• ↓ MAC-2+ cells<br>• ↓ IL-17	EAE (PLP and MOG-induction)	Mouse
[165]	Microglia	• ↓ MAC-2+ cells	EAE	Mouse
[166]	Microglia	• ↓ MAC-1+ cells	EAE	Mouse
[167]	Microglia	• ↑ CD11c+ cells<br>• ↓ MAC-1+ cells	Double-transgenic (APP/PS1) AD model	Mouse
[168]	Microglia	• ↓ Amoeboid (activated) phenotype<br>• ↓ IL-10, TNFα	Primary fetal and adult microglia + activated T-lymphocyte co-cultures	Human
[169]	Microglia	• ↓ TNFα	BV-2 cell line	Mouse
[169]	Microglia	• ↓ IBA1-TNFα+ cells, IBA1+ density<br>• ↓ Microglia cell surface	EAE (tissue slices)	Mouse
[170]	Microglia	• ↑ MAC-1+ cells, CD68+ cells	Cuprizone-induced demyelination	Mouse
[170]	Microglia	• ↑ CD68+ cells, Biot-GSA+ cells<br>• ↑ IL-4, IL-10	Primary cultures	Rat
[171]	Microglia	• (high concentrations) ↑ Cytotoxicity<br>• ↑ Phagocytic activity of latex beads<br>• ↑ IL-10<br>• ↓ TNFα<br>• No effect on NO production	Primary cultures	Rat
[172]	Microglia	• ↑ Bacterial phagocytosis	Primary cultures (IFN-y-stimulated)	Not mentioned
[173]	Microglia	• No effect on phagocytosis of autologous peripheral blood-derived mononuclear cells	Primary cultures	Human
[174]	Microglia	• ↑ IL-10, TGF-β2	EAE (GA-T-cell induced cell injection)	Mouse
[176]	Microglia	• ↓ IBA1+ cells	EAE	Mouse
[179]	Microglia	• ↓ CD45+ cells	EAE	Mouse
[160]	Microglia	• (pre-EAE treatment) ↓ CD45+ cells	EAE	Rat
[162]	Astrocyte	• No effect on BrdU-GFAP+ cells in the hippocampus	Cranial irradiation	Rat
[174]	Astrocyte	• ↑ IL-10, TGF-β2	EAE (GA-treated T-cell cell injection)	Mouse
[176]	Astrocyte	• ↓ GFAP+ cells	EAE	Mouse
[177]	Astrocyte	• ↑ BDNF<br>• ↑ *Bdnf*	R6/2 and YAC128 HD models	Mouse

[177]	Astrocyte	• ↑ BDNF	Primary mesencephalic cultures	Mouse
[179]	Astrocyte	• No effect on GFAP+ cells	EAE	Mouse
[160]	Neuron (RGC)	• ↓ Neurodegeneration<br>• Improved Electroretinogram (ERG) response<br>• (pre-treatment) ↓ APP+ cells	EAE	Rat
[162]	Neuron	• ↑ BrdU/DCX+ neurons<br>• ↑ BrdU/NeuN+ neurons	Cranial irradiation	Rat
[166]	Neuron	• ↑ BrdU+ cells, DCX+ cells<br>• ↓ Neuronal damage<br>• ↑ Migration of neuronal progenitor cells (to lesion sites)<br>• ↑ DCX-BDNF+ cells	EAE	Mouse
[167]	Neuron	• ↑ BrdU-NeuN+ cells<br>• ↑ DCX+ cells	Double-transgenic (APP/PS1) AD model	Mouse
[169]	Neuron	• ↓ Decay time, half width of spontaneous excitatory postsynaptic currents (sEPSCs	EAE (slice cultures)	Mouse
[175]	Neuron	• ↑ *Nrg1*, *Ninj1*, *Snap25*, *Grin1*	EAE	Mouse
[176]	Neuron	• ↑ NeuN+ cells<br>• ↓ RIP3 (necroptotic)/NeuN+ cells	EAE	Mouse
[177]	(motor) Neuron	• ↓ clCASP3+ cells	Primary cells (treated with GA-astrocyte medium)	Mouse
[177]	Neuron	• ↓ Cresyl Violet+ cells	R6/2 and YAC128 HD models	Mouse
[179]	Neuron	• ↓ β-APP+ axons<br>• ↑ NF-200+ cells<br>• ↑ Callosal action potential (CAP) response	EAE	Mouse
[180]	Neuron	• ↓ Bielschowsky staining<br>• ↓ APP+ cells<br>• ↓ Non-phosphorylated neurofilaments (SMI-32)+ cells	EAE	Mouse
[181]	Neuron	• ↓ SMI32+ cells<br>• (BDNF-/- animals) Less effect on SMI32+ cells	EAE	Mouse
[182]	Neuron (RGC)	• ↓ Cell death	Organophosphate intoxication	Mouse
[183]	Neuron	• ↓ Axonal loss (SEM)	EAE	Mouse
[184]	Neuron	• ↑ Axonal integrity<br>• ↑ NAA/ Cr ratio	^{1}H-MRS in MS-patients	Human
[188]	Neuron	• ↑ Axonal diameter<br>• No effect on BDNF	EAE	Mouse
[160]	Oligodend rocyte	• ↑ LFB+ cells	EAE	Rat
[164]	Oligodend rocyte	• ↓ MBP-IL-17+ cells	EAE (PLP and MOG-induction)	Mouse
[165]	Oligodend rocyte	• ↓ Demyelination	EAE	Mouse
[170]	Oligodend rocyte	• (pre- and concomitant) ↓ Eriochrome Cyanine staining<br>• (pre- and concomitant) ↑ Sudan Black<br>• (pre- and concomitant) ↑ Myelin lamellae structure<br>• (pre- and concomitant) ↓ PDGFRα+ cells<br>• (pre- and concomitant) ↑ CC1+ cells	Cuprizone-induced demyelination	Mouse

		• No effect on g-ratio		
[170]	Oligodend rocyte	• ↑ PDGFRα+ cells<br>• ↑ MBP+ cells	Primary cultures (stimulated with GA-treated microglia medium)	Rat
[175]	Oligodend rocyte	• ↑ *Mbp, Olig2*	EAE	Mouse
[176]	Oligodend rocyte	• ↑ MBP+ cells	EAE	Mouse
[179]	Oligodend rocyte	• ↑ MBP+ intensity, CC1+ cells, OLIG2+ cells, PLP1	EAE	Mouse
[180]	Oligodend rocyte	• ↑ Luxol Fast Blue (LFB staining)	EAE	Mouse
[183]	Oligodend rocyte	• (GA concomitant with EAE)<br>↓ Demyelination<br>• (GA (not) concomitant with EAE)<br>↑ MBP+ cells<br>• (GA not concomitant with EAE)<br>↑ NG2-BrdU+ cells<br>• (GA not concomitant with EAE)<br>↑ O4-BrdU+ cells	EAE	Mouse
[186]	Oligodend rocyte	• ↑ PDGFRα-Ki67+ cells	Embryonic forebrain cultures (GA-reactive T-cell medium treated)	Mouse
[186]	Oligodend rocyte	• ↓ Eriochrome cyanine staining	Lysolecitin-induced demyelination	Mouse
[188]	Oligodend rocyte	• ↑ Myelinated axons (MBP-NFL+ cells)<br>• ↑ IGF-1+ cells, BDNF+ cells<br>• ↑ NG2+ cells in white matter and grey matter, NG2-BrdU+ cells, OLIG2-BrdU+ cells, APC+ cells<br>• ↑ Myelin thickness, myelin diameter<br>• ↓ G-ratio	EAE	Mouse
[189]	Oligodend rocyte	• ↑ O4+ cells, OLIG2+ cells, CNPase+ cells	Primary cultures (GA-reactive T-lymphocyte (Th1 and Th2) stimulated)	Rat
[189]	Oligodend rocyte	• ↑ OLIG2+ cells	Primary cultures (GA-reactive T-lymphocyte (Th1 and Th2) stimulated)	Human

Supplementary table 4. Molecular effects of Interferon-β (IFN-β) and Interferon-alpha (IFN-α) on microglia, astrocytes, neurons and oligodendrocytes. ↑ indicates increased level of expression, number of cells or morphological/functional state; ↓ indicates reduced expression level, number of cells or morphological/functional state. Gene name in *italics* indicates mRNA expression; gene name in regular font indicates protein expression.

**Refe-rence**	**Com-pound**	**Cell type**	**Effect**	**Model**	**Species**
[206]	rhIFN-α	Total brain	• ↑ pJAK1, pSTAT1, TRAF3<br>• ↓ pNFκB, IL-6, TNFα	**Germinal** matrix hemorrhage (GMH)	Rat
[208]	IFN-β-1a	Total brain	• ↑ myo-inositol (mI), total creatine (tCr), total choline (tCho)	RRMS-patients	Human
[220]	IFN-β<br>IFN-β+B12 vitamin	Total brain	• ↑ STAT1	ND4 demyelination model	Mouse

[251]	IFN-β	Total brain	• ↓ *Il-17, Il-6, Foxp3*	EAE	Mouse
[202]	IFN-β-1	Microglia	• ↓ CXCL13<br>• ↑ CCL2	Interferon regulatory factor-7 (IRF7)-/- cultures (stimulated)	Mouse
[204]	IFN-β<br>IFN-α	Microglia	• (IFN-β) ↓ MMP-2, MMP-9<br>• (IFN-β) ↓ *Mmp-2, Mmp-9*<br>• ↓ MMP-2, MMP-9	Primary cells cultures (LPS-stimulated)	Rat
[205]	IFN-β	Microglia	• ↓ Activated IBA1+ cells	Age-related macular degeneration model	Mouse
[206]	rhIFN-α	Microglia	• ↓ IBA1+ cells<br>• ↓ Soma size	**Germinal** matrix hemorrhage (GMH)	Rat
[207]	IFN-β-1a	Microglia	• ↓ ED-1+ cells	MOG-induced optic neuritis	Rat
[209]	rIFN-β	Microglia	• ↑ *Aif1, B2m, Cst7, Spp1, Il-1α*<br>• ↑ CD68+, nuclear STAT1<br>• ↓ Dendrite length<br>• ↑ *C1qa, C3, C4b*<br>• ↑ CD3C3 complement-dependent synapse elimination	AD model and wildtype animals (slice cultures)	Mouse
[209]	rIFN-β	Microglia	• ↑ pSTAT1, C3d	Mixed glial cultures	Mouse
[210]	mIFN-β	Microglia	• ↑ IBA1+ cells	GBM8-Fluc-implanted athymic nude mice	Mouse
[211]	poly(I:C) (IFN-β inducer)	Microglia	• ↓ Ki67+ cells<br>• ↑ *Ifn-β, Irf3, Irf, Irf9, Ifit1, Ifitm3, Il-1β, Tnfα, Il-6*<br>• ↑ *Cd45, Cd11b*<br>• ↑ vulnerability of microglia to stress in offspring	Maternal immune activation	Mouse
[211]	IFN-β	Microglia	• ↓ Ki67+ cells<br>• (after maternal separation) ↑ TNFα, CD45+ cells, IBA1+ cells, CX3CR1+ cells, CD45-Cd11b+ cells	Maternal immune activation	Mouse
[212]	IFN-β	Microglia	• ↓ Proliferation (3^H incorporation)<br>• ↑ MHC-11+ cells, FcR<br>• (LPS-stimulated) ↑TNFa	Primary cultures	Rat
[213]	IFN-β	Microglia	• ↑ Activated morphology<br>• ↑ IBA1+ cells, IBA1-CD16/32+ cells, IBA1-CD206+ cells, IBA1-pNfkB+ cells, IBA1-pSTAT1+ cells, IBA-CXCL10+ cells<br>• ↑ *Il-12, Il-1β, Tnfα, Il-6, Ccl5, Cxcl9, Cxcl10*<br>• ↓ *Cd163, Il-4, Mcr1, Ccr7, Il-10, Il-13, Tgf-β*<br>• ↑ CXCL9, CXCL10, CCL5	Primary cultures	Mouse
[214]	IFN-α	Microglia	• ↑ MHC-II+ cells, CD86+ cells, CD54+ cells	Primary cultures	Mouse
[215]	IFN-β<br>+TNFα	Microglia	• ↑ CCL5<br>• ↑ pJAK1, pTYK2<br>• ↑ pSTAT1α/β	MG6-1 cell line	Mouse
[216]	IFN-β	Microglia	• ↑ TNFα, IL-1β<br>• ↑ NO<br>• ↓ Superoxide anions, Glutamate	Primary cultures (LPS-stimulated)	Mouse
[217]	IFN-α	Microglia	• ↑ *Ifit3, Ifit1, oas1, mx1, ifi35, stat2, psmb8, stat1, tap1, Dhx58, Ifih1, Irf7, Ddx58, Zbp1, Stat2, Stat1, Adar, Ifit2, Psmb9, H2-T23, H2-*	Primary cultures	Mouse

			Q7, Psmb8, Tap1, Tapbp, H2-Q1, Oas1d, Ifih1, Oas1, Irf7, Oas2, Ddx58, Oas1b, Eif2ak2, Tlr3, Dhx58, Ifih1, Irf7, Ddx58, Trim25, Zc3hav1, Parp11, Parp12, Parp9, Parp14, H2-T23, H2-Q7, H2-T10, H2-D1, H2-q8, Psmb9, Usp18, Psmb10, H2-Q7, Psme2, Psmb8, Tap1, Ube2l6, H2-Q1, Cxcl10, H2-T23, H2-Q7, Tlr3, H2-D1, Eif2ak2, Tlr3, Stat1, Il18, Cd40, Il1rn, Il15, Cd86, Ccl5, Il2rg, Il15, Axl, Tnfsf10, Irf1, Parp14, Cxcl9, Ccl2, Cd40, Kdr, Mmp13, Ccl5, C3, Cd180, Cd40, Pik3ap1, Itpr1, Daxx, Il18, Ddit3, Il1rn, Enpp1, Pank3, Epas1, Mt2a, Gls, Gclm, Gstm5, Gclm, Sult1a1, Igfbp4, Prkar2, Gpr183, Prkar2b, Ednrb, Fzd1, Adrb2<br>• More extensive response than astrocytes		
[218]	IFN-α	Microglia	• ↑ *Tnfα, Ifit2, Il-1α, Il-6, Tnfsf10, Ccl2, Ccl3, Ccl4, Cxcl11, Ifit1, Ifit3, Isg20, Ifit1bl1, Usp18, Tgtp1, Pyhin1, Klrk1, Gm4951, Irf7, Phf11a, Serpina3g, Phf11d, Serpin3af, Mx2, Oas3, Zbp1, Pydc3, Phf11b, Oasl2, Apol9a, Bc094916, Ms4a4c, Fam26f, Ifi204, Gbp11, Pydc4, Fgl2, Batf2, Herc6, Gbp9, Bc147527, Slfn1, Slfn9, Slfn4, Gm12185, Irgm1, Slfn5, Trim30d, Stat1, Ifih1, Gm7609, Ddx60, Oas2, Mnda, H2-T24, Ifi44, Apol9b, Ifi203, Mndal*	Primary cultures	Mouse
[219]	IFN-β	Microglia	• ↑ *Ifi208, Ifi213, p204, Ifi205, Ifi206, Ifi207, Ifi202Ifi214, Aim2, cGas*	Primary cultures	Mouse
[204]	IFN-β<br>IFN-α	Astrocyte	• (IFN-β) ↓ MMP-2, MMP-9<br>• (IFN-β) ↓ *Mmp-2, Mmp-9*<br>• ↓ MMP-2, MMP-9	Primary cultures (LPS-activated)	Rat
[209]	rIFN-β	Astrocyte	• ↑ C3-GFAP+ cells	Wildtype animals	Mouse
[209]	rIFN-β	Astrocyte	• ↑ pSTAT1, C3d	Mixed glial cultures	Mouse
[217]	IFN-α	Astrocyte	• ↑ *Ifit3, Ifit1, oas1, mx1, ifi35, stat2, psmb8, stat1, tap1, Dhx58, Ifih1, Irf7, Ddx58, Zbp1, Stat2, Stat1, Adar, Ifit2, Psmb9, H2-T23, H2-Q7, Psmb8, Tap1, Tapbp, H2-Q1, Oas1d, Ifih1, Oas1, Irf7, Oas2, Ddx58, Oas1b, Eif2ak2, Tlr3, Dhx58, Ifih1, Irf7, Ddx58, Trim25, Zc3hav1, Parp11, Parp12, Parp9, Parp14, H2-T23, H2-Q7, H2-T10, H2-D1, H2-q8, Psmb9, Usp18, Psmb10, H2-Q7, Psme2, Psmb8, Tap1, Ube2l6, H2-Q1, Cxcl10, H2-T23, H2-Q7, Tlr3, H2-D1, Eif2ak2, Tlr3, Stat1, H2-M3, Tap2, H2-K1, Tgm2, Vnn1, Parp3, Cybb, Apod*<br>• Less extensive response than microglia	Primary cultures	Mouse
[219]	IFN-β	Astrocyte	• *Ifi203, Ifi208, Ifi213, p204, Ifi205, Ifi206*	Primary cultures	Mouse
[220]	IFN-β<br>IFN-β+B12 vitamin	Astrocyte	• ↓ GFAP+ cells	EAE	Mouse
[222]	IFN-β	Astrocyte	• ↑ *Ifnar1, Ahr, Cyp1b1, Mx1, Stat1, Stat2, Irf9, Il-10, Mx1*<br>• ↓ *Vim, Csf1, Csf2, Ccl2*<br>• ↑ pSTAT1, pSTAT2, nuclear p65	EAE	Mouse

[222]	IFN-β	Astrocyte	• ↑ *STAT1, STAT2, IRF9, MX1, AHR*	Fetal cell cultures	Human
[223]	IFN-β	Astrocyte	• ↓ Proliferation rate	Primary cultures (growth factor and cytokine-stimulated)	Rat
[224]	IFN-β	Astrocyte	• No effect on proliferation (S-phase)<br>• ↑ *2-5A Synthetase*	Glioma cell lines (AO2V4, GJC, G JR, NN, NNR) and primary cultures	Human
[225]	IFN-β-1b	Astrocyte	• ↓ Nitrite production	A172 cell line	Human
[226]	IFN-β	Astrocyte	• ↓ MBP-cleaving proteolytic activity<br>• ↓ CANP-2, MMP-2,MMP-9<br>• ↓ *Mmp-9, Timp-1, Mmp-2, Timp-2, Canp-2*	Primary cultures (LPS-stimulated)	Rat
[227]	IFN-β-1b	Astrocyte/Endothelial cell	• ↓ Permeability for inulin and sucrose	Co-cultures HBMEC with rat astrocytes	Human / Rat
[228]	IFN-β	Astrocyte/Endothelial cell	• ↓ Permeability for 3H-inulin and 14C-sucrose	Co-cultures brain endothelial cells with astrocytes	
[229]	IFN-β	Astrocyte	• (IFN-β) ↑ GSDMT1, COF1, PROF1, UBIQ, SBP1, GDIB, GANAB, CAP1, AMPL, GFAP, TBB2A, TBB2B, G3P, VINC, NIT2, PSME1, IDHC, PDIA3, TCPA, ECH1, DPY2, VIME<br>• (LPS+IFN-β) ↑ACTN4, DPYL2,G6PD, PSME2, GSTP1, PROF1, NADP, IDHC, VIME, PDIA3	Primary cells (LPS-stimulated)	Rat
[230]	IFN-β	Astrocyte	• ↑ GFAP intensity<br>• ↑ IL-6,CCL5, CXCL10	Primary cultures	Mouse
[231]	IFN-β	Astrocyte	• ↑ MHC-I	NG97 cell line	Human
[232]	IFN-β via poly I:C induction	Astrocyte	• ↑ CXCL10	EAE	Mouse
[233]	IFN-α	Astrocyte	• ↑ MCP-1, IL-6, IP-10	Co-cultures monocyte and astrocytes (U-251 cell line and fetal astrocytes)	Human
[234]	IFN-β	Astrocyte	• ↑ Apoptosis<br>• ↑ pP38 MAPK<br>• ↓ TNFα-induced apoptosis	Fetal cell cultures (serum-starved or cytokine-stimulated)	Rat
[235]	IFN-β	Astrocyte	• ↓ Apoptosis	Primary fetal cultures (serum-starved)	Rat
[235]	IFN-β	Astrocyte	• No effect on apoptosis<br>• ↑ pAKT	Primary neonatal cultures (serum-starved or sodium butyrate treated)	Rat
[236]	IFN-β	Astrocyte	• (low dose) ↑ BrdU+ cells<br>• (low dose) ↑ pAkt<br>• (high dose) ↑ Cell death<br>• (high dose) ↓ pP38 MAPK, Iκ-B<br>• (high dose) ↑ WIP1	Fetal cell cultures (serum-starved)	Rat

[237]	IFN-β	Astrocyte	• ↑ pPI3K, pAKT	Primary fetal cultures (serum-starved)	Rat
[160]	IFN-β-1b	Neuron (retinal ganglion cells; RGC)	• No effect on cell survival	EAE	Rat
[185]	IFN-β	Neuron	• No effect on N-acetyl aspartate (NAA), creatine (Cr), phosphocreatine (pCr) and glutamate (Glut)	RRMS-patients	Human
[207]	IFN-β-1a	Neuron (RGCs)	• ↓ Loss of RGCs<br>• ↓ β-APP+ axons	MOG-induced optic neuritis	Rat
[207]	IFN-β-1a	Neuron (RGCs)	• No effect on neuroprotection<br>• ↑ pMAPK1/2	Primary cultures	Rat
[209]	rIFN-β	Neuron	• ↓ PSD95<br>• ↓ Dendritic spine density<br>• ↑ C3 complement-dependent synapse elimination	Wildtype animals	Mouse
[216]	IFN-β	Neuron	• No effect on MAP2+ cells	Primary cultures (LPS-stimulated)	Mouse
[216]	IFN-β	Neuron	• (LPS stimulated) ↓ activated microglia induced neuronal cell death<br>• No decreased cell death after NDMA or AMPA stimulation	Co-cultures neurons and microglia	Mouse
[238]	IFN-β	Neuron	• No effect on NAA/Cr	RRMS-patients	Human
[239]	IFN-β-1b	Neuron	• ↑ NAA/Cr ratio	MS-patients	Human
[240]	IFN-β-1b	Neuron	• ↑ NAA/Cr ratio	MS-patients	Human
[241]	IFN-α2	Neuron	• ↑ Cr, Lactate, Myo-inositol, Taurine, Scyllo-inositol, Glycerophosphorylcholine,<br>• ↑ Lactate, Glycine, Glutamine, Acetate, Ethanol in medium	SH-SY5Y cell line	Human
[242]	IFN-β	Neuron (DRG)	• ↑ Cell viability<br>• ↓ HSV-1 virus production<br>• ↓ *Icp0, Tk, gB*<br>• ↑ pJAK, pSTAT1	Primary cultures (HSV-1 infected)	Mouse
[243]	IFN-α-2/4<br>IFN-β-1	Neuron	• ↑ Cell survival<br>• ↑ β-IIITUB+ cells	Cerebral organoids (La Crosse Virus infected)	Human
[244]	IFN-β	Neuron	• ↑ *Oasl2*<br>• No effect on protection from viral infection<br>• ↑ STAT1, MX1<br>• ↑*Usp18, Rtp4, Ifit4, Ifi44,Isg15, Mpa21, Mx2, Oasl2, Xaf1, Bst2, Gbp3, Iigp1, Ifi2712a*<br>• Low expression of IFN-responsive genes in neurons: *Dhx58, Gvin1, Sp100, Ifi203 isoforms 1 and 2, Irgm2, Lgals3bp, Ifi205, Apol9b, Ifi204, Ifi202b, Tor3a, Slfn2, Ifi35, Lgals9*	Primary cultures	Mouse
[244]	IFN-β	Neuron	• ↑ STAT1	N2A cell line	Mouse
[245]	IFN-α	Neuron	• ↓ Firing rate	Slice cultures	Rat
[246]	IFN-β	Neuron	• ↓ Synaptophysin (SYP) signal intensity<br>• ↑ MHC-I signal intensity	PC12 cell line	Rat

[247]	IFN-β	Neuron	• SNORA31- and TLR3- but not STAT1-mutated neurons resistant to HSV-1	Induced Pluripotent Stem cell (IPSC)-derived neurons	Human
[207]	IFN-β-1a	Oligodendrocyte	• ↑ Luxol Fast Blue staining	MOG-induced optic neuritis	Rat
[220]	IFN-β<br>IFN-β+B12 vitamin	Oligodendrocyte	• ↑ Cell size and process length<br>• ↓ aNOTCH-1, JAG-1	MO3.13 cell line	Human
[220]	IFN-β<br>IFN-β+B12 vitamin	Oligodendrocyte	• ↓ aNOTCH-1<br>• ↑ SHH	Primary cultures	Rat
[220]	IFN-β<br>IFN-β+B12 vitamin	Oligodendrocyte	• ↑ PLP1	ND4 demyelination model	Mouse
[248]	IFN-β	Oligodendrocyte	• ↓ Demyelination	MOG35-55 (EAE)<br>MBP35-47 (EAE)<br>PLP190-209 (EAE)<br>HSV-IL-2	Mouse
[249]	IFN-β	Oligodendrocyte	• ↑ A2B5-BrdU+ cells<br>• No effect on differentiation	Mixed glial cultures (IFN-β MS-patient sera treated)	Rat
[250]	IFN-β	Oligodendrocyte	• ↓ A2B5-GALC+ cell ratio<br>• No effect on proliferation	Mixed glial cultures	Rat
[250]	IFN-β	Oligodendrocyte	• No effect on differentiation	Oligodendrocyte precursor cell (OPC) primary cultures	Rat
[250]	IFN-β	Oligodendrocyte	• No cytoprotective effect	CG4 cell line (H2O2, NO, complement, Glutamate stimulated or astrocyte medium-treated)	Rat
[250]	IFN-β	Oligodendrocyte	• No toxicity	OLN-93 cell line	Rat

Supplementary table 5. Molecular effects of Teriflunomide (TF) on microglia, neurons and oligodendrocytes. ↑ indicates increased level of expression, number of cells or morphological/functional state; ↓ indicates reduced expression level, number of cells or morphological/functional state. Gene name in *italics* indicates mRNA expression; gene name in regular font indicates protein expression.

**Reference**	**Cell type**	**Effect**	**Model**	**Species**
[61]	Total brain	• No effect on pathology	EAE	Mouse
[255]	Total brain	• ↑ cAMP, GDP<br>• ↓ UDP, UMP, ADP, AMP<br>• ↑ Taurine, Glutamic Acid<br>• ↓ GSH	Wildtype animals	Mouse
[49]	Microglia	• ↓ SIGLEC-1+ cells	Neuronal Ceroid Lipofuscinosis (CLN)	Mouse
[146]	Microglia	• ↓ CXCL10, IL-6, CCL2	HMC3 cell line – monocyte co-culture (HIV-particle-induced)	Human

[254]	Microglia	• ↓ IBA1+ cells<br>• ↓ IL-1β, COX2, 3-NT (3-Nitrotyrosine)	Transient middle cerebral artery occlusion (tMCAO)	Mouse
[256]	Microglia	• ↓ IBA1+ cells with activated phenotype	Traumatic Brain Injury (TBI)	Rat
[257]	Microglia	• ↓ IBA1+ cells in corpus callosum	Theiler's murine encephalomyelitis virus (TMEV)-induced demyelination	Mouse
[258]	Microglia	• ↓ CD86+ cells<br>• No effect on morphology<br>• (GM-CSF treatment) ↑ BrdU+ cells<br>• No effect on *iNos, Il-1β, Tnfα, Arg1, Igf1*<br>• ↑*Il-10*<br>• No effect on IκBα	Primary microglia and mixed glia cultures (LPS+IFN-y-stimulated)	Rat
[49]	Neuron	• ↓ Retinal thinning<br>• ↓ SMI32+ axonal spheroids<br>• ↓ Loss of retinal ganglion cells (RGCs)	CLN	Mouse
[146]	Neuron	• ↓ Neurotoxicity of microglia-monocyte co-culture medium (MAP2-PI+ cells)	Fetal cell cultures	Human
[254]	Neuron	• ↑ BrdU/DCX+ cells<br>• ↑ MASH1, DCX, PBX1 expression in SVZ	tMCAO	Mouse
[256]	Neuron	• ↑ DCX+ cells	TBI	Rat
[257]	Neuron	• ↓ Axonal loss	TMEV-induced demyelination	Mouse
[257]	Oligoden drocyte	• No effect on OLIG2+ cells, CC1+ cells, CC1-OLIG2+ cells	TMEV-induced demyelination	Mouse
[259]	Oligoden drocyte	• (high dose) ↑ ClCASP3+ cells<br>• ↓ Ki67+ cells<br>• (short term pulse) ↑ *Cnp, Tap73, Mash1, Myrf, Nkx2.2, Plp1*<br>• (short term pulse) ↑ CNPase+ cells, MOG+ cells, MBP+ cells, Tap73<br>• (short term pulse) ↑ MBP-positive internodes	Co-cultures neuron and oligodendrocyte	Rat

Supplementary table 6. Molecular effects of Laquinimod (LQ) on microglia, astrocytes, neurons and oligodendrocytes. ↑ indicates increased level of expression, number of cells or morphological/functional state; ↓ indicates reduced expression level, number of cells or morphological/functional state. Gene name in *italics* indicates mRNA expression; gene name in regular font indicates protein expression.

**Refe-rence**	**Cell type**	**Effect**	**Model**	**Species**
[263]	Total tissue	• (LQ after disease onset and simultaneously with disease onset)<br>• ↓ *Cd68, iNos, Il-1β, Myd88, MiR-124a*	Experimental Autoimmune Encephalitis (EAE)	Mouse
[268]	Total tissue	• ↓ CASP3+ cells	Cuprizone-induced demyelination	Mouse
[277]	Total tissue	• ↑ BDNF	Wildtype animals	Mouse
[261]	Microglia	• ↓ IBA1+ cells, TMEM119-IBA1+ cells, F4/80-IBA1+ cells<br>• ↓ *Iba1, Tmem119, Cd68*	EAE	Mouse
[262]	Microglia	• ↓ IBA1+ cells<br>• (AhR-/- animals) No effect on IBA1+ cells	EAE	Mouse

[263]	Microglia	• ↓ CD14+ cell area<br>• ↓ TNFα, IL-1β, IL-12p70, IL-6, IL-4, IL-10, Il-1RA, MMP-9<br>• ↑ G-CSF<br>• No toxicity<br>• ↓ pAKT, pJNK, pP90-Ribosomal S6 kinase<br>• ↑ MiR124a<br>• ↓ Neurotoxicity	Primary cultures (LPS-stimulated)	Human
[263]	Microglia	• ↓ TNFα	Primary cultures (LPS-stimulated)	Mouse
[263]	Microglia	• (LQ after disease onset) ↓ IBA1+ cells<br>• (LQ simultaneously with disease onset) ↓ IBA1+ cells	EAE	Mouse
[263]	Microglia	• ↓ Nitrite production	Co-cultures microglia and neurons	Mouse
[264]	Microglia	• ↓ IBA1+ cells	Cuprizone-induced demyelination	Mouse
[265]	Microglia	• ↓ IBA1+ cells, IBA1+ intensity, MAC-3+ cells<br>• ↑ TSPO-ligand binding	Cuprizone-induced demyelination	Mouse
[266]	Microglia	• ↓ CD45+ cells	EAE	Mouse
[267]	Microglia	• ↓ MAC-3+ cells	Wildtype, TLR4-/- and MyD88-/- demyelination models	Mouse
[268]	Microglia	• ↓ MAC-3+ cells	Cuprizone induced demyelination (wildtype and RAG1-/- animals)	Mouse
[268]	Microglia	• ↑ CCL5<br>• No effect on NFκB	Primary cultures (LPS- or TNFα-stimulated)	Mouse
[269]	Microglia	• ↓ IB4+ cells	EAE	Mouse
[271]	Microglia	• ↓ *Hspa1a, Nedd1, Epn2, Bach2, Tnfrsf17, Sult1a1*<br>• ↑ *Chi3l3, Atf3, Adamts1, Fosb, Cybb, Ccl3, Ccl4, Ccl2, Fos, Tnf, CXcl10, Egr1, C4a, Jun, Lilrb4, Tspo*	Traumatic Brain Injury (TBI)	Mouse
[261]	Astrocyte	• (LQ during EAE) ↑ GFAP+ area<br>• (LQ after EAE) ↓ GFAP+ area, VIM+ area	EAE	Mouse
[262]	Astrocyte	• ↓ GFAP+ cells<br>• (AhR-/- animals) No effect on GFAP+ cells	EAE	Mouse
[265]	Astrocyte	• ↓ GFAP+ intensity, VIM+ intensity, ALDH1L1+ intensity	Cuprizone-induced demyelination	Mouse
[266]	Astrocyte	• ↓ GFAP+ cells	EAE	Mouse
[267]	Astrocyte	• ↓ NFκB activation	Primary cultures (IL-1β/IFNy stimulated) of TRL4-/-, MyD88-/- and TRIF-/- animals	Mouse
[268]	Astrocyte	• ↓ GFAP+ reactive fibers<br>• ↓ NFkB	Cuprizone-induced demyelination (wildtype and RAG1-/- animals)	Mouse
[268]	Astrocyte	• ↓ *Il-12 p35, Il-23 p19, Tnfα, IFNα, Cxcl10*<br>• ↑ *Ccl5*<br>• ↓ TNFα, Nitrite, CXCL10, IL-6<br>• ↑ CCL5<br>• ↓ NFκB<br>• ↓ Nuclear translocation p65	Primary cultures (IL-1β-stimulated)	Mouse

[269]	Astrocyte	• ↓ No effect on GFAP+ cells or morphology	EAE	Mouse
[273]	Astrocyte	• ↓ IL-6, IP-10<br>• ↑ RANTES	Primary cultures (IL-1β/IFN-y-stimulated)	Human
[261]	Neuron (retinal ganglion cells; RGC)	• (LQ during EAE) ↓ BRN3A-ClCASP3+ cells<br>• (LQ during EAE) ↑BRN3A+ cells<br>• (LQ during EAE) ↑ *Brn3a*<br>• (LQ after EAE) No effect on BRN3A+ cells	EAE	Mouse
[262]	Neuron	• ↓ APP+ cells<br>• (AhR-/- animals) No effect on APP+ cells	EAE	Mouse
[263]	Neuron	• (LQ simultanousely with disease onset) ↓ Axonal injury (Bielchowsky silver staining)<br>• (LQ after disease onset) ↓ Axonal loss (Bielchowsky silver staining)	EAE	Mouse
[263]	Neuron	• ↑ MAP2+ cells	Co-cultures microglia and neurons	Human
[263]	Neuron	• ↑ MAP2+ cells	Co-cultures microglia and neurons	Mouse
[264]	Neuron	• ↓ APP+ cells	Cuprizone-induced demyelination	Mouse
[265]	Neuron	• ↓ APP+ spheroids, VGLUT1-APP+ spheroids, SYP+ spheroids	Cuprizone-induced demyelination	Mouse
[266]	Neuron	• ↑ Callosal axon conduction<br>• ↑ Axon refractoriness<br>• ↓ APP-NF200+ cells	EAE	Mouse
[267]	Neuron	• ↓ APP+ axons	Wildtype, TLR4-/- and MyD88-/- demyelination	Mouse
[268]	Neuron	• ↓ APP+ axons	Cuprizone-induced demyelination (wildtype and RAG1-/- animals)	Mouse
[269]	Neuron	• ↓ Axonal damage<br>• Modulation EAE-induced GABAergic synapse alterations<br>• ↓ Glutamate excitotoxicity<br>• ↑ Cannabinoid receptor (CB1) sensitivity<br>• ↑ Inhibitory post-synaptic currents<br>• ↓ Excitatory post-synaptic currents	EAE	Mouse
[271]	Neuron	• ↓ APP+ cells<br>• ↑ DCX+ cells	TBI	Mouse
[273]	Neuron	• No effect on NSC viability<br>• No effect on NSC migratory response to CXCL12, CXCL8<br>• No effect on neuronal differentiation Nestin+ or DCX+ cells	WA09 embryonic stem cell line (IL-1β-stimulated)	Human
[275]	Neuron	• ↓ Cresyl Violet+ cells, iNOS+ cells<br>• ↑ NeuN+ cells, DARPP-32, BDNF-NeuN+ cells<br>• ↑ *Bdnf*	R6/2 Huntington’s disease model	Mouse
[275]	Neuron	• ↓ Mutant huntingtin (MHTT)+ cells<br>• ↓ Ubiquitin+ cells<br>• (Ponasterone A treated) ↓ Basal respiration, ATP production, proton leak	PC12 cell line	Rat

[276]	Neuron	• ↓ BAX, clCASP6	Primary cultures (induced DNA damage)	Mouse
[261]	Oligodendrocyte	• (LQ during EAE) ↑ LFB staining<br>• (LQ during EAE) ↑ MBP+ area<br>• (LQ during EAE) No effect on LFB staining	EAE	Mouse
[262]	Oligodendrocyte	• ↑ Luxol Fast Blue (LFB) staining	EAE	Mouse
[263]	Oligodendrocyte	• (LQ after disease onset) ↑ LFB staining	EAE	Mouse
[264]	Oligodendrocyte	• ↑ OLIG2+ cells, APC+ cells<br>• ↑ Myelination<br>• ↑ PLP1/MAG+ cells	Cuprizone-induced demyelination	Mouse
[265]	Oligodendrocyte	• ↑ LFB staining	Cuprizone-induced demyelination	Mouse
[266]	Oligodendrocyte	• ↑ MBP+ cells, MBP-NF200+ cells (myelinated axons), PLP1+ cells, OLIG2-Ki67+ cells, CC1+ cells	EAE	Mouse
[267]	Oligodendrocyte	• ↑ P25+ cells	Wildtype, TLR4-/- and MyD88-/- demyelination	Mouse
[268]	Oligodendrocyte	• ↑ LFB staining<br>• ↑ # myelin sheaths<br>• ↓ Apoptosis	Cuprizone-induced demyelination (wildtype and RAG1-/- animals)	Mouse
[268]	Oligodendrocyte	• No effect on survival and mitochondrial respiration	Primary OPC cultures	Mouse
[269]	Oligodendrocyte	• ↓ Demyelination	EAE	Mouse
[273]	Oligodendrocyte	• No effect on OPC viability<br>• Minor effect on OPC proliferation<br>• No effect on OPC migratory response to CXCL8 or PDGF-AA<br>• No effect on OPC differentiation (NG2+, CNPase+, MBP+ cells).	WA09 embryonic stem cell line (IL-1β-stimulated)	Human
[278]	Oligodendrocyte	• ↑ LFB staining<br>• ↑ MBP+ myelin sheaths	EAE	Marmoset

Supplementary table 7. Molecular effects of Natalizumab (NZ) on microglia, astrocytes, neurons and oligodendrocytes. ↑ indicates increased level of expression, number of cells or morphological/functional state; ↓ indicates reduced expression level, number of cells or morphological/functional state. Gene name in *italics* indicates mRNA expression; gene name in regular font indicates protein expression.

**Reference**	**Cell type**	**Effect**	**Model**	**Species**
[283]	Total brain	• ↓ Lipid peroxidases, Oxidized GSH<br>• ↓ Lipopolysaccharide binding protein (LBP)	EAE	Mouse
[284]	Total brain	• ↓ IL-12, IFNy	Double-transgenic (APP/PS1) AD model	Mouse
[284]	Microglia	• ↓ CD68+ cells, IBA1+ cells<br>• No effect on TMEM119+ cells	Double-transgenic (APP/PS1) AD model	Mouse
[285]	Microglia	• ↓ CD45(high)/CD11b+ cells	EAE	Mouse
[286]	Microglia	• ↓ 11C-PK11195 (TSPO) signal	MS-patients	Human
[287]	Microglia	• ↓ [11C]PK11195 (TSPO) signal	MS-patients	Human
[282]	Astrocyte	• ↑ SDF1-CXCL12+ cells	EAE	

[284]	Astrocyte	• ↓ GFAP+ cells, GFAP	Double-transgenic (APP/PS1) AD model	Mouse
[295]	Astrocyte	• ↓ GFAP-LCN2+ cells	EAE	Mouse
[185]	Neuron	• ↑ NAA, Cr, PCr, Glutamate	RRMS-patients	Human
[284]	Neuron	• ↓ PSD-95+ intensity	Double-transgenic (APP/PS1) AD model	Mouse
[285]	Neuron	• ↑ NF+ cells	EAE	Mouse
[285]	Oligoden drocyte	• ↑ MBP+ cells	EAE	Mouse

Supplementary table 8. Molecular effects of Alemtuzumab (AZ) on microglia and neurons. ↓ indicates reduced expression level, number of cells or morphological/functional state. Gene name in *italics* indicates mRNA expression; gene name in regular font indicates protein expression.

**Refe-rence**	**Cell type**	**Effect**	**Model**	**Species**
[301]	Microglia	• ↓ Dendrite length, ramification index	EAE	Mouse
[301]	Microglia	• ↓ Ramification index<br>• No change in microglia function	EAE (hippocampal slice cultures)	Mouse
[301]	Neuron	• No effect on NDMA-induced or Th17-induced excitotoxic Ca2+ levels	EAE (hippocampal slice cultures)	Mouse

Supplementary table 9. Molecular effects of Ocrelizumab (OCR) on microglia, astrocytes, neurons and oligodendrocytes. ↓ indicates reduced expression level, number of cells or morphological/functional state. Gene name in *italics* indicates mRNA expression; gene name in regular font indicates protein expression.

**Refe-rence**	**Cell type**	**Effect**	**Model**	**Species**
[208]	Total tissue/Neuro n	• ↓ Creatine, choline	RRMS-patients	Human
[71]	Microglia	• ↓ MHC-II+ cells<br>• ↓ [125I]DPA-713 (TSPO-ligand)	fDTH-EAE (experimental allergic encephalomyelitis)	Rat
[71]	Microglia	• ↓ MHC-II+ cells<br>• ↓ [125I]DPA-713 (TSPO-ligand)	MOG-induced EAE	Rat
[208]	Microglia	• ↓ Myo-inositol	RRMS-patients	Human
[305]	Microglia	• No effect on MAC-3+ cells	MOG-induced EAE	Mouse
[208]	Astrocyte	• ↓ Myo-inositol	RRMS-patients	Human
[305]	Oligodendro cyte	• No effect on Luxol Fast Blue staining	MOG-induced EAE	Mouse
